# Supplementary material for: Global hypo-methylation in a proportion of glioblastoma enriched for an astrocytic signature is associated with increased invasion and altered immune landscape
Source: eLife. 2022 Nov 22;11:e77335. doi: 10.7554/eLife.77335 (PMC9681209; doi:10.7554/eLife.77335)
Supplement: Figure 2—source data 1. [file elife-77335-fig2-data1.zip › Figure_2_source_data_1/Figure_2C/homerResults.html]

/data/Blizard-MarinoLab/Nicola\_Pomella/Motifs\_James/201117\_2// - Homer de novo Motif Results


# Homer *de novo* Motif Results (/data/Blizard-MarinoLab/Nicola\_Pomella/Motifs\_James/201117\_2//)

Known Motif Enrichment Results  
Gene Ontology Enrichment Results  
If Homer is having trouble matching a motif to a known motif, try copy/pasting the matrix file into
STAMP  
More information on motif finding results: HOMER
| Description of Results
| Tips
  
Total target sequences = 1176  
Total background sequences = 4892  
\* - possible false positive  

|  |  |  |  |  |  |  |  |  |
| --- | --- | --- | --- | --- | --- | --- | --- | --- |
| Rank | Motif | P-value | log P-pvalue | % of Targets | % of Background | STD(Bg STD) | Best Match/Details | Motif File |
| 1 | A C G T C G A T A C G T A T G C A G T C A C G T T C G A C A G T C A T G C G T A T C A G T G A C | 1e-27 | -6.221e+01 | 1.53% | 0.03% | 54.4bp (0.0bp) | Stat4/MA0518.1/Jaspar(0.708) More Information | Similar Motifs Found | motif file (matrix) |
| 2 | A G C T G T A C C A G T A G C T A C G T C G T A C G T A C A G T G T C A C G A T C G A T A C G T | 1e-23 | -5.363e+01 | 1.36% | 0.02% | 52.7bp (0.0bp) | PB0148.1\_Mtf1\_2/Jaspar(0.712) More Information | Similar Motifs Found | motif file (matrix) |
| 3 | C T A G C T A G A G T C T G A C G A T C T C G A T A C G T A G C A G C T A T C G A C G T C T A G T A C G A G C T A T G C C T G A T A C G T G C A T G A C A C T G T A G C C A T G T A G C T C A G T A C G C G A T T A G C G T C A C G T A A C T G T A C G C G T A A T C G T A G C T A C G T A G C C G A T A C T G T A C G T G A C G T C A T C A G T A G C G A T C C T A G | 1e-23 | -5.363e+01 | 1.36% | 0.03% | 47.0bp (38.7bp) | Plagl1/MA1615.1/Jaspar(0.443) More Information | Similar Motifs Found | motif file (matrix) |
| 4 | T G A C A C G T G T A C T C G A A T G C G C A T T A C G C T G A A C T G C T G A C G T A C G T A | 1e-21 | -4.943e+01 | 1.28% | 0.03% | 52.4bp (0.0bp) | PRDM1/MA0508.3/Jaspar(0.654) More Information | Similar Motifs Found | motif file (matrix) |
| 5 | C G A T T A G C G T C A C G T A C A T G T C G A T G A C C T G A C G T A C A G T C A T G C T A G G A T C G A C T G A T C G C T A T C A G G C T A C T A G C A T G G T C A G T C A C T G A C G A T A T C G G C A T T G A C C G A T C A T G T C A G G C T A C G A T A G C T A C G T C G A T C T A G G T C A C G A T C A G T C G T A A C G T C G T A C A T G A C G T C T G A | 1e-20 | -4.633e+01 | 1.45% | 0.06% | 47.1bp (23.2bp) | PU.1(ETS)/ThioMac-PU.1-ChIP-Seq(GSE21512)/Homer(0.422) More Information | Similar Motifs Found | motif file (matrix) |
| 6 | A C G T A T C G C T G A C A G T C G T A C T A G C T G A C T A G A G T C C G A T T G C A A T G C G C T A C G T A C A G T | 1e-19 | -4.529e+01 | 1.19% | 0.02% | 57.1bp (0.0bp) | ZNF415(Zf)/HEK293-ZNF415.GFP-ChIP-Seq(GSE58341)/Homer(0.651) More Information | Similar Motifs Found | motif file (matrix) |
| 7 | G A T C C G T A G A T C A G C T C G T A C G A T C G T A C G A T A C G T C G T A C G A T A C G T G C T A G A C T A C G T | 1e-18 | -4.277e+01 | 1.36% | 0.05% | 47.9bp (63.7bp) | PB0069.1\_Sox21\_1/Jaspar(0.686) More Information | Similar Motifs Found | motif file (matrix) |
| 8 | A C T G C T A G A G T C A C G T T C A G G T C A C T A G A G C T G T A C G A T C C G A T A G C T G T A C A G T C A G T C | 1e-17 | -4.123e+01 | 1.11% | 0.01% | 55.4bp (0.0bp) | ZNF467(Zf)/HEK293-ZNF467.GFP-ChIP-Seq(GSE58341)/Homer(0.630) More Information | Similar Motifs Found | motif file (matrix) |
| 9 | T A G C T C A G A T G C T A G C T A C G T G A C A G T C A T G C A T G C A T G C A T G C G A C T C A T G T A C G A T C G T A C G C A G T A T C G A T G C T C A G G T A C T A C G T A G C C T G A A T G C A T G C T A G C T G A C A C T G T A C G A C T G A T C G T A C G T C A G C T G A A T C G T G A C C T A G A T G C T A G C A T G C G T C A A G T C A T G C A T G C A T G C G C A T T A C G A C T G T A C G | 1e-17 | -4.123e+01 | 1.11% | 0.02% | 43.1bp (0.0bp) | PB0101.1\_Zic1\_1/Jaspar(0.463) More Information | Similar Motifs Found | motif file (matrix) |
| 10 | A C T G A G T C A C G T A C T G A C T G A C G T A T C G A G C T A G T C A C G T C G T A A C T G | 1e-17 | -3.926e+01 | 1.28% | 0.06% | 43.7bp (50.1bp) | Smad4/MA1153.1/Jaspar(0.639) More Information | Similar Motifs Found | motif file (matrix) |
| 11 | C G T A A C G T C T G A A C G T C G T A C G T A A C G T C G T A G T A C C G T A C T G A A C G T | 1e-16 | -3.724e+01 | 1.02% | 0.04% | 59.4bp (19.7bp) | PH0075.1\_Hoxd10/Jaspar(0.654) More Information | Similar Motifs Found | motif file (matrix) |
| 12 | C G T A T G C A C G T A C A T G G A C T A C G T C G T A T A C G T G C A G A C T C G A T G T A C | 1e-15 | -3.581e+01 | 1.19% | 0.05% | 54.0bp (20.2bp) | PHOX2B/MA0681.2/Jaspar(0.561) More Information | Similar Motifs Found | motif file (matrix) |
| 13 | A C T G A T G C A C G T A C T G T C A G A T G C A G T C C G T A A C T G A T C G C G T A C A G T | 1e-15 | -3.581e+01 | 1.19% | 0.06% | 44.7bp (49.8bp) | Bcl11a(Zf)/HSPC-BCL11A-ChIP-Seq(GSE104676)/Homer(0.709) More Information | Similar Motifs Found | motif file (matrix) |
| 14 | C G A T G C T A C G T A C T G A C T A G C G A T C T G A C T A G C G T A C G T A C T A G C G T A T A G C C G T A A T G C | 1e-15 | -3.500e+01 | 1.45% | 0.10% | 50.9bp (60.7bp) | MYNN(Zf)/HEK293-MYNN.eGFP-ChIP-Seq(Encode)/Homer(0.568) More Information | Similar Motifs Found | motif file (matrix) |
| 15 | C A G T A C T G C A T G A T G C G T C A C G T A C G T A C G A T G T C A T C G A | 1e-15 | -3.488e+01 | 2.55% | 0.37% | 60.6bp (51.8bp) | NFAT5/MA0606.1/Jaspar(0.694) More Information | Similar Motifs Found | motif file (matrix) |
| 16 | C A G T T C A G G T C A C G T A C A G T C G A T G T C A G C T A G T A C G A C T C A T G C G T A | 1e-14 | -3.415e+01 | 1.53% | 0.11% | 57.1bp (50.2bp) | PB0081.1\_Tcf1\_1/Jaspar(0.680) More Information | Similar Motifs Found | motif file (matrix) |
| 17 | C T G A A C T G T C G A G T C A C G T A C T A G T C G A G C A T G A C T G A C T T G A C G C T A | 1e-14 | -3.333e+01 | 0.94% | 0.04% | 43.8bp (59.3bp) | PB0126.1\_Gata5\_2/Jaspar(0.638) More Information | Similar Motifs Found | motif file (matrix) |
| 18 | A C G T C G T A C T A G C G T A C T G A A T C G C G A T G T C A C G T A C G T A C G A T C G T A | 1e-14 | -3.333e+01 | 0.94% | 0.00% | 39.5bp (0.0bp) | PB0015.1\_Foxa2\_1/Jaspar(0.725) More Information | Similar Motifs Found | motif file (matrix) |
| 19 | C G A T G A T C G T A C G T C A C G T A A G T C G A T C G A T C C G T A G A T C A T G C G A T C G T A C C G T A A T C G | 1e-14 | -3.333e+01 | 0.94% | 0.00% | 53.3bp (0.0bp) | KLF4/MA0039.4/Jaspar(0.568) More Information | Similar Motifs Found | motif file (matrix) |
| 20 | T A G C A T C G A T G C T A C G A T G C A C T G A T C G T A C G A G T C A G T C G T A C C G A T A T C G A T C G A G T C A T C G A G C T A T C G A T G C A C T G | 1e-14 | -3.333e+01 | 0.94% | 0.04% | 50.2bp (0.5bp) | POL006.1\_BREu/Jaspar(0.597) More Information | Similar Motifs Found | motif file (matrix) |
| 21 | A T G C C G T A C T A G T G A C C G T A T C G A G C T A A G T C C G A T C G T A | 1e-14 | -3.318e+01 | 5.87% | 1.97% | 55.0bp (59.4bp) | ZNF317/MA1593.1/Jaspar(0.701) More Information | Similar Motifs Found | motif file (matrix) |
| 22 | C G A T A T C G A G T C A G T C C G T A A G T C G C A T A C T G G T C A A G T C A G T C A C G T | 1e-14 | -3.244e+01 | 1.11% | 0.06% | 56.3bp (59.6bp) | FXR(NR),IR1/Liver-FXR-ChIP-Seq(Chong\_et\_al.)/Homer(0.712) More Information | Similar Motifs Found | motif file (matrix) |
| 23 | A G T C C A G T A C T G C G T A C G T A C G T A C G A T A C G T A C G T A C T G | 1e-13 | -3.117e+01 | 1.79% | 0.20% | 39.8bp (36.9bp) | PB0169.1\_Sox15\_2/Jaspar(0.684) More Information | Similar Motifs Found | motif file (matrix) |
| 24 | T A G C G A C T G C T A C A T G T G A C G C T A C G T A A G T C C G T A C T A G T C A G C G T A T C G A A C G T C A G T | 1e-13 | -3.098e+01 | 1.62% | 0.16% | 52.7bp (40.5bp) | IKZF1/MA1508.1/Jaspar(0.733) More Information | Similar Motifs Found | motif file (matrix) |
| 25 | A G T C C G T A A C G T C G T A C G T A C A G T G C T A A G T C C G T A A C G T C T A G C A T G | 1e-13 | -3.036e+01 | 1.19% | 0.08% | 55.0bp (29.4bp) | OCT:OCT-short(POU,Homeobox)/NPC-OCT6-ChIP-Seq(GSE43916)/Homer(0.656) More Information | Similar Motifs Found | motif file (matrix) |
| 26 | G C T A C G T A C A T G G A T C G A T C G A T C G C A T A C G T G A C T C A G T A C T G C G A T G C T A C G T A C T A G | 1e-13 | -3.036e+01 | 1.19% | 0.07% | 61.6bp (38.5bp) | ZFP42/MA1651.1/Jaspar(0.589) More Information | Similar Motifs Found | motif file (matrix) |
| 27 | C T G A G T A C G T C A G C A T G C T A C G A T C G T A C A T G A G T C G A C T C T G A G T C A | 1e-12 | -2.954e+01 | 1.87% | 0.24% | 51.1bp (53.9bp) | NEUROG2/MA0669.1/Jaspar(0.576) More Information | Similar Motifs Found | motif file (matrix) |
| 28 | A C T G G T A C C G T A A C T G A C T G A C G T C T A G T G A C C G T A A C G T | 1e-12 | -2.951e+01 | 0.85% | 0.04% | 53.9bp (5.9bp) | Snail1(Zf)/LS174T-SNAIL1.HA-ChIP-Seq(GSE127183)/Homer(0.784) More Information | Similar Motifs Found | motif file (matrix) |
| 29 | A C G T C T A G G T A C A C G T A C G T A C G T A G T C A C G T A C G T A C T G G T A C G T A C | 1e-12 | -2.951e+01 | 0.85% | 0.00% | 61.2bp (0.0bp) | Hoxc9(Homeobox)/Ainv15-Hoxc9-ChIP-Seq(GSE21812)/Homer(0.692) More Information | Similar Motifs Found | motif file (matrix) |
| 30 | A G C T G T A C G T A C A G T C G A C T A G T C T A C G T C A G C A T G A T C G C A G T A T C G G T A C T A C G A G T C A G T C G A T C G C A T C A G T A G T C | 1e-12 | -2.951e+01 | 0.85% | 0.03% | 53.3bp (15.2bp) | Ebf2/MA1604.1/Jaspar(0.564) More Information | Similar Motifs Found | motif file (matrix) |
| 31 | A T C G A C G T C A T G T A C G C T G A T A C G C G T A A T G C C T G A G T C A C A T G A T C G C T G A A C T G A C T G A T C G C T G A T G A C T C G A G T A C | 1e-12 | -2.951e+01 | 0.85% | 0.03% | 54.8bp (10.6bp) | Bcl11a(Zf)/HSPC-BCL11A-ChIP-Seq(GSE104676)/Homer(0.484) More Information | Similar Motifs Found | motif file (matrix) |
| 32 | G T A C A T G C T A G C G T C A T C A G T A G C A T G C A T C G C A G T A C T G T A C G T C G A G T A C T C G A T A G C T G C A A T G C C T G A A T G C A C T G C A T G T C A G A T C G G T A C G A T C T G C A C A G T C A T G T G A C G T A C | 1e-12 | -2.951e+01 | 0.85% | 0.03% | 49.2bp (6.8bp) | PB0153.1\_Nr2f2\_2/Jaspar(0.453) More Information | Similar Motifs Found | motif file (matrix) |
| 33 | A T G C G T C A A G T C G A C T A T C G G T C A A G T C G A T C G A C T G A C T T A C G T C G A A C T G G T A C A T C G C G T A C A T G A G T C A G T C T C G A G A C T C G A T A T C G G C T A T G A C T G A C C A G T C A G T A C T G G T C A | 1e-12 | -2.951e+01 | 0.85% | 0.00% | 48.3bp (0.0bp) | ESRRA/MA0592.3/Jaspar(0.554) More Information | Similar Motifs Found | motif file (matrix) |
| 34 | G A C T C T G A G C T A G A T C A C T G C T A G C A G T C T A G T C G A T C G A C T G A A C G T A G T C G T A C G C T A T G A C G A C T C T G A C T G A G C T A G C T A C G T A C T A G G T A C G A C T C G T A T C G A A C T G A T G C C T G A A G T C T G C A C G T A T C G A C A T G T C A G C G T A T A C G G A T C G C T A | 1e-12 | -2.951e+01 | 0.85% | 0.00% | 57.0bp (0.0bp) | PH0037.1\_Hdx/Jaspar(0.386) More Information | Similar Motifs Found | motif file (matrix) |
| 35 | A C G T A G C T A C G T A C T G A G T C A T C G A G T C C T A G T C A G A C T G | 1e-12 | -2.914e+01 | 1.02% | 0.05% | 59.9bp (22.6bp) | TCFL5/MA0632.2/Jaspar(0.715) More Information | Similar Motifs Found | motif file (matrix) |
| 36 | G T C A C T G A A G T C G C A T A G T C G T A C G T C A G T C A T G A C A G T C G A T C T A G C | 1e-12 | -2.914e+01 | 1.02% | 0.05% | 51.9bp (61.5bp) | GFY(?)/Promoter/Homer(0.591) More Information | Similar Motifs Found | motif file (matrix) |
| 37 | A C G T T A G C A C T G A C T G A C T G A G T C C G A T G A C T G T A C A G C T | 1e-12 | -2.785e+01 | 1.87% | 0.27% | 62.1bp (40.7bp) | OSR2/MA1646.1/Jaspar(0.725) More Information | Similar Motifs Found | motif file (matrix) |
| 38 \* | T G A C G C T A G A T C T A C G G A C T T A C G C T A G A G C T G T A C A G T C | 1e-11 | -2.739e+01 | 1.11% | 0.08% | 48.9bp (52.9bp) | MAX::MYC/MA0059.1/Jaspar(0.797) More Information | Similar Motifs Found | motif file (matrix) |
| 39 \* | C T A G G T A C A G T C A G T C A G T C C G A T A G T C A G C T A T G C C G T A A C T G A G T C | 1e-11 | -2.739e+01 | 1.11% | 0.08% | 54.0bp (33.6bp) | PB0114.1\_Egr1\_2/Jaspar(0.643) More Information | Similar Motifs Found | motif file (matrix) |
| 40 \* | A G T C A T C G C G A T C A G T A C G T A G C T A G C T A C G T A G T C A G T C | 1e-11 | -2.667e+01 | 2.13% | 0.35% | 51.5bp (62.9bp) | NFATC2/MA0152.1/Jaspar(0.773) More Information | Similar Motifs Found | motif file (matrix) |
| 41 \* | A C G T C G T A C T G A G T C A C A G T C T A G C G A T G T C A G C T A A C G T A T C G C G T A C T G A C G T A C G A T | 1e-11 | -2.619e+01 | 1.28% | 0.12% | 53.7bp (53.9bp) | Hoxd12(Homeobox)/ChickenMSG-Hoxd12.Flag-ChIP-Seq(GSE86088)/Homer(0.754) More Information | Similar Motifs Found | motif file (matrix) |
| 42 \* | A C G T A C G T A C G T C G T A T C G A C G T A A T C G A C T G C G T A A C T G | 1e-11 | -2.592e+01 | 0.94% | 0.05% | 40.5bp (14.3bp) | LIN54/MA0619.1/Jaspar(0.641) More Information | Similar Motifs Found | motif file (matrix) |
| 43 \* | C A G T C A G T A C T G G T C A G T C A G C T A C T G A C G A T C T A G G C T A G C T A C A G T A G T C C G T A G A C T | 1e-11 | -2.592e+01 | 0.94% | 0.05% | 54.8bp (17.7bp) | DUX4/MA0468.1/Jaspar(0.629) More Information | Similar Motifs Found | motif file (matrix) |
| 44 \* | C T A G C T A G A G T C A G C T A C G T A T G C G A C T C A T G A T C G G T C A G A T C A G T C G A T C C G T A A C T G | 1e-11 | -2.592e+01 | 0.94% | 0.05% | 54.6bp (40.2bp) | OSR2/MA1646.1/Jaspar(0.693) More Information | Similar Motifs Found | motif file (matrix) |
| 45 \* | A G T C G C T A T C A G C G T A G A C T G T C A C T G A T G A C G C T A G C T A C G T A T G C A G A T C G T A C C T G A T A G C G T C A A C T G C T G A T C G A C G T A T G C A G T C A C G T A G T C A C T G A G A T C G T A C G A T C G T C A | 1e-11 | -2.592e+01 | 0.94% | 0.06% | 52.7bp (6.5bp) | RUNX1/MA0002.2/Jaspar(0.497) More Information | Similar Motifs Found | motif file (matrix) |
| 46 \* | A G C T G A C T A G T C A C T G A C G T C G T A C G T A A C T G A T G C A G T C | 1e-11 | -2.578e+01 | 0.77% | 0.00% | 68.6bp (0.0bp) | Pitx1(Homeobox)/Chicken-Pitx1-ChIP-Seq(GSE38910)/Homer(0.663) More Information | Similar Motifs Found | motif file (matrix) |
| 47 \* | G A T C G T C A C T A G T A G C G A C T A G T C G C T A A T G C G C T A A G T C G T A C G A C T | 1e-11 | -2.578e+01 | 0.77% | 0.03% | 46.1bp (11.8bp) | TBX15/MA0803.1/Jaspar(0.780) More Information | Similar Motifs Found | motif file (matrix) |
| 48 \* | C A T G A G T C A C T G T A C G A C T G A C T G T G C A A G T C A C T G G T A C G T A C T C G A | 1e-11 | -2.578e+01 | 0.77% | 0.04% | 66.7bp (33.3bp) | HINFP/MA0131.2/Jaspar(0.636) More Information | Similar Motifs Found | motif file (matrix) |
| 49 \* | A C T G A G T C A C G T A C G T A C G T A C G T A G T C A C G T A C T G A C G T A C T G A C G T | 1e-11 | -2.578e+01 | 0.77% | 0.04% | 41.5bp (9.1bp) | Stat2/MA1623.1/Jaspar(0.679) More Information | Similar Motifs Found | motif file (matrix) |
| 50 \* | A G T C A G C T A T G C C G T A C A T G T A G C G C T A A G T C A G C T A G T C A G T C G T C A | 1e-11 | -2.578e+01 | 0.77% | 0.02% | 59.9bp (0.0bp) | Srebp1a(bHLH)/HepG2-Srebp1a-ChIP-Seq(GSE31477)/Homer(0.608) More Information | Similar Motifs Found | motif file (matrix) |
| 51 \* | A G T C T G A C T A G C G T C A A T C G T A G C T A G C A G C T A G T C A G T C T A G C C T G A A T G C G A T C G A C T G A T C G T C A A G T C T G C A A G T C G T A C A G C T A T G C A G T C C A G T A G T C A G T C A G T C G C A T T A C G G T A C A G T C G C T A A T G C A G T C | 1e-11 | -2.578e+01 | 0.77% | 0.03% | 47.6bp (0.0bp) | TBX18/MA1565.1/Jaspar(0.507) More Information | Similar Motifs Found | motif file (matrix) |
| 52 \* | T G A C C A T G G A T C A C T G T A G C A G C T A G C T G C T A T A G C A T G C | 1e-10 | -2.385e+01 | 1.36% | 0.15% | 59.2bp (66.4bp) | SD0001.1\_at\_AC\_acceptor/Jaspar(0.718) More Information | Similar Motifs Found | motif file (matrix) |
| 53 \* | C T G A A C T G A T C G A G T C A G C T G A C T C G A T A C T G G C T A A T C G C T A G G C T A A T C G T A C G T G A C | 1e-10 | -2.385e+01 | 1.36% | 0.15% | 63.3bp (30.7bp) | TCF7/MA0769.2/Jaspar(0.627) More Information | Similar Motifs Found | motif file (matrix) |
| 54 \* | A C G T C A G T A G T C G A C T A G T C G T A C G C T A A G T C G C A T T C A G G A T C G A C T T G A C C G T A A C T G | 1e-10 | -2.368e+01 | 1.28% | 0.14% | 46.1bp (53.5bp) | ZNF354C/MA0130.1/Jaspar(0.584) More Information | Similar Motifs Found | motif file (matrix) |
| 55 \* | A C G T C T A G A C G T G T A C A C G T C T G A C A G T C G T A A C G T G T A C | 1e-10 | -2.366e+01 | 1.19% | 0.12% | 43.5bp (62.2bp) | PB0163.1\_Six6\_2/Jaspar(0.745) More Information | Similar Motifs Found | motif file (matrix) |
| 56 \* | A C T G A C G T A C T G A C T G A G T C G T A C A C G T G A C T C T A G A G T C | 1e-9 | -2.279e+01 | 0.85% | 0.05% | 54.7bp (19.1bp) | ZNF682/MA1599.1/Jaspar(0.728) More Information | Similar Motifs Found | motif file (matrix) |
| 57 \* | G T A C G A T C G C A T C G T A C G T A C T A G T C A G C G T A T C A G C T G A C T G A C G A T | 1e-9 | -2.279e+01 | 0.85% | 0.06% | 56.0bp (29.7bp) | PB0139.1\_Irf5\_2/Jaspar(0.601) More Information | Similar Motifs Found | motif file (matrix) |
| 58 \* | G C A T A C G T G C T A G C T A G C A T C G T A G C A T C G T A T G C A G C A T G C A T G T C A A C G T G C T A C G A T C G T A C G T A C G A T C G A T G T A C C G T A C G T A A G C T A C G T A G T C C G A T A C G T G C A T G A C T C G A T G C T A A G C T C A T G G C A T G C A T C G A T A C T G G A C T G C T A C T G A G C A T C G A T C A G T C A G T C G T A G A C T T G A C T G C A A G C T C G T A | 1e-9 | -2.279e+01 | 0.85% | 0.05% | 36.4bp (19.1bp) | Pax7(Paired,Homeobox),longest/Myoblast-Pax7-ChIP-Seq(GSE25064)/Homer(0.427) More Information | Similar Motifs Found | motif file (matrix) |
| 59 \* | A C G T A C G T A T C G A G T C C G T A C G T A G T A C A T C G G T A C C G T A | 1e-9 | -2.216e+01 | 0.68% | 0.02% | 36.3bp (0.0bp) | CEBP:AP1(bZIP)/ThioMac-CEBPb-ChIP-Seq(GSE21512)/Homer(0.730) More Information | Similar Motifs Found | motif file (matrix) |
| 60 \* | A C G T T C G A A T C G C T A G T C G A C T G A A C T G T A G C C T A G C T A G A G T C T C A G T G C A A C T G A C T G | 1e-9 | -2.131e+01 | 1.53% | 0.23% | 61.9bp (46.5bp) | ETV4/MA0764.2/Jaspar(0.660) More Information | Similar Motifs Found | motif file (matrix) |
| 61 \* | C T G A C T A G A C T G T G A C C G T A C G T A A C G T A C T G | 1e-9 | -2.120e+01 | 6.80% | 3.21% | 55.9bp (59.0bp) | EWS:ERG-fusion(ETS)/CADO\_ES1-EWS:ERG-ChIP-Seq(SRA014231)/Homer(0.750) More Information | Similar Motifs Found | motif file (matrix) |
| 62 \* | C T A G C T G A A C T G A C G T C T A G A C T G A G C T A C G T | 1e-8 | -2.047e+01 | 8.16% | 4.23% | 57.5bp (54.4bp) | Bapx1(Homeobox)/VertebralCol-Bapx1-ChIP-Seq(GSE36672)/Homer(0.801) More Information | Similar Motifs Found | motif file (matrix) |
| 63 \* | A G T C G T C A T A C G A C G T A C T G A G T C C G T A A C T G C A T G A T G C A C G T A T C G C T A G A G T C A G C T | 1e-8 | -1.865e+01 | 0.60% | 0.00% | 29.6bp (0.0bp) | ZNF143/MA0088.2/Jaspar(0.571) More Information | Similar Motifs Found | motif file (matrix) |
| 64 \* | C G T A G T C A G T A C A T C G C T A G A C T G | 1e-7 | -1.818e+01 | 47.62% | 39.56% | 57.4bp (56.5bp) | PB0139.1\_Irf5\_2/Jaspar(0.688) More Information | Similar Motifs Found | motif file (matrix) |
| 65 \* | A C T G A G T C A C G T C G T A A C G T A C G T A G C T A C G T A C G T A C G T | 1e-7 | -1.683e+01 | 0.68% | 0.06% | 46.2bp (48.0bp) | Mef2c(MADS)/GM12878-Mef2c-ChIP-Seq(GSE32465)/Homer(0.793) More Information | Similar Motifs Found | motif file (matrix) |
| 66 \* | A C G T A C T G A T G C T A C G C G T A C T G A A C G T A G T C A G T C C G A T | 1e-7 | -1.683e+01 | 0.68% | 0.04% | 43.6bp (46.8bp) | Oct11(POU,Homeobox)/NCIH1048-POU2F3-ChIP-seq(GSE115123)/Homer(0.673) More Information | Similar Motifs Found | motif file (matrix) |
| 67 \* | A C G T A C G T A C G T A G T C C G T A C G T A C G T A A C G T A C G T A G T C A C G T A C G T A C T G C G T A C G T A | 1e-7 | -1.683e+01 | 0.68% | 0.04% | 43.5bp (1.0bp) | PB0197.1\_Zfp105\_2/Jaspar(0.640) More Information | Similar Motifs Found | motif file (matrix) |
| 68 \* | C G T A A T C G A C G T A G T C A C G T A C G T A G C T A C T G | 1e-6 | -1.560e+01 | 2.89% | 1.04% | 59.7bp (57.5bp) | SOX10/MA0442.2/Jaspar(0.734) More Information | Similar Motifs Found | motif file (matrix) |
| 69 \* | A T C G T A G C G T C A T A C G T G C A T G A C G T A C G A T C A G T C T G A C C G T A A T C G G A T C G A C T A T G C | 1e-6 | -1.529e+01 | 0.51% | 0.01% | 49.8bp (0.0bp) | ZIC5/MA1584.1/Jaspar(0.616) More Information | Similar Motifs Found | motif file (matrix) |
| 70 \* | C G T A C G T A A G T C A C G T A G T C A C T G C G T A A C T G | 1e-6 | -1.529e+01 | 0.51% | 0.03% | 47.7bp (37.5bp) | Nkx2.1(Homeobox)/LungAC-Nkx2.1-ChIP-Seq(GSE43252)/Homer(0.629) More Information | Similar Motifs Found | motif file (matrix) |
| 71 \* | T C G A T C A G C T A G A T C G A G T C A C T G A T G C C T A G | 1e-6 | -1.472e+01 | 5.95% | 3.14% | 54.2bp (60.9bp) | PB0009.1\_E2F3\_1/Jaspar(0.847) More Information | Similar Motifs Found | motif file (matrix) |
| 72 \* | C G T A A C G T A C T G C G T A A G T C C G T A C G T A C G T A A C T G A C T G A C G T C G T A C G T A C G T A C G T A | 1e-6 | -1.401e+01 | 0.60% | 0.04% | 47.0bp (54.1bp) | Sox3(HMG)/NPC-Sox3-ChIP-Seq(GSE33059)/Homer(0.640) More Information | Similar Motifs Found | motif file (matrix) |
| 73 \* | A C G T A C G T C G T A A G T C C T G A A C T G A C G T A G T C | 1e-5 | -1.350e+01 | 1.96% | 0.61% | 56.3bp (48.7bp) | ZBTB32/MA1580.1/Jaspar(0.685) More Information | Similar Motifs Found | motif file (matrix) |
| 74 \* | A C T G A G T C A C G T G T A C A C T G C G T A A C G T G T C A | 1e-5 | -1.327e+01 | 1.53% | 0.40% | 44.5bp (44.0bp) | HNF6(Homeobox)/Liver-Hnf6-ChIP-Seq(ERP000394)/Homer(0.682) More Information | Similar Motifs Found | motif file (matrix) |
| 75 \* | A G T C C G T A A G T C A T G C A C G T C G T A A G T C C G T A | 1e-5 | -1.229e+01 | 1.87% | 0.62% | 64.6bp (47.3bp) | TCF3/MA0522.3/Jaspar(0.735) More Information | Similar Motifs Found | motif file (matrix) |
| 76 \* | A C G T A C T G A C T G C G T A C G T A C G T A A G T C A G T C C G T A A C G T | 1e-4 | -1.133e+01 | 0.51% | 0.06% | 45.9bp (97.5bp) | NFATC4/MA1525.1/Jaspar(0.703) More Information | Similar Motifs Found | motif file (matrix) |
| 77 \* | C G T A C G T A A C G T A G T C A G T C A C T G A C T G A C T G | 1e-4 | -1.133e+01 | 0.51% | 0.06% | 41.9bp (44.3bp) | PB0185.1\_Tcf1\_2/Jaspar(0.769) More Information | Similar Motifs Found | motif file (matrix) |
| 78 \* | A C T G A C G T A G C T A G T C A C G T A C T G A G T C A C G T | 1e-4 | -1.040e+01 | 2.89% | 1.34% | 53.4bp (59.3bp) | ZNF317/MA1593.1/Jaspar(0.680) More Information | Similar Motifs Found | motif file (matrix) |
| 79 \* | A C G T C G T A A C T G A G T C A C T G C G T A | 1e-4 | -9.680e+00 | 4.85% | 2.80% | 64.5bp (57.6bp) | POL010.1\_DCE\_S\_III/Jaspar(0.705) More Information | Similar Motifs Found | motif file (matrix) |
| 80 \* | A C G T A C G T A C G T A G T C A G T C A G T C A C T G A G T C | 1e-4 | -9.580e+00 | 0.60% | 0.09% | 75.8bp (43.2bp) | E2F7(E2F)/Hela-E2F7-ChIP-Seq(GSE32673)/Homer(0.937) More Information | Similar Motifs Found | motif file (matrix) |
| 81 \* | G T C A A T G C T A G C A T G C G A T C A G T C G T C A A T G C G T A C A G T C G A T C C T G A G T A C G T C A A G T C T A G C A G T C G T C A A T G C A C G T G A T C A G T C A T G C A G C T A T C G G T C A A T G C G T A C A G T C G T C A A G T C G T A C A T G C G T A C A T C G A T G C A C G T G T A C C G T A A G T C C G T A A G T C G T A C A G C T G T A C A G T C A G C T A G T C A G T C A T G C | 1e-3 | -9.034e+00 | 0.34% | 0.00% | 40.9bp (0.0bp) | ZKSCAN5/MA1652.1/Jaspar(0.394) More Information | Similar Motifs Found | motif file (matrix) |
| 82 \* | A G T C A T G C A C T G A G T C A G C T A C G T | 1e-3 | -7.852e+00 | 16.92% | 13.45% | 57.5bp (58.8bp) | CENPB/MA0637.1/Jaspar(0.660) More Information | Similar Motifs Found | motif file (matrix) |
| 83 \* | T C G A T C A G T A G C A T G C G A T C A G C T A G C T G T A C A G T C A T G C G A T C G A T C A T C G C T A G A T C G A C T G A C G T C T A G T C G A T C A G A T G C T A G C G A T C A G C T A G C T G T A C A G T C A T G C G A T C G A T C A T C G C T A G A T C G A C T G A C G T | 1e-2 | -6.472e+00 | 1.11% | 0.43% | 47.4bp (40.7bp) | PB0085.1\_Tcfap2a\_1/Jaspar(0.488) More Information | Similar Motifs Found | motif file (matrix) |
| 84 \* | T A C G A T G C T A G C T A G C T A C G A T C G T A G C A T G C A T C G A T G C A T C G A T C G A T G C A T G C A T G C A T C G T A C G T A C G A T C G T A G C T A C G T A G C A T C G T A G C A T C G T A G C T A G C A T C G T A C G A T C G A T G C A T G C A T G C A T C G A T C G A T G C A T G C A T G C T A G C A T G C A T C G T A G C T A G C A T G C T A C G | 1e-1 | -3.683e+00 | 0.17% | 0.00% | 12.4bp (0.0bp) | Sp1(Zf)/Promoter/Homer(0.333) More Information | Similar Motifs Found | motif file (matrix) |
