# Supplementary material for: Global hypo-methylation in a proportion of glioblastoma enriched for an astrocytic signature is associated with increased invasion and altered immune landscape
Source: eLife. 2022 Nov 22;11:e77335. doi: 10.7554/eLife.77335 (PMC9681209; doi:10.7554/eLife.77335)
Supplement: Figure 2—source data 1. [file elife-77335-fig2-data1.zip › Figure_2_source_data_1/Figure_2C/knownResults.html]

/data/Blizard-MarinoLab/Nicola\_Pomella/Motifs\_James/201117\_2/ - Homer Known Motif Enrichment Results


# Homer Known Motif Enrichment Results (/data/Blizard-MarinoLab/Nicola\_Pomella/Motifs\_James/201117\_2/)

Homer *de novo* Motif Results  
Gene Ontology Enrichment Results  
Known Motif Enrichment Results (txt file)  
Total Target Sequences = 1176, Total Background Sequences = 4844

|  |  |  |  |  |  |  |  |  |  |  |  |
| --- | --- | --- | --- | --- | --- | --- | --- | --- | --- | --- | --- |
| Rank | Motif | Name | P-value | log P-pvalue | q-value (Benjamini) | # Target Sequences with Motif | % of Targets Sequences with Motif | # Background Sequences with Motif | % of Background Sequences with Motif | Motif File | SVG |
| 1 | G A C T C T A G G A T C C A G T A C T G C T G A A T G C G C A T A T G C C T G A | MafA(bZIP)/Islet-MafA-ChIP-Seq(GSE30298)/Homer | 1e-3 | -8.268e+00 | 0.1129 | 93.0 | 7.91% | 263.5 | 5.44% | motif file (matrix) | svg |
| 2 | T C G A T C G A T A C G G A T C G T C A G T A C C G A T A G C T G T C A T G C A | Nkx3.1(Homeobox)/LNCaP-Nkx3.1-ChIP-Seq(GSE28264)/Homer | 1e-2 | -5.947e+00 | 0.5750 | 266.0 | 22.62% | 934.3 | 19.31% | motif file (matrix) | svg |
| 3 | C G T A A C G T A C T G G T A C C G T A A C G T C G T A C G T A A C G T A C T G A G T C C G T A A C G T C T G A G C A T | OCT:OCT-short(POU,Homeobox)/NPC-OCT6-ChIP-Seq(GSE43916)/Homer | 1e-2 | -5.688e+00 | 0.5750 | 105.0 | 8.93% | 330.4 | 6.83% | motif file (matrix) | svg |
| 4 | C A G T T G C A A C G T A C T G C G T A A T C G C G A T T G A C C G T A A C G T | BATF(bZIP)/Th17-BATF-ChIP-Seq(GSE39756)/Homer | 1e-2 | -5.654e+00 | 0.5750 | 75.0 | 6.38% | 223.3 | 4.61% | motif file (matrix) | svg |
| 5 | C T G A C T G A T A G C G A T C G C T A G T A C A C G T G A T C T G C A C G T A | Nkx2.5(Homeobox)/HL1-Nkx2.5.biotin-ChIP-Seq(GSE21529)/Homer | 1e-2 | -5.452e+00 | 0.5750 | 241.0 | 20.49% | 846.2 | 17.49% | motif file (matrix) | svg |
| 6 | T A C G T A C G G T A C A T C G T A C G T A C G G T C A C T G A C G T A G A C T | E2F4(E2F)/K562-E2F4-ChIP-Seq(GSE31477)/Homer | 1e-2 | -5.278e+00 | 0.5750 | 40.0 | 3.40% | 106.2 | 2.20% | motif file (matrix) | svg |
| 7 | C A G T T C A G G A T C A C T G A C G T C T A G A C T G A C T G G A C T C T A G | Egr1(Zf)/K562-Egr1-ChIP-Seq(GSE32465)/Homer | 1e-2 | -5.227e+00 | 0.5750 | 73.0 | 6.21% | 220.3 | 4.55% | motif file (matrix) | svg |
| 8 | T A C G T C G A G A C T A C T G C T G A A G T C T C A G G A C T T G A C C T G A | Atf1(bZIP)/K562-ATF1-ChIP-Seq(GSE31477)/Homer | 1e-2 | -5.004e+00 | 0.5750 | 69.0 | 5.87% | 208.2 | 4.30% | motif file (matrix) | svg |
| 9 | G T A C G C T A T C A G C T G A C T A G C A T G A G C T G A T C T G C A T C G A C T G A A C T G C A G T A G T C G A T C G C T A | HNF4a(NR),DR1/HepG2-HNF4a-ChIP-Seq(GSE25021)/Homer | 1e-2 | -4.914e+00 | 0.5750 | 49.0 | 4.17% | 139.5 | 2.88% | motif file (matrix) | svg |
| 10 | A G C T T C G A G T A C T C G A A T G C A T G C G C A T A T C G A G T C A G C T | Snail1(Zf)/LS174T-SNAIL1.HA-ChIP-Seq(GSE127183)/Homer | 1e-2 | -4.743e+00 | 0.5750 | 91.0 | 7.74% | 290.2 | 6.00% | motif file (matrix) | svg |
