# Supplementary material for: Global hypo-methylation in a proportion of glioblastoma enriched for an astrocytic signature is associated with increased invasion and altered immune landscape
Source: eLife. 2022 Nov 22;11:e77335. doi: 10.7554/eLife.77335 (PMC9681209; doi:10.7554/eLife.77335)
Supplement: Figure 2—source data 1. [file elife-77335-fig2-data1.zip › Figure_2_source_data_1/Figure_2I_J/homerResults.html]

/data/Blizard-MarinoLab/Nicola\_Pomella/Motifs\_James/201117\_1// - Homer de novo Motif Results


# Homer *de novo* Motif Results (/data/Blizard-MarinoLab/Nicola\_Pomella/Motifs\_James/201117\_1//)

Known Motif Enrichment Results  
Gene Ontology Enrichment Results  
If Homer is having trouble matching a motif to a known motif, try copy/pasting the matrix file into
STAMP  
More information on motif finding results: HOMER
| Description of Results
| Tips
  
Total target sequences = 726  
Total background sequences = 284  
\* - possible false positive  

|  |  |  |  |  |  |  |  |  |
| --- | --- | --- | --- | --- | --- | --- | --- | --- |
| Rank | Motif | P-value | log P-pvalue | % of Targets | % of Background | STD(Bg STD) | Best Match/Details | Motif File |
| 1 | A T G C G A T C G A C T A C G T A T G C G T A C C G A T A T C G A T G C G T A C C G T A A T C G | 1e-59 | -1.367e+02 | 8.26% | 0.36% | 57.2bp (0.0bp) | ETV4/MA0764.2/Jaspar(0.739) More Information | Similar Motifs Found | motif file (matrix) |
| 2 | A G T C C G T A A T C G G T A C G C T A A T G C A C G T A C T G | 1e-57 | -1.335e+02 | 8.13% | 0.57% | 51.9bp (41.3bp) | PB0099.1\_Zfp691\_1/Jaspar(0.696) More Information | Similar Motifs Found | motif file (matrix) |
| 3 | T A C G C T G A C T A G C T G A A C G T A C T G T A C G C T A G T C A G T A G C | 1e-52 | -1.208e+02 | 7.58% | 0.00% | 55.1bp (0.0bp) | KLF4/MA0039.4/Jaspar(0.766) More Information | Similar Motifs Found | motif file (matrix) |
| 4 | A C T G A T C G C A G T T C A G G T C A G T A C G A T C T G A C C G A T A T C G G A T C C G A T A G T C G T A C G A C T | 1e-48 | -1.114e+02 | 7.16% | 0.52% | 58.9bp (0.0bp) | Znf263(Zf)/K562-Znf263-ChIP-Seq(GSE31477)/Homer(0.628) More Information | Similar Motifs Found | motif file (matrix) |
| 5 | A C G T A G T C C G T A A C T G A T C G C T G A A C T G A C T G | 1e-48 | -1.114e+02 | 7.16% | 0.70% | 59.2bp (0.0bp) | Zic1::Zic2/MA1628.1/Jaspar(0.792) More Information | Similar Motifs Found | motif file (matrix) |
| 6 | A G T C G C A T T A C G G C A T A C T G A G T C G A C T G T A C C T G A A C T G G A T C T A G C T A G C G C A T T A C G | 1e-47 | -1.083e+02 | 7.02% | 0.40% | 41.9bp (10.6bp) | ZNF416(Zf)/HEK293-ZNF416.GFP-ChIP-Seq(GSE58341)/Homer(0.622) More Information | Similar Motifs Found | motif file (matrix) |
| 7 | G T A C G A C T C A T G A C T G A C T G C T A G A T C G G A T C G T A C T A G C A T G C C A G T A C T G A C G T A T C G A T G C G C A T A T C G C G A T A T C G G A C T A C T G A T G C A T G C A T G C G C A T A T C G G A C T A C T G C G T A A T G C A G T C G A T C G A T C G A T C A T G C G C A T A T G C G A C T A G C T G A T C A G T C G T A C A C G T C A T G | 1e-47 | -1.083e+02 | 7.02% | 0.69% | 44.2bp (17.8bp) | ZKSCAN5/MA1652.1/Jaspar(0.398) More Information | Similar Motifs Found | motif file (matrix) |
| 8 | T C G A T A G C C T G A T C A G C T G A T C A G T C G A C G T A C T A G C T A G | 1e-44 | -1.026e+02 | 8.54% | 0.75% | 55.5bp (50.3bp) | GATA4/MA0482.2/Jaspar(0.734) More Information | Similar Motifs Found | motif file (matrix) |
| 9 | T A C G C T G A A C T G T C A G A C T G A C T G A T G C T A G C G A T C C A T G C A T G T C A G | 1e-43 | -9.923e+01 | 6.61% | 0.52% | 55.4bp (43.7bp) | PLAGL2/MA1548.1/Jaspar(0.696) More Information | Similar Motifs Found | motif file (matrix) |
| 10 | A T G C C G A T A C T G T A G C G C A T C A T G A C T G A C G T A G T C G C A T | 1e-42 | -9.747e+01 | 8.26% | 1.03% | 47.6bp (13.1bp) | Zic1::Zic2/MA1628.1/Jaspar(0.684) More Information | Similar Motifs Found | motif file (matrix) |
| 11 | G C T A C G T A A C G T G C A T T C G A C G A T G A C T G C A T C A G T C T A G G C T A G C T A G T C A C G T A A C G T | 1e-41 | -9.624e+01 | 6.47% | 0.50% | 54.3bp (18.7bp) | OCT4-SOX2-TCF-NANOG(POU,Homeobox,HMG)/mES-Oct4-ChIP-Seq(GSE11431)/Homer(0.649) More Information | Similar Motifs Found | motif file (matrix) |
| 12 | A G T C A G T C A G C T A G T C C G T A A C T G A G T C A G T C C G T A G A T C A C T G C T A G G T C A A T C G A G T C | 1e-41 | -9.624e+01 | 6.47% | 0.70% | 53.5bp (4.9bp) | TFAP2C(var.2)/MA0814.2/Jaspar(0.581) More Information | Similar Motifs Found | motif file (matrix) |
| 13 | A G T C G T A C G T A C G T A C C G T A A C T G C A T G T C A G A T G C G A C T | 1e-40 | -9.327e+01 | 6.34% | 0.15% | 55.5bp (0.0bp) | EBF1/MA0154.4/Jaspar(0.768) More Information | Similar Motifs Found | motif file (matrix) |
| 14 | T C A G C T A G T C A G T C G A A T C G T G A C G A T C G C A T A T C G T A C G A T G C C G T A T A C G C A T G T C A G C G T A A C T G A T G C C G T A A T C G T C G A C T G A C T A G A C T G T A G C G T C A A G T C A T C G T A C G T G C A | 1e-40 | -9.242e+01 | 7.99% | 0.97% | 49.4bp (38.5bp) | Znf263(Zf)/K562-Znf263-ChIP-Seq(GSE31477)/Homer(0.512) More Information | Similar Motifs Found | motif file (matrix) |
| 15 | C T A G A T C G C G A T G C A T C G T A C T A G A C T G C A G T | 1e-39 | -9.033e+01 | 6.20% | 0.26% | 52.3bp (0.0bp) | SD0001.1\_at\_AC\_acceptor/Jaspar(0.654) More Information | Similar Motifs Found | motif file (matrix) |
| 16 | A T G C G A T C G A C T T A G C A G C T A T C G C G A T T A C G A T C G G A T C G A T C T G A C G C T A A T C G T A G C | 1e-39 | -8.992e+01 | 7.85% | 0.83% | 53.6bp (9.7bp) | PLAG1/MA0163.1/Jaspar(0.590) More Information | Similar Motifs Found | motif file (matrix) |
| 17 | C T A G C T A G A T G C C T A G T A G C C G A T A C T G A T C G A C G T C A T G A T G C G T A C A T G C A G T C T A G C G C T A A T C G C A T G T A C G A T G C G A T C A G T C C A T G A C T G A C T G A T C G T A G C T C A G A T G C T C A G | 1e-39 | -8.992e+01 | 7.85% | 0.83% | 50.3bp (8.5bp) | ZNF416(Zf)/HEK293-ZNF416.GFP-ChIP-Seq(GSE58341)/Homer(0.518) More Information | Similar Motifs Found | motif file (matrix) |
| 18 | C A G T G A C T G A T C A G C T T A G C C G T A T C A G C T G A C T G A A G T C G A T C A G C T | 1e-37 | -8.741e+01 | 6.06% | 0.69% | 54.4bp (35.9bp) | STAT5(Stat)/mCD4+-Stat5-ChIP-Seq(GSE12346)/Homer(0.648) More Information | Similar Motifs Found | motif file (matrix) |
| 19 | C T A G A T C G C T G A T A G C C T G A T A G C G T C A A T G C T A G C G C A T C A T G A T C G T A C G A G T C T A C G A C G T T C A G A C G T T A G C A G T C | 1e-37 | -8.741e+01 | 6.06% | 0.15% | 48.2bp (0.0bp) | ZEB2(Zf)/SNU398-ZEB2-ChIP-Seq(GSE103048)/Homer(0.651) More Information | Similar Motifs Found | motif file (matrix) |
| 20 | C G T A G T C A T G C A C T G A A C G T A C G T C G T A G T A C C G A T C G A T | 1e-34 | -7.879e+01 | 5.65% | 0.47% | 50.8bp (6.7bp) | Dlx1/MA0879.1/Jaspar(0.779) More Information | Similar Motifs Found | motif file (matrix) |
| 21 | C G T A A C T G C G A T C A G T A T C G T C G A A G T C G A T C | 1e-33 | -7.772e+01 | 7.16% | 0.77% | 53.2bp (39.2bp) | NR1I3/MA1534.1/Jaspar(0.729) More Information | Similar Motifs Found | motif file (matrix) |
| 22 | A G T C A G T C C G A T A G C T A T G C A G C T A G T C G T A C G A C T A G T C G A T C G C T A A T C G T G C A T A G C G A C T A T G C T A C G C A G T A C T G A C G T T G C A A G C T G A C T A T C G C G T A A T C G A C G T T A C G A G T C A G C T A G C T C G T A T G A C C G A T T A C G T G C A A T C G A T G C C A T G A T G C A G T C G T C A G A T C A T G C A C G T A T G C G A T C G A C T A G C T | 1e-33 | -7.762e+01 | 8.40% | 1.29% | 45.4bp (7.5bp) | ZSCAN22(Zf)/HEK293-ZSCAN22.GFP-ChIP-Seq(GSE58341)/Homer(0.413) More Information | Similar Motifs Found | motif file (matrix) |
| 23 | A G C T C G A T C G A T A G C T G C A T A G C T C G T A A C T G C G A T C T G A | 1e-32 | -7.596e+01 | 5.51% | 0.62% | 53.8bp (30.4bp) | ZNF384/MA1125.1/Jaspar(0.725) More Information | Similar Motifs Found | motif file (matrix) |
| 24 | C G T A T G A C C G T A A T C G C G A T A T C G C T A G T C A G C G A T A C T G | 1e-32 | -7.570e+01 | 10.33% | 1.90% | 51.0bp (56.5bp) | ZSCAN4/MA1155.1/Jaspar(0.669) More Information | Similar Motifs Found | motif file (matrix) |
| 25 | G A T C G A T C G A T C T C G A G T C A T C G A A G C T A G T C | 1e-32 | -7.534e+01 | 7.02% | 1.00% | 52.5bp (58.7bp) | GCM2/MA0767.1/Jaspar(0.756) More Information | Similar Motifs Found | motif file (matrix) |
| 26 | C A G T C A T G C A T G T G A C G T C A A C G T T A G C C G T A C A T G T A G C G C A T A C T G | 1e-31 | -7.335e+01 | 8.13% | 1.06% | 58.1bp (31.2bp) | Ap4(bHLH)/AML-Tfap4-ChIP-Seq(GSE45738)/Homer(0.729) More Information | Similar Motifs Found | motif file (matrix) |
| 27 | G A C T A G C T G A T C G T A C C G T A T A C G T C G A T A G C G C T A T A G C G T A C G T A C | 1e-31 | -7.335e+01 | 8.13% | 1.26% | 48.8bp (33.1bp) | PB0060.1\_Smad3\_1/Jaspar(0.837) More Information | Similar Motifs Found | motif file (matrix) |
| 28 | T C A G C A T G C T A G C T A G C T A G C T A G T A C G G C T A A C T G C T A G T C A G C T G A C T G A A C T G C T A G | 1e-30 | -7.040e+01 | 5.23% | 0.62% | 57.3bp (6.8bp) | ZNF148/MA1653.1/Jaspar(0.745) More Information | Similar Motifs Found | motif file (matrix) |
| 29 | G A T C C A G T C A T G T A C G T C G A A T C G A T G C T G A C G T A C C A G T A T C G C T A G T A G C G A T C G C A T | 1e-30 | -6.915e+01 | 7.85% | 1.29% | 52.4bp (24.3bp) | Zfx/MA0146.2/Jaspar(0.683) More Information | Similar Motifs Found | motif file (matrix) |
| 30 | T A G C C G T A C T A G A G T C A C G T A T G C G C T A A C T G C T G A C T A G T C A G G T C A C T G A C T A G C G T A | 1e-29 | -6.832e+01 | 6.61% | 0.72% | 58.2bp (13.8bp) | ETV4/MA0764.2/Jaspar(0.629) More Information | Similar Motifs Found | motif file (matrix) |
| 31 | T G A C C G A T A C T G A G C T G A C T C G A T A C G T G A C T G A C T G A T C A G T C C G A T A C T G C G A T C G A T G A T C C G A T A G T C C G A T A C T G G C A T A C G T G A T C A G C T G A C T C G A T C A T G C G A T G A C T G C A T A G C T A G T C G A C T A G T C G C A T A G T C G C A T G A C T G A C T G C A T | 1e-29 | -6.766e+01 | 5.10% | 0.63% | 43.8bp (59.0bp) | PB0148.1\_Mtf1\_2/Jaspar(0.418) More Information | Similar Motifs Found | motif file (matrix) |
| 32 | A C G T A G T C T C A G A G T C G C A T T C G A G T C A G T A C | 1e-29 | -6.766e+01 | 5.10% | 0.53% | 56.9bp (4.2bp) | PRDM14(Zf)/H1-PRDM14-ChIP-Seq(GSE22767)/Homer(0.685) More Information | Similar Motifs Found | motif file (matrix) |
| 33 | A G T C G T A C A C G T T A G C A G C T C G T A G C A T C G T A | 1e-29 | -6.766e+01 | 5.10% | 0.53% | 54.7bp (18.1bp) | POL012.1\_TATA-Box/Jaspar(0.728) More Information | Similar Motifs Found | motif file (matrix) |
| 34 | A T G C C G T A A C T G C A G T C T A G T C G A G T C A G T A C C G T A A T C G C T G A A C T G | 1e-28 | -6.494e+01 | 4.96% | 0.56% | 50.3bp (0.0bp) | ZNF768(Zf)/Rajj-ZNF768-ChIP-Seq(GSE111879)/Homer(0.629) More Information | Similar Motifs Found | motif file (matrix) |
| 35 | A C G T A G C T C A G T C T A G C G A T A G T C G C T A T C G A T C G A C G T A | 1e-26 | -6.151e+01 | 6.20% | 0.84% | 58.1bp (43.1bp) | MEIS1/MA0498.2/Jaspar(0.736) More Information | Similar Motifs Found | motif file (matrix) |
| 36 | C A T G T A C G A G T C A C G T A C G T A G C T A T G C C G T A C A T G T C A G C A G T C A T G | 1e-25 | -5.960e+01 | 4.68% | 0.49% | 47.2bp (0.0bp) | E2A(bHLH),near\_PU.1/Bcell-PU.1-ChIP-Seq(GSE21512)/Homer(0.679) More Information | Similar Motifs Found | motif file (matrix) |
| 37 | A T C G A T G C C G A T A C T G T A G C C A G T A C G T G T A C G T A C T A G C G C A T A C T G T G A C G C T A T G A C | 1e-25 | -5.960e+01 | 4.68% | 0.70% | 55.9bp (0.0bp) | Spz1/MA0111.1/Jaspar(0.650) More Information | Similar Motifs Found | motif file (matrix) |
| 38 | G A C T A G T C G A C T T A C G C T G A T A C G T G C A G A T C G A T C C G T A G T A C A G T C G C T A A G T C G T C A T A C G A G T C G A T C G C A T A T G C | 1e-25 | -5.960e+01 | 4.68% | 0.56% | 52.3bp (14.6bp) | ETS:RUNX(ETS,Runt)/Jurkat-RUNX1-ChIP-Seq(GSE17954)/Homer(0.648) More Information | Similar Motifs Found | motif file (matrix) |
| 39 | C G T A T C A G G A C T G A T C A C T G C A G T C A T G A C G T T G C A C A G T | 1e-24 | -5.697e+01 | 4.55% | 0.70% | 54.9bp (11.7bp) | Npas4(bHLH)/Neuron-Npas4-ChIP-Seq(GSE127793)/Homer(0.689) More Information | Similar Motifs Found | motif file (matrix) |
| 40 | C A G T G C A T A G T C G C T A G T C A G T C A C G T A G C T A A T C G T C G A G T C A G C T A G T C A C G T A G A C T | 1e-23 | -5.490e+01 | 5.79% | 0.81% | 48.4bp (19.8bp) | IRF7/MA0772.1/Jaspar(0.705) More Information | Similar Motifs Found | motif file (matrix) |
| 41 | A C T G A T G C A G C T A G C T T G C A A G T C G A C T A C T G C T G A C T A G G T A C A T C G A G T C A T G C C G T A A T G C G T A C G A C T A G T C G A T C G A C T A C G T T A C G A G C T T A G C A T G C A G C T A T G C G T A C A C T G T A C G G C T A T A C G G A C T A G T C | 1e-23 | -5.438e+01 | 4.41% | 0.60% | 45.9bp (0.0bp) | ZNF135/MA1587.1/Jaspar(0.517) More Information | Similar Motifs Found | motif file (matrix) |
| 42 | A T G C G A T C C G A T C A T G T A C G C T G A A T C G T A G C G A T C G T C A T A G C G T C A | 1e-22 | -5.275e+01 | 5.65% | 0.84% | 54.9bp (55.5bp) | ZBTB6/MA1581.1/Jaspar(0.748) More Information | Similar Motifs Found | motif file (matrix) |
| 43 | G A C T T A G C T A C G T A G C G T C A T G C A A G T C G T A C | 1e-22 | -5.182e+01 | 4.27% | 0.63% | 54.9bp (39.1bp) | PB0029.1\_Hic1\_1/Jaspar(0.736) More Information | Similar Motifs Found | motif file (matrix) |
| 44 | T C A G A T G C G T A C A C T G A C G T T A G C G T C A T C G A G T A C G T C A A T G C G C T A | 1e-21 | -5.062e+01 | 5.51% | 0.91% | 51.6bp (19.6bp) | Tbx20(T-box)/Heart-Tbx20-ChIP-Seq(GSE29636)/Homer(0.695) More Information | Similar Motifs Found | motif file (matrix) |
| 45 | T A C G A C T G T A C G T C A G A T C G T G A C A T C G A C G T A T C G A T C G T A G C T G A C T A G C C T A G T A C G C A T G T A C G T A G C T G A C T A C G T C A G T C A G T A C G A T G C T A G C T G A C T A C G A T C G A T C G T G C A T A C G A G T C T A C G G T A C A T G C T G C A A T G C A T G C A C T G A T C G T A C G T G A C A T G C A G T C A T G C A G T C A T C G T A C G T A G C T A G C | 1e-21 | -4.929e+01 | 4.13% | 0.61% | 51.1bp (29.2bp) | Sp1(Zf)/Promoter/Homer(0.383) More Information | Similar Motifs Found | motif file (matrix) |
| 46 | T C A G T A G C C A T G C T G A A T G C T G C A C G T A A G C T | 1e-21 | -4.929e+01 | 4.13% | 0.70% | 52.0bp (17.6bp) | Sox17/MA0078.1/Jaspar(0.834) More Information | Similar Motifs Found | motif file (matrix) |
| 47 | G A C T G A C T G A T C G A C T A G T C G A C T A G C T A G C T A G T C G A T C G A C T A G T C G A T C G A C T G C T A | 1e-20 | -4.810e+01 | 7.30% | 1.63% | 58.1bp (66.1bp) | Stat2/MA1623.1/Jaspar(0.672) More Information | Similar Motifs Found | motif file (matrix) |
| 48 | A G T C T G C A A G T C C G T A A G T C A C G T A G T C C G T A | 1e-19 | -4.433e+01 | 3.86% | 0.58% | 40.5bp (19.9bp) | MSANTD3/MA1523.1/Jaspar(0.815) More Information | Similar Motifs Found | motif file (matrix) |
| 49 | C T A G A T C G C T G A A T C G T A C G T A G C C T A G T A G C T A G C A G T C C T A G A C T G T C A G A G T C T A G C A T G C T G A C A C G T T A C G A G T C A T C G A T G C A T G C C G A T T A C G A T G C A G T C G A T C A T G C T A G C A T G C T C A G C A T G A T C G T C A G T A C G T A C G T G C A T A G C T A G C T A G C A T C G T A G C T A G C A C T G | 1e-18 | -4.237e+01 | 4.96% | 0.84% | 47.3bp (24.1bp) | NRF1/MA0506.1/Jaspar(0.421) More Information | Similar Motifs Found | motif file (matrix) |
| 50 | A C G T C T A G A C G T A G T C G T A C C G A T A T C G A G T C C G T A T A C G | 1e-17 | -4.038e+01 | 4.82% | 0.88% | 48.1bp (3.6bp) | ERRg(NR)/Kidney-ESRRG-ChIP-Seq(GSE104905)/Homer(0.643) More Information | Similar Motifs Found | motif file (matrix) |
| 51 | C G A T A T C G G A T C C T A G G T A C G C T A A C G T G T C A | 1e-17 | -4.014e+01 | 7.44% | 2.09% | 55.0bp (45.8bp) | PB0104.1\_Zscan4\_1/Jaspar(0.767) More Information | Similar Motifs Found | motif file (matrix) |
| 52 | A C T G A G T C A C T G C G A T A G T C C G A T | 1e-17 | -3.979e+01 | 6.61% | 1.64% | 49.0bp (63.2bp) | Smad4(MAD)/ESC-SMAD4-ChIP-Seq(GSE29422)/Homer(0.732) More Information | Similar Motifs Found | motif file (matrix) |
| 53 | A G T C G C A T A T C G A G T C G A C T A G T C G A C T A G C T A G T C G T A C | 1e-17 | -3.952e+01 | 3.58% | 0.57% | 46.5bp (0.0bp) | ZNF189(Zf)/HEK293-ZNF189.GFP-ChIP-Seq(GSE58341)/Homer(0.751) More Information | Similar Motifs Found | motif file (matrix) |
| 54 | T C G A A T C G T A G C G T A C G T C A A C T G A C T G T G A C G C T A T A C G G T A C G T A C G T A C A G T C C G A T | 1e-16 | -3.717e+01 | 3.44% | 0.26% | 57.7bp (0.0bp) | PB0060.1\_Smad3\_1/Jaspar(0.584) More Information | Similar Motifs Found | motif file (matrix) |
| 55 | A T G C T G C A A T G C T A C G A C G T T C A G A G C T A C T G | 1e-15 | -3.662e+01 | 6.34% | 1.69% | 55.7bp (42.9bp) | MNT(bHLH)/HepG2-MNT-ChIP-Seq(Encode)/Homer(0.883) More Information | Similar Motifs Found | motif file (matrix) |
| 56 | T A G C A G C T A G T C G A T C A T G C A G T C A G T C G T C A A G T C A G T C A G T C G A T C G A T C G A T C T A G C A G T C A G T C G A T C T G A C G A C T A T G C G T C A A T G C T A G C G T A C | 1e-15 | -3.649e+01 | 4.55% | 0.90% | 51.9bp (2.8bp) | PB0097.1\_Zfp281\_1/Jaspar(0.631) More Information | Similar Motifs Found | motif file (matrix) |
| 57 | G A C T A G C T A G T C G C T A G A C T G A T C A G T C G C T A G C A T A G C T G T A C T G C A T G C A A G C T C G A T G A T C A G T C G T A C G T A C C G A T | 1e-15 | -3.486e+01 | 3.31% | 0.49% | 57.6bp (0.0bp) | PB0028.1\_Hbp1\_1/Jaspar(0.576) More Information | Similar Motifs Found | motif file (matrix) |
| 58 | C G A T C G T A G C A T C G T A G C A T C G T A G C A T C G T A G C T A C G T A G C A T C G T A | 1e-13 | -3.037e+01 | 3.03% | 0.50% | 43.2bp (32.7bp) | PB0080.1\_Tbp\_1/Jaspar(0.800) More Information | Similar Motifs Found | motif file (matrix) |
| 59 \* | T G A C C T A G G T C A G C A T T C G A G T C A C G T A T C A G | 1e-11 | -2.703e+01 | 4.68% | 1.13% | 57.9bp (43.5bp) | GATA3/MA0037.3/Jaspar(0.785) More Information | Similar Motifs Found | motif file (matrix) |
| 60 \* | A G T C G T A C G A C T A G C T A G C T A G T C G A T C G C T A C T A G A C T G | 1e-10 | -2.488e+01 | 5.23% | 1.45% | 53.3bp (44.7bp) | Bcl6(Zf)/Liver-Bcl6-ChIP-Seq(GSE31578)/Homer(0.751) More Information | Similar Motifs Found | motif file (matrix) |
| 61 \* | C G A T C G T A G T A C C T G A A C T G A G T C | 1e-10 | -2.443e+01 | 23.83% | 14.71% | 55.4bp (59.2bp) | PH0158.1\_Rhox11\_2/Jaspar(0.842) More Information | Similar Motifs Found | motif file (matrix) |
| 62 \* | A G T C A G T C A G T C A G T C A G T C A G T C A G T C A G T C A G T C A G T C A G T C A G T C A G T C A G T C A G T C A G T C A G T C A G T C A G T C A G T C A G T C A G T C A G T C A G T C A G T C A G T C A G T C A G T C A G T C A G T C A G T C A G T C A G T C A G T C A G T C A G T C A G T C A G T C A G T C A G T C A G T C A G T C A G T C A G T C A G T C A G T C A G T C A G T C A G T C A G T C | 1e-9 | -2.223e+01 | 3.44% | 0.74% | 34.0bp (0.0bp) | PB0097.1\_Zfp281\_1/Jaspar(0.491) More Information | Similar Motifs Found | motif file (matrix) |
| 63 \* | A T C G T G A C C T G A A C T G T A C G T A G C C G T A A T C G C A G T A C T G T A C G A C T G T A C G C A G T A C T G | 1e-9 | -2.113e+01 | 5.51% | 2.07% | 58.5bp (45.5bp) | ELF3/MA0640.2/Jaspar(0.648) More Information | Similar Motifs Found | motif file (matrix) |
| 64 \* | A C G T C G T A A C T G A C G T C G T A A C G T | 1e-9 | -2.090e+01 | 4.82% | 1.76% | 51.4bp (27.4bp) | PB0181.1\_Spdef\_2/Jaspar(0.663) More Information | Similar Motifs Found | motif file (matrix) |
| 65 \* | A T G C A G T C A G T C A C T G A C G T A C G T | 1e-8 | -1.995e+01 | 5.37% | 1.97% | 50.5bp (27.0bp) | BMYB(HTH)/Hela-BMYB-ChIP-Seq(GSE27030)/Homer(0.828) More Information | Similar Motifs Found | motif file (matrix) |
| 66 \* | C G T A A C G T C G T A A G T C A C T G A C G T A C T G A G T C | 1e-7 | -1.835e+01 | 3.86% | 1.13% | 51.4bp (62.6bp) | HIF-1b(HLH)/T47D-HIF1b-ChIP-Seq(GSE59937)/Homer(0.901) More Information | Similar Motifs Found | motif file (matrix) |
| 67 \* | A C T G A G T C C G T A A C G T A C T G A G T C | 1e-7 | -1.751e+01 | 3.03% | 0.96% | 50.8bp (6.5bp) | NRF(NRF)/Promoter/Homer(0.754) More Information | Similar Motifs Found | motif file (matrix) |
| 68 \* | T A C G G A T C T A G C A C T G A T C G T A G C G A C T T A G C G T A C A T C G A C G T T C A G T A C G T A G C C A G T A C T G C T G A T A C G T A C G T G C A C T A G A T C G T G A C C T A G T A C G G A C T A C G T G C A T G A T C A C G T A C G T A G C T T A G C A T C G A T C G C A T G G A C T A C G T A T G C G A C T A C T G A T G C A T G C A C T G T A C G A G T C A C G T A T G C G A T C A C T G | 1e-5 | -1.319e+01 | 2.62% | 0.95% | 44.3bp (24.9bp) | PB0076.1\_Sp4\_1/Jaspar(0.397) More Information | Similar Motifs Found | motif file (matrix) |
| 69 \* | A G T C A C G T C G T A A C T G A G T C A G T C | 1e-3 | -7.773e+00 | 4.68% | 2.56% | 57.8bp (40.3bp) | Smad4/MA1153.1/Jaspar(0.706) More Information | Similar Motifs Found | motif file (matrix) |
| 70 \* | A C G T A G T C A C T G A C T G A C G T A C T G | 1e-3 | -7.042e+00 | 1.93% | 0.85% | 46.9bp (22.0bp) | ZBTB7C/MA0695.1/Jaspar(0.713) More Information | Similar Motifs Found | motif file (matrix) |
