# Supplementary material for: Global hypo-methylation in a proportion of glioblastoma enriched for an astrocytic signature is associated with increased invasion and altered immune landscape
Source: eLife. 2022 Nov 22;11:e77335. doi: 10.7554/eLife.77335 (PMC9681209; doi:10.7554/eLife.77335)
Supplement: Figure 2—source data 1. [file elife-77335-fig2-data1.zip › Figure_2_source_data_1/Figure_2I_J/knownResults.html]

/data/Blizard-MarinoLab/Nicola\_Pomella/Motifs\_James/201117\_1/ - Homer Known Motif Enrichment Results


# Homer Known Motif Enrichment Results (/data/Blizard-MarinoLab/Nicola\_Pomella/Motifs\_James/201117\_1/)

Homer *de novo* Motif Results  
Gene Ontology Enrichment Results  
Known Motif Enrichment Results (txt file)  
Total Target Sequences = 726, Total Background Sequences = 282

|  |  |  |  |  |  |  |  |  |  |  |  |
| --- | --- | --- | --- | --- | --- | --- | --- | --- | --- | --- | --- |
| Rank | Motif | Name | P-value | log P-pvalue | q-value (Benjamini) | # Target Sequences with Motif | % of Targets Sequences with Motif | # Background Sequences with Motif | % of Background Sequences with Motif | Motif File | SVG |
| 1 | C T A G A C T G T G C A G T C A A T G C C G T A A T C G A T G C A G T C C T A G | ZNF341(Zf)/EBV-ZNF341-ChIP-Seq(GSE113194)/Homer | 1e-10 | -2.509e+01 | 0.0000 | 48.0 | 6.61% | 6.7 | 2.38% | motif file (matrix) | svg |
| 2 | T G A C C G T A C T G A A C T G A C T G G A C T G A T C T G C A G T A C T A C G | SF1(NR)/H295R-Nr5a1-ChIP-Seq(GSE44220)/Homer | 1e-9 | -2.111e+01 | 0.0000 | 30.0 | 4.13% | 3.4 | 1.22% | motif file (matrix) | svg |
| 3 | A C G T G A C T T A G C C G T A C T G A C A T G C T A G G A C T G A T C C G T A | Nr5a2(NR)/Pancreas-LRH1-ChIP-Seq(GSE34295)/Homer | 1e-8 | -2.008e+01 | 0.0000 | 48.0 | 6.61% | 8.0 | 2.82% | motif file (matrix) | svg |
| 4 | T A G C G T A C A G T C G T A C C G A T A G T C A G T C A G T C A G T C A G T C C G T A G A T C | Zfp281(Zf)/ES-Zfp281-ChIP-Seq(GSE81042)/Homer | 1e-8 | -1.999e+01 | 0.0000 | 17.0 | 2.34% | 0.5 | 0.18% | motif file (matrix) | svg |
| 5 | C A T G A C T G A G C T A T G C C G T A A T G C G T A C G A C T T A C G C T G A A C T G A C T G G C A T A T G C C T G A | THRb(NR)/HepG2-THRb.Flag-ChIP-Seq(Encode)/Homer | 1e-8 | -1.889e+01 | 0.0000 | 51.0 | 7.02% | 8.2 | 2.90% | motif file (matrix) | svg |
| 6 | A G T C G A C T C A G T A C T G C T A G T G A C G C T A A T G C G C A T A T C G C G A T A C T G G A T C G T A C G T C A C T G A | NF1(CTF)/LNCAP-NF1-ChIP-Seq(Unpublished)/Homer | 1e-7 | -1.840e+01 | 0.0000 | 33.0 | 4.55% | 4.6 | 1.64% | motif file (matrix) | svg |
| 7 | A T G C T A C G A G C T T G C A C G T A C G A T A C G T C T G A | DLX5(Homeobox)/BasalGanglia-Dlx5-ChIP-seq(GSE124936)/Homer | 1e-7 | -1.613e+01 | 0.0000 | 52.0 | 7.16% | 9.8 | 3.47% | motif file (matrix) | svg |
| 8 | T C G A T A G C T G A C C T G A A G T C A C T G G A C T C A T G | c-Myc(bHLH)/LNCAP-cMyc-ChIP-Seq(Unpublished)/Homer | 1e-6 | -1.444e+01 | 0.0000 | 34.0 | 4.68% | 5.8 | 2.05% | motif file (matrix) | svg |
| 9 | T A C G T C G A T A G C A G T C C G T A A G T C C T A G G C A T A C T G A T C G | n-Myc(bHLH)/mES-nMyc-ChIP-Seq(GSE11431)/Homer | 1e-6 | -1.429e+01 | 0.0000 | 46.0 | 6.34% | 8.1 | 2.87% | motif file (matrix) | svg |
| 10 | A G T C C G A T A C T G A T C G T G A C G C T A C A T G A T C G T G A C C G A T A C T G T A G C G T A C G T C A | Tlx?(NR)/NPC-H3K4me1-ChIP-Seq(GSE16256)/Homer | 1e-5 | -1.376e+01 | 0.0000 | 29.0 | 3.99% | 4.6 | 1.62% | motif file (matrix) | svg |
| 11 | T G A C C T A G T C A G G T C A C G T A T C A G C G A T T C A G T C G A T G C A C T G A T A G C | PU.1-IRF(ETS:IRF)/Bcell-PU.1-ChIP-Seq(GSE21512)/Homer | 1e-5 | -1.363e+01 | 0.0000 | 94.0 | 12.95% | 22.8 | 8.09% | motif file (matrix) | svg |
| 12 | T C A G G C T A T C A G C A G T T G A C G T C A A G T C A T C G T G C A G T A C C A G T G A T C | Npas4(bHLH)/Neuron-Npas4-ChIP-Seq(GSE127793)/Homer | 1e-5 | -1.332e+01 | 0.0001 | 41.0 | 5.65% | 7.4 | 2.60% | motif file (matrix) | svg |
| 13 | A G T C A T C G G C A T C A T G A C T G A T C G C G A T C T A G A C T G A G C T T G A C G A C T | Gli2(Zf)/GM2-Gli2-ChIP-Chip(GSE112702)/Homer | 1e-5 | -1.185e+01 | 0.0002 | 18.0 | 2.48% | 2.2 | 0.78% | motif file (matrix) | svg |
| 14 | A C G T G A C T A T G C G C T A C T G A C T A G A C T G G A C T A G T C C G T A | Nr5a2(NR)/mES-Nr5a2-ChIP-Seq(GSE19019)/Homer | 1e-4 | -1.148e+01 | 0.0003 | 31.0 | 4.27% | 5.5 | 1.95% | motif file (matrix) | svg |
| 15 | C T A G A C T G T G C A A G T C C G T A A C T G A C T G A C G T C T A G C G A T T A C G A G T C | ZEB2(Zf)/SNU398-ZEB2-ChIP-Seq(GSE103048)/Homer | 1e-4 | -1.079e+01 | 0.0006 | 76.0 | 10.47% | 18.1 | 6.42% | motif file (matrix) | svg |
| 16 | C G T A C G A T G A C T G A C T T G A C C T G A A T G C C T G A T A G C A G T C A C G T T C G A C A T G T A C G G A C T A T C G G A C T A C G T C T G A T C G A C G T A | Brachyury(T-box)/Mesoendoderm-Brachyury-ChIP-exo(GSE54963)/Homer | 1e-4 | -1.056e+01 | 0.0007 | 17.0 | 2.34% | 2.9 | 1.02% | motif file (matrix) | svg |
| 17 | T C G A C A G T A C T G A G C T C G T A C G T A A C G T A C G T C T G A T A G C | Dlx3(Homeobox)/Kerainocytes-Dlx3-ChIP-Seq(GSE89884)/Homer | 1e-4 | -1.052e+01 | 0.0007 | 45.0 | 6.20% | 9.2 | 3.24% | motif file (matrix) | svg |
| 18 | A C T G A G C T A G T C G T C A A G C T T C A G A T G C G A T C G C A T A T C G T C G A T A G C C G A T C A T G T A G C | Pax8(Paired,Homeobox)/Thyroid-Pax8-ChIP-Seq(GSE26938)/Homer | 1e-4 | -9.872e+00 | 0.0013 | 21.0 | 2.89% | 3.7 | 1.33% | motif file (matrix) | svg |
| 19 | C T G A C A G T C T G A A G T C C T A G G A C T A T C G G T A C | HIF-1b(HLH)/T47D-HIF1b-ChIP-Seq(GSE59937)/Homer | 1e-4 | -9.819e+00 | 0.0013 | 71.0 | 9.78% | 17.8 | 6.31% | motif file (matrix) | svg |
| 20 | T C A G T C A G T A G C A G T C C T G A A G T C C T A G A C G T A C T G A T C G | c-Myc(bHLH)/mES-cMyc-ChIP-Seq(GSE11431)/Homer | 1e-4 | -9.667e+00 | 0.0014 | 29.0 | 3.99% | 5.9 | 2.09% | motif file (matrix) | svg |
| 21 | A T C G A T G C A G T C T A G C G A C T T C G A G C T A G C A T A G C T C T G A | DLX1(Homeobox)/BasalGanglia-Dlx1-ChIP-seq(GSE124936)/Homer | 1e-4 | -9.607e+00 | 0.0014 | 102.0 | 14.05% | 27.8 | 9.86% | motif file (matrix) | svg |
| 22 | G A C T A G C T G T A C G A C T C T G A A C T G G T C A C T G A A T G C T A C G G A C T A C G T A G T C G A C T C T G A | HRE(HSF)/Striatum-HSF1-ChIP-Seq(GSE38000)/Homer | 1e-4 | -9.483e+00 | 0.0015 | 11.0 | 1.52% | 1.6 | 0.56% | motif file (matrix) | svg |
| 23 | T C G A T G A C A G T C C G T A A G T C C T A G A C G T A C T G A C T G A G C T A G T C G C A T | Max(bHLH)/K562-Max-ChIP-Seq(GSE31477)/Homer | 1e-3 | -8.790e+00 | 0.0029 | 39.0 | 5.37% | 8.5 | 3.00% | motif file (matrix) | svg |
| 24 | C T A G C A T G A C G T A G T C G C T A A G C T A G T C A G C T T C A G C T G A A C T G C A T G G C A T A T G C C G T A | THRa(NR)/C17.2-THRa-ChIP-Seq(GSE38347)/Homer | 1e-3 | -8.212e+00 | 0.0048 | 31.0 | 4.27% | 7.0 | 2.47% | motif file (matrix) | svg |
| 25 | T A C G T C A G A G C T A T G C C G T A A G T C T C A G A C G T A C T G T C G A | USF1(bHLH)/GM12878-Usf1-ChIP-Seq(GSE32465)/Homer | 1e-3 | -8.212e+00 | 0.0048 | 31.0 | 4.27% | 6.3 | 2.23% | motif file (matrix) | svg |
| 26 | A C T G T C A G A G C T G A C T C A T G A G T C A G T C G C T A C G A T C T A G T C A G G T A C C T G A T C G A | Rfx1(HTH)/NPC-H3K4me1-ChIP-Seq(GSE16256)/Homer | 1e-3 | -8.153e+00 | 0.0049 | 15.0 | 2.07% | 2.9 | 1.03% | motif file (matrix) | svg |
| 27 | A G T C A T C G G C A T C T A G A C T G T A C G C G A T T C A G C A T G A G C T T A G C G A T C | GLI3(Zf)/Limb-GLI3-ChIP-Chip(GSE11077)/Homer | 1e-3 | -7.997e+00 | 0.0055 | 10.0 | 1.38% | 0.9 | 0.32% | motif file (matrix) | svg |
| 28 | T A G C T A G C G A C T C T A G A G C T A G T C G T C A T G C A A C G T A T G C G C T A T G C A | Pbx3(Homeobox)/GM12878-PBX3-ChIP-Seq(GSE32465)/Homer | 1e-3 | -7.836e+00 | 0.0060 | 19.0 | 2.62% | 3.2 | 1.14% | motif file (matrix) | svg |
| 29 | C A G T G A C T C G T A G C T A G T A C G A T C G T A C G A C T A G C T A C G T T G A C C G T A C A G T A C G T A T G C | ZNF652/HepG2-ZNF652.Flag-ChIP-Seq(Encode)/Homer | 1e-3 | -7.836e+00 | 0.0060 | 19.0 | 2.62% | 3.1 | 1.10% | motif file (matrix) | svg |
| 30 | A T C G A T C G A T G C G A C T T C G A C G T A G C A T A G C T C T G A T A C G | DLX2(Homeobox)/BasalGanglia-Dlx2-ChIP-seq(GSE124936)/Homer | 1e-3 | -7.572e+00 | 0.0076 | 118.0 | 16.25% | 34.7 | 12.28% | motif file (matrix) | svg |
| 31 | C G T A T A G C T A G C T G C A A C T G C T A G C G T A C G T A T C A G G A C T | EHF(ETS)/LoVo-EHF-ChIP-Seq(GSE49402)/Homer | 1e-3 | -7.504e+00 | 0.0078 | 82.0 | 11.29% | 22.5 | 7.98% | motif file (matrix) | svg |
| 32 | C G T A T A C G T C G A A C T G A C T G C G T A C G T A T A C G A G C T T A C G | PU.1(ETS)/ThioMac-PU.1-ChIP-Seq(GSE21512)/Homer | 1e-3 | -7.468e+00 | 0.0079 | 30.0 | 4.13% | 6.6 | 2.32% | motif file (matrix) | svg |
| 33 | C T A G T A C G G A C T T G C A T G C A C G A T T A C G C T G A T C G A C T G A | Hoxa10(Homeobox)/ChickenMSG-Hoxa10.Flag-ChIP-Seq(GSE86088)/Homer | 1e-3 | -7.440e+00 | 0.0079 | 37.0 | 5.10% | 9.0 | 3.18% | motif file (matrix) | svg |
| 34 | A C T G G A T C C T G A A T C G A G T C T A G C C T G A C G T A T A C G A G T C C T A G C A G T T C A G T C G A T G A C G A T C | PAX5(Paired,Homeobox)/GM12878-PAX5-ChIP-Seq(GSE32465)/Homer | 1e-3 | -7.199e+00 | 0.0097 | 26.0 | 3.58% | 5.7 | 2.04% | motif file (matrix) | svg |
| 35 | A G C T G C A T A C T G A C G T A G T C A C G T C T A G T A C G | Smad3(MAD)/NPC-Smad3-ChIP-Seq(GSE36673)/Homer | 1e-3 | -6.961e+00 | 0.0119 | 190.0 | 26.17% | 60.3 | 21.37% | motif file (matrix) | svg |
| 36 | T C G A C T A G A G T C A G T C C G T A C G T A A C G T T A G C T C A G T A C G | NFY(CCAAT)/Promoter/Homer | 1e-2 | -6.815e+00 | 0.0134 | 46.0 | 6.34% | 11.9 | 4.22% | motif file (matrix) | svg |
| 37 | T C A G C A T G C A T G A C T G A C T G A G C T A C T G A C G T A C T G C A G T A T G C A G T C | KLF10(Zf)/HEK293-KLF10.GFP-ChIP-Seq(GSE58341)/Homer | 1e-2 | -6.803e+00 | 0.0134 | 36.0 | 4.96% | 8.9 | 3.15% | motif file (matrix) | svg |
| 38 | T C G A A C T G C A T G A G C T A G T C C G T A C T G A C T A G A C T G C G A T A T G C C T G A | RAR:RXR(NR),DR0/ES-RAR-ChIP-Seq(GSE56893)/Homer | 1e-2 | -6.603e+00 | 0.0157 | 9.0 | 1.24% | 0.5 | 0.18% | motif file (matrix) | svg |
| 39 | C A T G G T A C C G T A A G T C C T A G A C G T A C T G G T A C A G T C A G C T | bHLHE40(bHLH)/HepG2-BHLHE40-ChIP-Seq(GSE31477)/Homer | 1e-2 | -6.447e+00 | 0.0179 | 25.0 | 3.44% | 5.8 | 2.07% | motif file (matrix) | svg |
| 40 | T C G A T C G A T A C G G A T C G T C A G T A C C G A T A G C T G T C A T G C A | Nkx3.1(Homeobox)/LNCaP-Nkx3.1-ChIP-Seq(GSE28264)/Homer | 1e-2 | -6.225e+00 | 0.0218 | 168.0 | 23.14% | 53.9 | 19.09% | motif file (matrix) | svg |
| 41 | T G A C T A G C T C A G T C G A T C G A C G T A A G T C C G T A C G T A C G A T C T A G T A C G | Sox7(HMG)/ESC-Sox7-ChIP-Seq(GSE133899)/Homer | 1e-2 | -6.182e+00 | 0.0222 | 21.0 | 2.89% | 4.4 | 1.56% | motif file (matrix) | svg |
| 42 | C T A G C T A G A G T C T C A G A C T G A C G T A C G T C T G A | MYB(HTH)/ERMYB-Myb-ChIPSeq(GSE22095)/Homer | 1e-2 | -6.161e+00 | 0.0222 | 114.0 | 15.70% | 34.9 | 12.36% | motif file (matrix) | svg |
| 43 | C T G A C T A G T C G A C G T A A T G C C G T A A T C G C G A T T A G C G C A T A T C G G C A T A G C T G A T C G A C T A G C T | ARE(NR)/LNCAP-AR-ChIP-Seq(GSE27824)/Homer | 1e-2 | -5.997e+00 | 0.0249 | 13.0 | 1.79% | 2.6 | 0.91% | motif file (matrix) | svg |
| 44 | T C A G A T C G G A C T A C T G G A C T C A G T C T A G C G T A G T A C C G T A C T A G A T C G | Tbx20(T-box)/Heart-Tbx20-ChIP-Seq(GSE29636)/Homer | 1e-2 | -5.997e+00 | 0.0249 | 13.0 | 1.79% | 2.2 | 0.78% | motif file (matrix) | svg |
| 45 | T A G C T C A G C A T G G C A T A G C T C G A T A T G C C G T A C G T A G T C A | CHR(?)/Hela-CellCycle-Expression/Homer | 1e-2 | -5.674e+00 | 0.0336 | 47.0 | 6.47% | 12.3 | 4.36% | motif file (matrix) | svg |
| 46 | A C T G C A T G T C G A A C G T A C T G C G T A A T C G A C G T G T A C C G T A G A C T A G T C | Fos(bZIP)/TSC-Fos-ChIP-Seq(GSE110950)/Homer | 1e-2 | -5.611e+00 | 0.0350 | 34.0 | 4.68% | 8.1 | 2.86% | motif file (matrix) | svg |
| 47 | A C G T A G T C A G T C C G A T A C G T A C G T A C T G A C G T A T G C G A C T A C T G T A C G | Sox21(HMG)/ESC-SOX21-ChIP-Seq(GSE110505)/Homer | 1e-2 | -5.587e+00 | 0.0351 | 92.0 | 12.67% | 27.3 | 9.68% | motif file (matrix) | svg |
| 48 | T A G C G C A T A G T C G A T C A T G C G A C T C T A G A C T G A C T G C T G A A C T G C T A G A G T C T G A C C G A T | GLIS3(Zf)/Thyroid-Glis3.GFP-ChIP-Seq(GSE103297)/Homer | 1e-2 | -5.513e+00 | 0.0370 | 112.0 | 15.43% | 34.8 | 12.31% | motif file (matrix) | svg |
| 49 | A T G C A T G C A T C G T A C G A G C T A G T C G C T A A G T C T C A G G A C T A C T G T C G A | E-box(bHLH)/Promoter/Homer | 1e-2 | -5.308e+00 | 0.0427 | 8.0 | 1.10% | 1.7 | 0.62% | motif file (matrix) | svg |
| 50 | T C A G C T G A C G T A C G T A T A C G G C A T C T A G C T G A C G T A C G T A T A C G G A C T | IRF1(IRF)/PBMC-IRF1-ChIP-Seq(GSE43036)/Homer | 1e-2 | -5.308e+00 | 0.0427 | 8.0 | 1.10% | 1.9 | 0.68% | motif file (matrix) | svg |
| 51 | T A C G C G T A T C A G G A C T C T A G A C T G C A G T T A G C T C G A A C G T G T A C C T A G A G T C A G T C G A T C | ZNF669(Zf)/HEK293-ZNF669.GFP-ChIP-Seq(GSE58341)/Homer | 1e-2 | -5.308e+00 | 0.0427 | 8.0 | 1.10% | 0.4 | 0.15% | motif file (matrix) | svg |
| 52 | A G T C T G C A T C G A C T G A A C T G C A T G A C G T A T G C G T C A T A C G | Erra(NR)/HepG2-Erra-ChIP-Seq(GSE31477)/Homer | 1e-2 | -5.202e+00 | 0.0466 | 128.0 | 17.63% | 40.6 | 14.37% | motif file (matrix) | svg |
| 53 | T A C G A T G C G A C T A C T G A G C T A G T C G T C A T G C A A C G T A G T C G C T A T G C A | Pknox1(Homeobox)/ES-Prep1-ChIP-Seq(GSE63282)/Homer | 1e-2 | -5.158e+00 | 0.0477 | 16.0 | 2.20% | 3.2 | 1.14% | motif file (matrix) | svg |
| 54 | C T G A A C G T A C G T A C G T A G T C G A C T C G A T C T G A A C T G C G T A C G T A T C G A | STAT5(Stat)/mCD4+-Stat5-ChIP-Seq(GSE12346)/Homer | 1e-2 | -5.057e+00 | 0.0509 | 23.0 | 3.17% | 5.7 | 2.00% | motif file (matrix) | svg |
| 55 | C T A G C A T G C A T G T A C G A G T C G C A T A G C T C T A G A C G T A G T C G A C T A C T G A C T G A C T G T C G A | Zfp809(Zf)/ES-Zfp809-ChIP-Seq(GSE70799)/Homer | 1e-2 | -5.057e+00 | 0.0509 | 23.0 | 3.17% | 6.0 | 2.12% | motif file (matrix) | svg |
| 56 | G C T A C G T A A G T C A C G T T C G A T A C G A C T G A G C T A G T C T C G A | RORgt(NR)/EL4-RORgt.Flag-ChIP-Seq(GSE56019)/Homer | 1e-2 | -5.022e+00 | 0.0509 | 12.0 | 1.65% | 2.6 | 0.93% | motif file (matrix) | svg |
| 57 | G C T A C G T A A G T C A C G T T C G A T A C G A C T G A G C T A G T C T C G A | RORgt(NR)/EL4-RORgt.Flag-ChIP-Seq(GSE56019)/Homer | 1e-2 | -5.022e+00 | 0.0509 | 12.0 | 1.65% | 2.6 | 0.93% | motif file (matrix) | svg |
| 58 | C G T A G A C T C G A T A T C G G T A C G C A T C A T G C G T A T A C G G C A T G T A C C G T A C A T G A T G C G C T A C T A G G C A T G C A T G C A T G A C T | MafB(bZIP)/BMM-Mafb-ChIP-Seq(GSE75722)/Homer | 1e-2 | -4.702e+00 | 0.0689 | 19.0 | 2.62% | 4.3 | 1.52% | motif file (matrix) | svg |
| 59 | A G C T A G T C A G T C A C G T C T A G A C G T A C G T A C G T C G T A A G T C G A T C C G T A | FOXP1(Forkhead)/H9-FOXP1-ChIP-Seq(GSE31006)/Homer | 1e-2 | -4.648e+00 | 0.0715 | 29.0 | 3.99% | 7.9 | 2.79% | motif file (matrix) | svg |
