# Supplementary material for: Global hypo-methylation in a proportion of glioblastoma enriched for an astrocytic signature is associated with increased invasion and altered immune landscape
Source: eLife. 2022 Nov 22;11:e77335. doi: 10.7554/eLife.77335 (PMC9681209; doi:10.7554/eLife.77335)
Supplement: Figure 2—source data 1. [file elife-77335-fig2-data1.zip › Figure_2_source_data_1/Figure_2B/homerResults.html]

/data/Blizard-MarinoLab/Nicola\_Pomella/Motifs\_Sara/JamesAnalysis\_Size\_200\_Motifs\_6to50// - Homer de novo Motif Results


# Homer *de novo* Motif Results (/data/Blizard-MarinoLab/Nicola\_Pomella/Motifs\_Sara/JamesAnalysis\_Size\_200\_Motifs\_6to50//)

Known Motif Enrichment Results  
Gene Ontology Enrichment Results  
If Homer is having trouble matching a motif to a known motif, try copy/pasting the matrix file into
STAMP  
More information on motif finding results: HOMER
| Description of Results
| Tips
  
Total target sequences = 2310  
Total background sequences = 353  
\* - possible false positive  

|  |  |  |  |  |  |  |  |  |
| --- | --- | --- | --- | --- | --- | --- | --- | --- |
| Rank | Motif | P-value | log P-pvalue | % of Targets | % of Background | STD(Bg STD) | Best Match/Details | Motif File |
| 1 | C T G A A C G T G T C A A G T C T G C A A C T G A G C T A C G T | 1e-91 | -2.096e+02 | 4.72% | 0.30% | 53.0bp (0.0bp) | ZBTB32/MA1580.1/Jaspar(0.796) More Information | Similar Motifs Found | motif file (matrix) |
| 2 | A C G T A T C G A C G T A C T G A C T G A C T G A T G C C G T A | 1e-88 | -2.039e+02 | 4.63% | 0.54% | 56.8bp (27.6bp) | HIC2/MA0738.1/Jaspar(0.783) More Information | Similar Motifs Found | motif file (matrix) |
| 3 | C G A T A C T G A G T C G A C T A G T C G A T C G A T C G C A T A C T G A G C T A G C T A G C T | 1e-84 | -1.954e+02 | 4.50% | 0.51% | 56.1bp (63.1bp) | IKZF1/MA1508.1/Jaspar(0.774) More Information | Similar Motifs Found | motif file (matrix) |
| 4 | T G A C G C A T A C T G T A C G T A C G G A T C G A T C T A G C G T C A T A C G T C G A C T A G T C A G T A G C G A T C | 1e-82 | -1.898e+02 | 4.41% | 0.53% | 54.4bp (16.9bp) | Plagl1/MA1615.1/Jaspar(0.616) More Information | Similar Motifs Found | motif file (matrix) |
| 5 | A G T C A C G T A C G T A C T G A C T G A G C T A C G T A G T C | 1e-73 | -1.692e+02 | 5.28% | 0.72% | 59.0bp (33.4bp) | Sox3/MA0514.1/Jaspar(0.692) More Information | Similar Motifs Found | motif file (matrix) |
| 6 | C T G A A C T G C T G A C G T A C T A G A C T G C G T A A C T G A G T C A G C T | 1e-72 | -1.678e+02 | 4.07% | 0.48% | 52.6bp (15.7bp) | POL013.1\_MED-1/Jaspar(0.625) More Information | Similar Motifs Found | motif file (matrix) |
| 7 | A C G T C T A G A G T C G T A C C G T A A G C T G C T A G A T C | 1e-71 | -1.651e+02 | 4.02% | 0.56% | 54.4bp (63.7bp) | Tgif2(Homeobox)/mES-Tgif2-ChIP-Seq(GSE55404)/Homer(0.704) More Information | Similar Motifs Found | motif file (matrix) |
| 8 | A C G T A C T G A C T G A G T C A G T C T G A C A C G T A C T G | 1e-70 | -1.630e+02 | 6.92% | 1.27% | 54.3bp (55.7bp) | SF1(NR)/H295R-Nr5a1-ChIP-Seq(GSE44220)/Homer(0.765) More Information | Similar Motifs Found | motif file (matrix) |
| 9 | A C G T A G T C A G T C A C G T A C G T A C G T A G T C A C G T A C T G A G T C A C G T A C G T | 1e-69 | -1.601e+02 | 5.11% | 0.83% | 54.8bp (52.5bp) | Stat2/MA1623.1/Jaspar(0.696) More Information | Similar Motifs Found | motif file (matrix) |
| 10 | A G C T A G T C G C A T C A T G C T A G T C A G T C G A T G C A T A G C G T A C G T A C G T C A | 1e-67 | -1.556e+02 | 5.02% | 0.73% | 55.0bp (27.3bp) | RBPJ/MA1116.1/Jaspar(0.776) More Information | Similar Motifs Found | motif file (matrix) |
| 11 | A T G C A T C G A T G C G T A C G C A T T A C G T A G C C G T A A T C G A T G C A G T C G A T C G C A T A C T G A T C G | 1e-67 | -1.556e+02 | 5.02% | 0.71% | 54.0bp (52.3bp) | Zic1::Zic2/MA1628.1/Jaspar(0.574) More Information | Similar Motifs Found | motif file (matrix) |
| 12 | A G T C C G T A A C T G C G T A A C T G C G T A A C T G A C T G A G T C A G T C | 1e-67 | -1.544e+02 | 3.85% | 0.54% | 50.5bp (45.9bp) | ZNF768(Zf)/Rajj-ZNF768-ChIP-Seq(GSE111879)/Homer(0.812) More Information | Similar Motifs Found | motif file (matrix) |
| 13 | G T A C A T G C A G T C T G C A A C G T A C G T A C G T C G T A | 1e-65 | -1.512e+02 | 4.93% | 0.61% | 51.2bp (65.3bp) | BARHL2/MA0635.1/Jaspar(0.759) More Information | Similar Motifs Found | motif file (matrix) |
| 14 | C T A G T A C G C T G A T A C G T A G C G A C T T C A G C A G T C T A G T C A G C T G A T A C G C T G A C T A G T C A G | 1e-60 | -1.402e+02 | 4.72% | 0.79% | 54.1bp (62.5bp) | ZNF263/MA0528.2/Jaspar(0.593) More Information | Similar Motifs Found | motif file (matrix) |
| 15 | G T A C G C T A G C T A G C A T C G A T G C T A G C A T C G A T G C A T G C A T G C T A C G A T | 1e-60 | -1.387e+02 | 3.59% | 0.56% | 55.9bp (54.2bp) | MF0010.1\_Homeobox\_class/Jaspar(0.760) More Information | Similar Motifs Found | motif file (matrix) |
| 16 | T A C G C T A G A G C T T C G A G T A C T G A C C G A T T G C A C G T A C T A G | 1e-59 | -1.361e+02 | 3.55% | 0.25% | 55.8bp (0.0bp) | PB0159.1\_Rfx4\_2/Jaspar(0.645) More Information | Similar Motifs Found | motif file (matrix) |
| 17 | C T G A A C G T A T G C A G T C C G T A A C G T C G T A A C G T A C G T A G T C A G C T A C G T | 1e-59 | -1.361e+02 | 3.55% | 0.21% | 52.3bp (0.0bp) | Pit1(Homeobox)/GCrat-Pit1-ChIP-Seq(GSE58009)/Homer(0.762) More Information | Similar Motifs Found | motif file (matrix) |
| 18 | G C A T A C G T T A G C G A T C G A C T G A C T A C G T C T A G A T G C G C A T | 1e-59 | -1.359e+02 | 4.63% | 0.81% | 59.2bp (9.7bp) | PB0083.1\_Tcf7\_1/Jaspar(0.689) More Information | Similar Motifs Found | motif file (matrix) |
| 19 | G T A C G T A C G T C A C T G A T C A G C A G T A T C G A G C T A G C T A T G C | 1e-58 | -1.353e+02 | 10.00% | 2.95% | 56.3bp (53.6bp) | Prdm15/MA1616.1/Jaspar(0.691) More Information | Similar Motifs Found | motif file (matrix) |
| 20 | A T G C G A T C A T G C A C G T T C A G C G T A C G A T C A T G | 1e-57 | -1.335e+02 | 3.50% | 0.54% | 60.3bp (27.6bp) | Isl1/MA1608.1/Jaspar(0.741) More Information | Similar Motifs Found | motif file (matrix) |
| 21 | G C A T T A G C G T C A A T G C G T A C G A C T G T C A A G C T T A G C G T C A | 1e-57 | -1.334e+02 | 6.92% | 1.63% | 53.2bp (49.9bp) | PKNOX2/MA0783.1/Jaspar(0.699) More Information | Similar Motifs Found | motif file (matrix) |
| 22 | T A G C C G T A A T C G T A G C T G A C T G A C G C T A A T C G C A G T A T C G A T C G T A C G | 1e-57 | -1.316e+02 | 4.54% | 0.79% | 58.1bp (63.2bp) | ZNF416(Zf)/HEK293-ZNF416.GFP-ChIP-Seq(GSE58341)/Homer(0.726) More Information | Similar Motifs Found | motif file (matrix) |
| 23 | T A C G T C G A T A C G T G A C G C T A A T C G T A G C G A T C G A T C G A T C G A C T A T G C | 1e-54 | -1.253e+02 | 4.41% | 0.78% | 56.8bp (33.4bp) | MAZ/MA1522.1/Jaspar(0.643) More Information | Similar Motifs Found | motif file (matrix) |
| 24 | T G C A C T G A T A G C C A T G A T C G C A G T A T C G C G A T C A T G T C A G C A G T C T G A | 1e-53 | -1.232e+02 | 4.37% | 0.78% | 52.4bp (43.5bp) | EKLF(Zf)/Erythrocyte-Klf1-ChIP-Seq(GSE20478)/Homer(0.772) More Information | Similar Motifs Found | motif file (matrix) |
| 25 | A C G T A C G T A C G T A C G T A C G T A C G T A G T C A C G T C G T A A C G T | 1e-51 | -1.185e+02 | 5.11% | 1.09% | 52.2bp (46.1bp) | PB0192.1\_Tcfap2e\_2/Jaspar(0.752) More Information | Similar Motifs Found | motif file (matrix) |
| 26 | C A T G T C A G A T C G T C G A T A G C C G T A A C T G T A C G A T G C G C T A A C T G T A C G | 1e-45 | -1.040e+02 | 4.76% | 1.11% | 54.1bp (1.6bp) | GFY(?)/Promoter/Homer(0.592) More Information | Similar Motifs Found | motif file (matrix) |
| 27 | A G T C C G T A A C T G A C T G A C G T A C T G A G T C A G T C A C G T A C T G | 1e-45 | -1.038e+02 | 2.99% | 0.00% | 54.7bp (0.0bp) | TCF4/MA0830.2/Jaspar(0.795) More Information | Similar Motifs Found | motif file (matrix) |
| 28 | C G T A A C G T C G T A A G T C A C G T A C G T A C G T A C G T A C G T C G T A | 1e-45 | -1.038e+02 | 2.99% | 0.37% | 55.3bp (38.7bp) | Dmrt1/MA1603.1/Jaspar(0.725) More Information | Similar Motifs Found | motif file (matrix) |
| 29 | A G T C G T A C G T A C C G T A A C G T A C T G C T A G C G T A A G T C G T A C C G T A A C T G | 1e-44 | -1.034e+02 | 6.10% | 1.66% | 54.5bp (49.3bp) | PB0200.1\_Zfp187\_2/Jaspar(0.600) More Information | Similar Motifs Found | motif file (matrix) |
| 30 | A C T G A T C G A T C G C T G A T C A G A C G T A T C G C T A G | 1e-44 | -1.029e+02 | 3.94% | 0.73% | 56.6bp (44.7bp) | ZNF148/MA1653.1/Jaspar(0.783) More Information | Similar Motifs Found | motif file (matrix) |
| 31 | A T C G A T C G C T A G A C T G G T C A A C G T A G T C C G T A | 1e-43 | -1.005e+02 | 4.67% | 0.94% | 55.8bp (37.7bp) | MZF1/MA0056.2/Jaspar(0.758) More Information | Similar Motifs Found | motif file (matrix) |
| 32 | A G T C A C T G A C G T A C G T A G T C A C T G A C G T A C T G | 1e-43 | -9.908e+01 | 2.90% | 0.45% | 55.6bp (21.3bp) | Npas4(bHLH)/Neuron-Npas4-ChIP-Seq(GSE127793)/Homer(0.698) More Information | Similar Motifs Found | motif file (matrix) |
| 33 | A C G T A G T C A C G T A C G T A G T C A G T C A G T C A C G T A G T C A C G T A C T G A C T G | 1e-42 | -9.892e+01 | 3.85% | 0.81% | 49.1bp (48.3bp) | ZSCAN22(Zf)/HEK293-ZSCAN22.GFP-ChIP-Seq(GSE58341)/Homer(0.659) More Information | Similar Motifs Found | motif file (matrix) |
| 34 | A C G T A C T G A G T C A C T G A C G T C G T A A C T G A G T C | 1e-42 | -9.876e+01 | 4.63% | 0.86% | 52.6bp (52.4bp) | POL013.1\_MED-1/Jaspar(0.638) More Information | Similar Motifs Found | motif file (matrix) |
| 35 | G A C T C A G T T A C G C T G A T A C G A T G C G T A C A T C G | 1e-42 | -9.697e+01 | 3.81% | 0.73% | 56.9bp (0.0bp) | ZBTB6/MA1581.1/Jaspar(0.867) More Information | Similar Motifs Found | motif file (matrix) |
| 36 | T A G C C A T G A T C G G T C A C T G A T G A C G A C T A T G C G A C T T A G C | 1e-41 | -9.590e+01 | 5.88% | 1.51% | 51.7bp (53.4bp) | VDR/MA0693.2/Jaspar(0.721) More Information | Similar Motifs Found | motif file (matrix) |
| 37 | C G T A A C G T A C T G A C G T A G T C A G T C A C T G C G T A | 1e-40 | -9.311e+01 | 3.72% | 0.82% | 62.4bp (24.8bp) | PB0115.1\_Ehf\_2/Jaspar(0.665) More Information | Similar Motifs Found | motif file (matrix) |
| 38 | A C T G C G T A A G T C C G T A A C T G A G T C A C G T A C T G A C T G A C T G A C T G A G T C | 1e-39 | -9.119e+01 | 3.68% | 0.61% | 53.0bp (9.8bp) | Tcf12/MA0521.1/Jaspar(0.773) More Information | Similar Motifs Found | motif file (matrix) |
| 39 | T A C G G T A C A C T G C G A T T G A C C T G A C T A G A T C G C T G A T A G C T A G C C A G T A C T G T A C G G T A C C G T A T C A G C T A G T C A G T C G A T C G A C A G T T C A G T A G C C G T A C T A G C G A T C A T G C T G A T A G C G T A C G C A T G T A C C T G A C A T G | 1e-39 | -9.119e+01 | 3.68% | 0.69% | 52.9bp (22.5bp) | PB0091.1\_Zbtb3\_1/Jaspar(0.466) More Information | Similar Motifs Found | motif file (matrix) |
| 40 | A T C G G C A T T G C A G C T A T C A G A T G C T A G C C G T A T C A G G T A C T G A C T C G A A G T C G A C T G T C A A G T C T G C A T G A C G T A C C G T A T A C G C A G T T G C A C G T A C T A G T G A C G A T C T C G A A T C G T A G C G A T C T C G A A T G C G A C T C T G A | 1e-38 | -8.929e+01 | 3.63% | 0.58% | 46.5bp (0.0bp) | PB0029.1\_Hic1\_1/Jaspar(0.395) More Information | Similar Motifs Found | motif file (matrix) |
| 41 | T C G A T G C A C G T A A C T G G T C A T C G A C G T A G T A C G T A C G T C A T G A C C G A T T C G A T G C A T C G A | 1e-38 | -8.849e+01 | 4.37% | 1.01% | 53.3bp (19.0bp) | RUNX1(Runt)/Jurkat-RUNX1-ChIP-Seq(GSE29180)/Homer(0.804) More Information | Similar Motifs Found | motif file (matrix) |
| 42 | G C A T C G T A G C A T G C T A G C A T C G A T G C A T G C T A G C T A G C A T G C T A C G A T C G A T G C T A G C T A | 1e-36 | -8.365e+01 | 3.50% | 0.82% | 57.7bp (50.7bp) | PB0129.1\_Glis2\_2/Jaspar(0.742) More Information | Similar Motifs Found | motif file (matrix) |
| 43 | C G T A A G T C C G T A A G T C A C G T A C T G C G T A A C G T A G T C A C T G A C T G A G T C | 1e-35 | -8.286e+01 | 7.66% | 2.80% | 53.8bp (42.4bp) | NFYA/MA0060.3/Jaspar(0.639) More Information | Similar Motifs Found | motif file (matrix) |
| 44 | A G T C A C T G C G T A A C T G A G T C A C G T C G T A A C T G | 1e-34 | -8.022e+01 | 4.15% | 1.02% | 55.5bp (58.5bp) | POL010.1\_DCE\_S\_III/Jaspar(0.614) More Information | Similar Motifs Found | motif file (matrix) |
| 45 | A C G T A C T G A T G C G A T C C G T A A T C G T A G C G A C T A G C T A T G C G C A T C T A G | 1e-34 | -7.995e+01 | 3.42% | 0.74% | 51.1bp (34.3bp) | OSR2/MA1646.1/Jaspar(0.757) More Information | Similar Motifs Found | motif file (matrix) |
| 46 | T G A C C G A T T A G C G T C A T G A C G T A C A T G C G T C A A T C G T G A C T G A C G T A C G T A C T G A C G T C A T G A C G T A C G T A C T G A C G T A C T G A C C T A G T A G C G T A C T G A C T G A C T G A C T G C A A T C G T G A C T G A C G T A C G T A C T G A C G T C A T A G C G T A C G T A C T G A C G T A C T A G C C T A G T A G C G T A C T G A C T G A C T G A C T G C A A T C G T G A C | 1e-33 | -7.699e+01 | 4.07% | 1.12% | 47.7bp (0.0bp) | RREB1/MA0073.1/Jaspar(0.459) More Information | Similar Motifs Found | motif file (matrix) |
| 47 | C G T A A C G T C G T A C G T A C G T A A C G T A C T G A G T C A C G T A C G T C G T A A C G T | 1e-30 | -6.918e+01 | 3.16% | 0.81% | 55.0bp (46.9bp) | MAFG/MA0659.2/Jaspar(0.652) More Information | Similar Motifs Found | motif file (matrix) |
| 48 | A C G T A G C T A C T G C G T A C G T A A C G T C T A G A C G T | 1e-30 | -6.918e+01 | 3.16% | 0.78% | 57.2bp (39.4bp) | NKX2-5/MA0063.2/Jaspar(0.811) More Information | Similar Motifs Found | motif file (matrix) |
| 49 | A T G C G C T A T A C G C G T A C G A T C T G A T A C G C T G A A G C T C G T A T C G A G C T A C A T G T G A C G C A T G A T C G T C A C T G A A T C G C G A T T A C G G C A T T A G C G C A T A G T C C G A T A T G C G A C T A C G T A G C T C G A T A C G T T A G C G C A T A G C T C A G T C T G A A G C T G A T C A G C T G C A T C G A T A G C T C G A T T A C G | 1e-29 | -6.755e+01 | 3.81% | 1.10% | 51.9bp (22.5bp) | PB0093.1\_Zfp105\_1/Jaspar(0.352) More Information | Similar Motifs Found | motif file (matrix) |
| 50 | C T A G A G T C C T A G A T C G A G C T G A T C G A C T A C T G A C T G A G C T A G T C G C T A C A G T A T C G G A T C A G C T A G C T C A G T T C A G A G T C G T A C G T C A A T G C G C A T A T C G A C G T C T A G T A G C G A C T A G T C G C T A A C T G T G A C C T G A A T C G A G C T A C T G G C T A C A T G T A C G A C T G G C A T G T A C C A G T A G C T | 1e-29 | -6.743e+01 | 3.12% | 0.69% | 48.3bp (11.5bp) | PB0050.1\_Osr1\_1/Jaspar(0.380) More Information | Similar Motifs Found | motif file (matrix) |
| 51 | C G T A A G T C A C T G A C T G A G T C A G T C A C T G A G T C | 1e-27 | -6.237e+01 | 5.37% | 1.73% | 60.6bp (39.0bp) | HINFP/MA0131.2/Jaspar(0.742) More Information | Similar Motifs Found | motif file (matrix) |
| 52 | C A T G A T C G T A G C A G T C G A T C C G A T C A T G T A C G C T G A A T C G G T A C G A C T A T C G T A G C A G T C | 1e-26 | -6.134e+01 | 2.16% | 0.27% | 53.1bp (0.0bp) | ZNF415(Zf)/HEK293-ZNF415.GFP-ChIP-Seq(GSE58341)/Homer(0.721) More Information | Similar Motifs Found | motif file (matrix) |
| 53 | G C A T G C A T G C A T G C A T G A T C G A T C G C T A G C A T C G A T G C T A G C T A G C A T G C A T G C A T G C A T | 1e-26 | -6.134e+01 | 2.16% | 0.39% | 43.3bp (13.6bp) | NFAT5/MA0606.1/Jaspar(0.686) More Information | Similar Motifs Found | motif file (matrix) |
| 54 | A C G T C G T A C G T A A C G T A G T C A C T G C G T A A G T C | 1e-24 | -5.562e+01 | 3.46% | 1.01% | 53.6bp (51.2bp) | PB0179.1\_Sp100\_2/Jaspar(0.754) More Information | Similar Motifs Found | motif file (matrix) |
| 55 | C A G T A C T G C G A T A T C G C G A T C A T G A C G T C T A G A C G T A C G T A G T C G C A T C T G A A C T G C T G A C T A G T C A G C A G T T C A G C T G A A C T G C T A G C A T G T C A G C A G T A T C G A C G T A C T G C A G T A C T G | 1e-23 | -5.524e+01 | 2.03% | 0.37% | 50.5bp (0.0bp) | ZBTB12(Zf)/HEK293-ZBTB12.GFP-ChIP-Seq(GSE58341)/Homer(0.461) More Information | Similar Motifs Found | motif file (matrix) |
| 56 | G C A T G C A T A C G T A T G C G C A T C G A T G C T A C G T A C G A T G A T C G C A T G T C A A C G T C G A T G C A T | 1e-23 | -5.398e+01 | 2.77% | 0.82% | 53.5bp (49.0bp) | PH0018.1\_Dbx1/Jaspar(0.622) More Information | Similar Motifs Found | motif file (matrix) |
| 57 | T A G C C G A T C A T G T A C G T C G A T A G C C G T A A T C G A T G C G A C T A G T C G A C T | 1e-23 | -5.359e+01 | 3.98% | 1.15% | 51.1bp (16.9bp) | ATOH1(var.2)/MA1467.1/Jaspar(0.665) More Information | Similar Motifs Found | motif file (matrix) |
| 58 | G C A T C G A T G C A T G C T A C G T A G C T A G C A T C G T A G C A T G C A T G C A T G C T A G C A T G A C T A G C T C G A T C G A T C G A T C A G T C G A T G C A T G C A T G C A T G A C T C G A T G A C T G A C T G C A T G C T A G C T A C G T A C G T A C G T A C G A T C G A T C G A T G C A T G A C T G A T C G A C T | 1e-22 | -5.293e+01 | 9.87% | 5.08% | 47.4bp (34.7bp) | ZNF384/MA1125.1/Jaspar(0.563) More Information | Similar Motifs Found | motif file (matrix) |
| 59 | A C T G A C G T C G T A C G T A A C T G C G T A A G T C A C T G | 1e-21 | -5.057e+01 | 4.41% | 1.56% | 55.2bp (48.9bp) | PROX1/MA0794.1/Jaspar(0.681) More Information | Similar Motifs Found | motif file (matrix) |
| 60 | C G T A A C T G A C G T A C T G C G T A A C G T | 1e-20 | -4.764e+01 | 8.35% | 4.04% | 53.5bp (58.3bp) | GFI1/MA0038.2/Jaspar(0.741) More Information | Similar Motifs Found | motif file (matrix) |
| 61 | A C G T G T C A A C T G C G T A C G T A A C T G | 1e-20 | -4.754e+01 | 11.12% | 6.00% | 51.8bp (52.7bp) | POL008.1\_DCE\_S\_I/Jaspar(0.826) More Information | Similar Motifs Found | motif file (matrix) |
| 62 | C T G A T G A C G C T A T C A G C G A T T C A G G T C A T G A C G T A C G C T A G T A C G T A C G A T C G T A C G A T C G T A C G T A C G C T A T G A C G C T A T C A G C G T A T C A G G T C A G T C A | 1e-20 | -4.741e+01 | 1.86% | 0.28% | 49.5bp (0.0bp) | RREB1/MA0073.1/Jaspar(0.471) More Information | Similar Motifs Found | motif file (matrix) |
| 63 | A C G T A C G T A C G T A C G T A G C T A G C T C A G T C A T G C A T G C A T G C A T G C A T G | 1e-18 | -4.177e+01 | 1.73% | 0.46% | 52.2bp (6.2bp) | PB0092.1\_Zbtb7b\_1/Jaspar(0.730) More Information | Similar Motifs Found | motif file (matrix) |
| 64 | T A G C G T A C G T C A T A G C C T A G A T C G A G T C A G T C G T A C G C A T A T C G A C G T A G T C T G A C A T G C G T C A A T G C A C G T A T G C A G T C | 1e-16 | -3.814e+01 | 1.64% | 0.55% | 50.2bp (71.7bp) | PB0114.1\_Egr1\_2/Jaspar(0.607) More Information | Similar Motifs Found | motif file (matrix) |
| 65 | G A T C A C T G A C T G A C T G C T G A A C G T A C T G C T A G A C T G A C G T A T C G C G A T A C T G T G C A A C T G C A G T C T A G A C T G C G A T A C T G G A C T A G T C G C A T T G A C C A T G A C T G A C T G C G T A C A G T A C T G A C T G C T A G A G C T T C A G C G A T A C T G T C G A A C T G A G C T C T A G A T C G A G C T A C T G A C G T A G T C | 1e-15 | -3.477e+01 | 5.15% | 2.54% | 46.8bp (44.4bp) | KLF17/MA1514.1/Jaspar(0.402) More Information | Similar Motifs Found | motif file (matrix) |
| 66 | C G T A C G T A G T C A G C A T A T C G G C A T G C T A G C A T C G A T A G T C C T G A C A G T G A C T C G A T C T A G | 1e-14 | -3.435e+01 | 2.21% | 0.63% | 58.2bp (44.7bp) | ONECUT1/MA0679.2/Jaspar(0.627) More Information | Similar Motifs Found | motif file (matrix) |
| 67 | A C T G C G T A C G T A A C G T C G T A A C G T A C T G A C G T A C G T A C G T A C G T A C G T A C T G C G T A C G T A | 1e-14 | -3.296e+01 | 2.16% | 0.65% | 46.4bp (21.9bp) | PB0116.1\_Elf3\_2/Jaspar(0.633) More Information | Similar Motifs Found | motif file (matrix) |
| 68 | C T G A C A G T C T A G C A G T C T A G C A G T C A T G C G A T A T C G C A G T A T C G C A G T C A T G A G C T C A G T | 1e-13 | -3.083e+01 | 5.37% | 2.75% | 52.3bp (40.7bp) | KLF9/MA1107.2/Jaspar(0.663) More Information | Similar Motifs Found | motif file (matrix) |
| 69 | C A G T A C G T C G A T C A G T C A G T C A G T C A G T C A G T C G T A C G T A C A T G C G T A C A T G C T A G C G A T C T A G C G T A C G A T C A G T C A G T C A G T C A G T C A G T C G A T C A G T | 1e-12 | -2.958e+01 | 2.60% | 1.02% | 52.4bp (59.4bp) | PB0116.1\_Elf3\_2/Jaspar(0.410) More Information | Similar Motifs Found | motif file (matrix) |
| 70 \* | C G A T A C G T C G A T C A G T C G A T A C G T T C G A C G T A C A G T C G A T G C A T A G C T G C A T A C G T C G A T A C G T C G A T C G A T A C G T C G A T | 1e-11 | -2.629e+01 | 1.34% | 0.53% | 51.9bp (4.2bp) | PB0116.1\_Elf3\_2/Jaspar(0.461) More Information | Similar Motifs Found | motif file (matrix) |
| 71 \* | A G T C C G T A A G T C A G T C A C T G C G T A | 1e-11 | -2.601e+01 | 2.94% | 1.33% | 55.1bp (45.5bp) | ZBTB7C/MA0695.1/Jaspar(0.713) More Information | Similar Motifs Found | motif file (matrix) |
| 72 \* | G C A T A T C G G T A C C T G A A G T C T A G C G A C T A T C G T G C A T G A C G T C A C A G T T C A G T A G C G T A C G T A C C T G A T A G C G C A T A T C G A C G T A C T G A G T C T C A G T G A C | 1e-9 | -2.216e+01 | 10.99% | 7.41% | 51.6bp (42.0bp) | Klf9(Zf)/GBM-Klf9-ChIP-Seq(GSE62211)/Homer(0.550) More Information | Similar Motifs Found | motif file (matrix) |
| 73 \* | C T A G C T G A G C T A G C T A C G A T C G A T C G T A G C A T C G T A G C T A G C T A G C T A C A G T A G C T G C A T | 1e-9 | -2.147e+01 | 1.77% | 0.82% | 53.2bp (26.7bp) | Pax7(Paired,Homeobox),longest/Myoblast-Pax7-ChIP-Seq(GSE25064)/Homer(0.696) More Information | Similar Motifs Found | motif file (matrix) |
| 74 \* | C G T A C G T A C G T A A C T G A G T C A C T G | 1e-8 | -1.860e+01 | 2.60% | 1.41% | 59.4bp (23.1bp) | POL010.1\_DCE\_S\_III/Jaspar(0.674) More Information | Similar Motifs Found | motif file (matrix) |
| 75 \* | G T C A A C G T A G T C C T A G A C T G G C A T A T G C A G C T T G A C G A C T A C G T A T G C G T A C C G A T A G T C | 1e-7 | -1.690e+01 | 2.51% | 1.31% | 55.4bp (10.2bp) | SPI1/MA0080.5/Jaspar(0.697) More Information | Similar Motifs Found | motif file (matrix) |
| 76 \* | T C A G C T A G C T A G T C A G C T G A T C A G T C A G T C A G C T A G C T A G C T A G T C A G C T A G T C A G C T A G T C A G C T A G C T A G T C A G T C A G C T A G T C A G T C A G T C A G T C A G | 1e-7 | -1.658e+01 | 38.99% | 33.92% | 50.2bp (52.7bp) | EWSR1-FLI1/MA0149.1/Jaspar(0.533) More Information | Similar Motifs Found | motif file (matrix) |
| 77 \* | A T C G C A T G C A T G T A C G C A T G A T C G A C T G A T C G A C T G A T C G C T A G C T A G T A C G T C A G C A T G C T A G C T A G T A C G C A T G T A C G C A T G T A C G C T A G T A C G C A T G C A T G T A C G C A T G T A C G A C T G | 1e-2 | -6.773e+00 | 2.60% | 1.89% | 46.6bp (39.3bp) | PB0097.1\_Zfp281\_1/Jaspar(0.644) More Information | Similar Motifs Found | motif file (matrix) |
| 78 \* | C G T A A C T G C A G T C A T G C A T G C A G T C A T G A C G T C A G T C A G T C A T G C A T G C T A G C A T G C T G A C A G T C A T G C A T G C A G T C A G T C A T G C A G T C A T G C T G A A C T G C A G T C A T G C A T G C A G T C A T G A C G T C A T G C G T A C A T G C A T G C T A G C A T G C T G A C A G T C A T G A C T G C A G T C A G T C A T G C A G T C A T G C T G A A C T G C A G T C T A G | 1e0 | -2.208e+00 | 1.43% | 1.41% | 42.1bp (27.7bp) | RREB1/MA0073.1/Jaspar(0.436) More Information | Similar Motifs Found | motif file (matrix) |
