# Supplementary material for: Global hypo-methylation in a proportion of glioblastoma enriched for an astrocytic signature is associated with increased invasion and altered immune landscape
Source: eLife. 2022 Nov 22;11:e77335. doi: 10.7554/eLife.77335 (PMC9681209; doi:10.7554/eLife.77335)
Supplement: Figure 2—source data 1. [file elife-77335-fig2-data1.zip › Figure_2_source_data_1/Figure_2B/knownResults.html]

/data/Blizard-MarinoLab/Nicola\_Pomella/Motifs\_Sara/JamesAnalysis\_Size\_200\_Motifs\_6to50/ - Homer Known Motif Enrichment Results


# Homer Known Motif Enrichment Results (/data/Blizard-MarinoLab/Nicola\_Pomella/Motifs\_Sara/JamesAnalysis\_Size\_200\_Motifs\_6to50/)

Homer *de novo* Motif Results  
Gene Ontology Enrichment Results  
Known Motif Enrichment Results (txt file)  
Total Target Sequences = 2308, Total Background Sequences = 357

|  |  |  |  |  |  |  |  |  |  |  |  |
| --- | --- | --- | --- | --- | --- | --- | --- | --- | --- | --- | --- |
| Rank | Motif | Name | P-value | log P-pvalue | q-value (Benjamini) | # Target Sequences with Motif | % of Targets Sequences with Motif | # Background Sequences with Motif | % of Background Sequences with Motif | Motif File | SVG |
| 1 | T A C G A T G C G A C T A C T G A G C T A G T C G T C A T G C A A C G T A G T C G C T A T G C A | Pknox1(Homeobox)/ES-Prep1-ChIP-Seq(GSE63282)/Homer | 1e-22 | -5.128e+01 | 0.0000 | 45.0 | 1.95% | 1.0 | 0.28% | motif file (matrix) | svg |
| 2 | T C G A C A T G C A T G A C G T A T G C T C G A C T G A A G C T T A C G T G C A G T A C G A T C A G C T A G T C | FXR(NR),IR1/Liver-FXR-ChIP-Seq(Chong\_et\_al.)/Homer | 1e-20 | -4.824e+01 | 0.0000 | 100.0 | 4.33% | 5.9 | 1.67% | motif file (matrix) | svg |
| 3 | T A G C T A G C G A C T C T A G A G C T A G T C G T C A T G C A A C G T A T G C G C T A T G C A | Pbx3(Homeobox)/GM12878-PBX3-ChIP-Seq(GSE32465)/Homer | 1e-18 | -4.363e+01 | 0.0000 | 41.0 | 1.77% | 2.0 | 0.56% | motif file (matrix) | svg |
| 4 | T G A C G T A C C G T A A C T G T G A C C G A T A C T G A T C G A G C T T A C G T C G A T A G C G T A C C G T A A T C G T G A C G C A T A C T G A C T G A T G C | Twist(bHLH)/HMLE-TWIST1-ChIP-Seq(Chang\_et\_al)/Homer | 1e-18 | -4.363e+01 | 0.0000 | 41.0 | 1.77% | 0.9 | 0.25% | motif file (matrix) | svg |
| 5 | T G C A C T G A C A T G C T A G C A G T A G T C C G T A A T G C A T G C T A C G G C A T T C A G G T C A G A T C G T A C | ERE(NR),IR3/MCF7-ERa-ChIP-Seq(Unpublished)/Homer | 1e-13 | -3.160e+01 | 0.0000 | 49.0 | 2.12% | 2.3 | 0.65% | motif file (matrix) | svg |
| 6 | A T G C T A G C A G C T A G C T T G A C G A C T T C A G T A C G G T C A C T G A A T C G T A G C G A C T C A G T A G T C A G C T T C G A A T C G T G C A T G C A | HRE(HSF)/HepG2-HSF1-ChIP-Seq(GSE31477)/Homer | 1e-12 | -2.790e+01 | 0.0000 | 32.0 | 1.38% | 1.4 | 0.39% | motif file (matrix) | svg |
| 7 | T A G C C G A T T A C G A C T G A G T C A C T G A T C G A T C G C G T A C T G A | E2F1(E2F)/Hela-E2F1-ChIP-Seq(GSE22478)/Homer | 1e-11 | -2.629e+01 | 0.0000 | 31.0 | 1.34% | 0.9 | 0.27% | motif file (matrix) | svg |
| 8 | A T G C T C A G T C G A G C A T A C T G C G T A A G T C T C A G G A C T T G A C C G T A A G C T | Atf2(bZIP)/3T3L1-Atf2-ChIP-Seq(GSE56872)/Homer | 1e-11 | -2.621e+01 | 0.0000 | 57.0 | 2.47% | 3.6 | 1.01% | motif file (matrix) | svg |
| 9 | G C A T T C A G C T G A A T C G A C T G C G A T G A T C C T G A | THRb(NR)/Liver-NR1A2-ChIP-Seq(GSE52613)/Homer | 1e-10 | -2.531e+01 | 0.0000 | 831.0 | 35.96% | 104.4 | 29.55% | motif file (matrix) | svg |
| 10 | G C A T A T C G C A T G G T A C G C T A A G T C T C A G T G A C G T C A T G C A | Arnt:Ahr(bHLH)/MCF7-Arnt-ChIP-Seq(Lo\_et\_al.)/Homer | 1e-10 | -2.473e+01 | 0.0000 | 97.0 | 4.20% | 7.9 | 2.23% | motif file (matrix) | svg |
| 11 | T G A C C T A G A C T G T A G C C G A T A C T G A T G C C A T G A T C G A T C G A T C G T A G C C T G A T A G C G C T A A C T G C G T A A G C T C G T A C T G A | GATA:SCL(Zf,bHLH)/Ter119-SCL-ChIP-Seq(GSE18720)/Homer | 1e-10 | -2.318e+01 | 0.0000 | 29.0 | 1.25% | 1.2 | 0.33% | motif file (matrix) | svg |
| 12 | A G T C G A T C A G T C C G T A A T C G C A G T A G T C G T A C C T G A A C T G T C A G A G C T A G C T A G C T A G C T | PRDM15(Zf)/ESC-Prdm15-ChIP-Seq(GSE73694)/Homer | 1e-9 | -2.128e+01 | 0.0000 | 196.0 | 8.48% | 19.5 | 5.53% | motif file (matrix) | svg |
| 13 | C G T A C T G A C G T A C T A G T C G A C T A G A C T G C G T A C G T A T A C G A G C T A T C G | SpiB(ETS)/OCILY3-SPIB-ChIP-Seq(GSE56857)/Homer | 1e-9 | -2.094e+01 | 0.0000 | 52.0 | 2.25% | 3.4 | 0.95% | motif file (matrix) | svg |
| 14 | G A C T A G T C C G A T A C T G C T G A T G A C G T A C C G T A A T C G G C A T C T G A C T A G | Bcl11a(Zf)/HSPC-BCL11A-ChIP-Seq(GSE104676)/Homer | 1e-8 | -2.046e+01 | 0.0000 | 153.0 | 6.62% | 14.3 | 4.04% | motif file (matrix) | svg |
| 15 | A C T G C G T A A C T G A T G C T G A C G A T C A T C G T G C A A C T G A G T C | ZNF519(Zf)/HEK293-ZNF519.GFP-ChIP-Seq(GSE58341)/Homer | 1e-8 | -2.030e+01 | 0.0000 | 40.0 | 1.73% | 2.5 | 0.71% | motif file (matrix) | svg |
| 16 | C T G A C T G A C T A G T C G A C G T A A T G C C G T A A C T G C G T A A C G T C T G A C G A T A G C T C G T A A C G T A G T C C G A T T A C G G T C A G C A T | GATA(Zf),IR3/iTreg-Gata3-ChIP-Seq(GSE20898)/Homer | 1e-8 | -2.020e+01 | 0.0000 | 27.0 | 1.17% | 0.7 | 0.21% | motif file (matrix) | svg |
| 17 | T A G C G T A C A G T C G T A C C G A T A G T C A G T C A G T C A G T C A G T C C G T A G A T C | Zfp281(Zf)/ES-Zfp281-ChIP-Seq(GSE81042)/Homer | 1e-8 | -2.020e+01 | 0.0000 | 27.0 | 1.17% | 1.1 | 0.32% | motif file (matrix) | svg |
| 18 | C T A G A C T G T G C A A G T C C G T A A C T G A C T G A C G T C T A G C G A T T A C G A G T C | ZEB2(Zf)/SNU398-ZEB2-ChIP-Seq(GSE103048)/Homer | 1e-7 | -1.799e+01 | 0.0000 | 252.0 | 10.90% | 27.0 | 7.65% | motif file (matrix) | svg |
| 19 | T C G A G C A T A C T G C T G A A G T C T C A G G A C T G T A C C G T A A G C T A G T C G A T C | c-Jun-CRE(bZIP)/K562-cJun-ChIP-Seq(GSE31477)/Homer | 1e-7 | -1.614e+01 | 0.0000 | 47.0 | 2.03% | 3.2 | 0.91% | motif file (matrix) | svg |
| 20 | T A C G A C T G A G C T G T A C C G T A T C G A C T G A A C T G C A T G A C G T A G T C C G T A | COUP-TFII(NR)/K562-NR2F1-ChIP-Seq(Encode)/Homer | 1e-6 | -1.606e+01 | 0.0000 | 285.0 | 12.33% | 32.1 | 9.07% | motif file (matrix) | svg |
| 21 | C A T G A G T C C T G A T G A C A T C G G A C T G T C A A G T C T A G C G A T C | HIF2a(bHLH)/785\_O-HIF2a-ChIP-Seq(GSE34871)/Homer | 1e-6 | -1.410e+01 | 0.0000 | 73.0 | 3.16% | 6.4 | 1.82% | motif file (matrix) | svg |
| 22 | T G C A G T A C C G T A A T C G A C T G A C G T C T A G C G A T T C G A A G T C | ZEB1(Zf)/PDAC-ZEB1-ChIP-Seq(GSE64557)/Homer | 1e-5 | -1.281e+01 | 0.0001 | 442.0 | 19.13% | 55.9 | 15.81% | motif file (matrix) | svg |
| 23 | T G C A C G T A A C T G T C A G C A G T C A T G T C A G G A T C T A C G A G T C T G C A A C T G A C T G T G A C G T C A | ZNF165(Zf)/WHIM12-ZNF165-ChIP-Seq(GSE65937)/Homer | 1e-5 | -1.193e+01 | 0.0001 | 32.0 | 1.38% | 2.7 | 0.76% | motif file (matrix) | svg |
| 24 | T C G A T C A G T C G A A C T G C A T G A C G T A G T C C T G A | COUP-TFII(NR)/Artia-Nr2f2-ChIP-Seq(GSE46497)/Homer | 1e-5 | -1.186e+01 | 0.0001 | 338.0 | 14.63% | 41.7 | 11.80% | motif file (matrix) | svg |
| 25 | T G C A G C A T C G A T C G T A C A G T A C T G G T A C C G T A C T G A A G C T G T C A A C T G C T A G G T C A C G A T A C T G G T A C T G C A C G T A A G C T | CEBP:CEBP(bZIP)/MEF-Chop-ChIP-Seq(GSE35681)/Homer | 1e-4 | -1.098e+01 | 0.0003 | 20.0 | 0.87% | 2.0 | 0.56% | motif file (matrix) | svg |
| 26 | T A G C G T A C C G T A C T A G A C T G T G C A C G T A A T G C C G T A A T C G | AR-halfsite(NR)/LNCaP-AR-ChIP-Seq(GSE27824)/Homer | 1e-4 | -1.035e+01 | 0.0005 | 797.0 | 34.49% | 108.1 | 30.58% | motif file (matrix) | svg |
| 27 | A T G C G A T C C G A T C T A G A C T G G C T A C G T A A G C T A C T G A G C T | TEAD2(TEA)/Py2T-Tead2-ChIP-Seq(GSE55709)/Homer | 1e-4 | -1.031e+01 | 0.0005 | 100.0 | 4.33% | 10.3 | 2.91% | motif file (matrix) | svg |
| 28 | A C T G C A G T A C G T C G T A C G T A A C G T A C T G C T G A | Nkx6.1(Homeobox)/Islet-Nkx6.1-ChIP-Seq(GSE40975)/Homer | 1e-4 | -1.006e+01 | 0.0007 | 577.0 | 24.97% | 76.2 | 21.57% | motif file (matrix) | svg |
| 29 | T A G C C T A G T C G A G A C T A C T G C T G A A G T C T C A G G C A T T G A C C T G A A G C T | Atf7(bZIP)/3T3L1-Atf7-ChIP-Seq(GSE56872)/Homer | 1e-4 | -9.982e+00 | 0.0007 | 83.0 | 3.59% | 8.2 | 2.33% | motif file (matrix) | svg |
| 30 | A T G C G A T C C G T A A G C T C A G T A T C G G C A T A G C T G A C T A C T G | Sox17(HMG)/Endoderm-Sox17-ChIP-Seq(GSE61475)/Homer | 1e-4 | -9.936e+00 | 0.0007 | 123.0 | 5.32% | 13.3 | 3.78% | motif file (matrix) | svg |
| 31 | C G T A A C T G G T C A A C G T A T C G C A G T C T A G T C A G C G T A A C T G C G T A A C G T C G T A C T G A T A C G | GATA3(Zf),DR4/iTreg-Gata3-ChIP-Seq(GSE20898)/Homer | 1e-4 | -9.832e+00 | 0.0008 | 19.0 | 0.82% | 0.0 | 0.00% | motif file (matrix) | svg |
| 32 | G T C A C T G A T C A G G A T C T G C A T G C A A C G T T C A G C G T A C G T A C G T A G C T A | Hoxd12(Homeobox)/ChickenMSG-Hoxd12.Flag-ChIP-Seq(GSE86088)/Homer | 1e-4 | -9.729e+00 | 0.0008 | 401.0 | 17.35% | 51.8 | 14.67% | motif file (matrix) | svg |
| 33 | G A C T C T A G C T A G A G T C T G C A A C T G A C G T A C G T C T A G T C A G | AMYB(HTH)/Testes-AMYB-ChIP-Seq(GSE44588)/Homer | 1e-4 | -9.675e+00 | 0.0008 | 272.0 | 11.77% | 33.9 | 9.58% | motif file (matrix) | svg |
| 34 | G A T C G C T A C A G T A C G T T A C G A G T C A T G C C T A G A G T C T C G A | Zfp57(Zf)/H1-ZFP57.HA-ChIP-Seq(GSE115387)/Homer | 1e-4 | -9.615e+00 | 0.0009 | 57.0 | 2.47% | 5.2 | 1.47% | motif file (matrix) | svg |
| 35 | C T G A C T A G T C G A C G T A A T G C C G T A A T C G C G A T T A G C G C A T A T C G G C A T A G C T G A T C G A C T A G C T | ARE(NR)/LNCAP-AR-ChIP-Seq(GSE27824)/Homer | 1e-4 | -9.572e+00 | 0.0009 | 39.0 | 1.69% | 3.4 | 0.96% | motif file (matrix) | svg |
| 36 | A C G T G A C T A T G C G C T A C T G A C T A G A C T G G A C T A G T C C G T A | Nr5a2(NR)/mES-Nr5a2-ChIP-Seq(GSE19019)/Homer | 1e-4 | -9.407e+00 | 0.0010 | 98.0 | 4.24% | 10.9 | 3.07% | motif file (matrix) | svg |
| 37 | C A T G G A C T T A C G G T C A G T A C G A T C G A C T A G C T A T C G T C G A T A C G T A G C | ERRg(NR)/Kidney-ESRRG-ChIP-Seq(GSE104905)/Homer | 1e-3 | -9.036e+00 | 0.0014 | 159.0 | 6.88% | 18.1 | 5.13% | motif file (matrix) | svg |
| 38 | T G A C G T C A A G T C G C T A C T A G A G T C C T G A C A T G A C T G C T A G T A C G T C A G | Zic2(Zf)/ESC-Zic2-ChIP-Seq(SRP197560)/Homer | 1e-3 | -9.005e+00 | 0.0014 | 105.0 | 4.54% | 11.7 | 3.30% | motif file (matrix) | svg |
| 39 | C T G A T C A G C T G A C T A G C A T G A C G T A T G C C G T A A T G C G C A T T C A G C T G A A C T G A C G T C A G T A G T C C G T A C A G T C T A G C A T G | VDR(NR),DR3/GM10855-VDR+vitD-ChIP-Seq(GSE22484)/Homer | 1e-3 | -8.856e+00 | 0.0016 | 38.0 | 1.64% | 3.7 | 1.03% | motif file (matrix) | svg |
| 40 | T A C G T A C G G T A C A T C G T A C G T A C G G T C A C T G A C G T A G A C T | E2F4(E2F)/K562-E2F4-ChIP-Seq(GSE31477)/Homer | 1e-3 | -8.854e+00 | 0.0016 | 47.0 | 2.03% | 4.9 | 1.39% | motif file (matrix) | svg |
| 41 | C T A G C A T G C A T G T A C G A G T C G C A T A G C T C T A G A C G T A G T C G A C T A C T G A C T G A C T G T C G A | Zfp809(Zf)/ES-Zfp809-ChIP-Seq(GSE70799)/Homer | 1e-3 | -8.762e+00 | 0.0017 | 64.0 | 2.77% | 6.2 | 1.77% | motif file (matrix) | svg |
| 42 | T C G A A C T G C A T G A G C T A G T C C G T A C T G A C T A G A C T G C G A T A T G C C T G A | RAR:RXR(NR),DR0/ES-RAR-ChIP-Seq(GSE56893)/Homer | 1e-3 | -8.738e+00 | 0.0017 | 18.0 | 0.78% | 1.3 | 0.37% | motif file (matrix) | svg |
| 43 | C G T A C G T A C G T A A G T C A G C T C T G A A C T G A C T G A G C T A G T C C G T A C T A G C T A G C T A G T G C A | RORa(NR)/Liver-Rora-ChIP-Seq(GSE101115)/Homer | 1e-3 | -8.738e+00 | 0.0017 | 18.0 | 0.78% | 1.4 | 0.40% | motif file (matrix) | svg |
| 44 | T G C A C T A G C T A G C T G A C A T G A C T G T G C A G A T C G T C A T G C A G T C A G T C A A G C T C T A G G C A T | ZNF675(Zf)/HEK293-ZNF675.GFP-ChIP-Seq(GSE58341)/Homer | 1e-3 | -8.738e+00 | 0.0017 | 18.0 | 0.78% | 0.8 | 0.21% | motif file (matrix) | svg |
| 45 | C T A G A C T G C T A G T C A G T C A G T A C G C T A G A C T G | Maz(Zf)/HepG2-Maz-ChIP-Seq(GSE31477)/Homer | 1e-3 | -8.576e+00 | 0.0018 | 318.0 | 13.76% | 40.7 | 11.51% | motif file (matrix) | svg |
| 46 | C T A G T A C G A G T C C G T A A G T C A C G T A G T C T C G A C G T A T A C G | Nkx2.1(Homeobox)/LungAC-Nkx2.1-ChIP-Seq(GSE43252)/Homer | 1e-3 | -7.962e+00 | 0.0033 | 511.0 | 22.11% | 68.5 | 19.40% | motif file (matrix) | svg |
| 47 | T G C A T C G A T C G A A C G T C A T G C G T A A G T C T C A G A C G T G T A C C G T A A G C T | CREB5(bZIP)/LNCaP-CREB5.V5-ChIP-Seq(GSE137775)/Homer | 1e-3 | -7.737e+00 | 0.0041 | 62.0 | 2.68% | 6.9 | 1.95% | motif file (matrix) | svg |
| 48 | A T G C C G T A A C T G C G T A A C G T G C T A T C G A A G C T C G A T C G T A A C G T A G T C C G A T A C T G G A T C | GATA(Zf),IR4/iTreg-Gata3-ChIP-Seq(GSE20898)/Homer | 1e-3 | -7.695e+00 | 0.0041 | 17.0 | 0.74% | 0.7 | 0.20% | motif file (matrix) | svg |
| 49 | G C T A C G T A A G T C A C G T T C G A T A C G A C T G A G C T A G T C T C G A | RORgt(NR)/EL4-RORgt.Flag-ChIP-Seq(GSE56019)/Homer | 1e-3 | -7.695e+00 | 0.0041 | 17.0 | 0.74% | 1.4 | 0.40% | motif file (matrix) | svg |
| 50 | G C T A C G T A A G T C A C G T T C G A T A C G A C T G A G C T A G T C T C G A | RORgt(NR)/EL4-RORgt.Flag-ChIP-Seq(GSE56019)/Homer | 1e-3 | -7.695e+00 | 0.0041 | 17.0 | 0.74% | 1.4 | 0.40% | motif file (matrix) | svg |
| 51 | T C G A G C A T A T G C C T G A A T G C T A G C A G T C G T A C T C G A A G C T | Srebp1a(bHLH)/HepG2-Srebp1a-ChIP-Seq(GSE31477)/Homer | 1e-3 | -7.641e+00 | 0.0041 | 45.0 | 1.95% | 4.8 | 1.35% | motif file (matrix) | svg |
| 52 | A T G C G A C T A G C T C T A G C G T A C T A G C G A T C T A G A T C G G A T C | Nkx2.2(Homeobox)/NPC-Nkx2.2-ChIP-Seq(GSE61673)/Homer | 1e-3 | -7.585e+00 | 0.0043 | 356.0 | 15.40% | 46.1 | 13.05% | motif file (matrix) | svg |
| 53 | C G A T C T A G T C G A A G C T C G A T C T G A C G T A A G C T A C T G C T A G A T G C G A T C | Hoxb4(Homeobox)/ES-Hoxb4-ChIP-Seq(GSE34014)/Homer | 1e-3 | -7.497e+00 | 0.0046 | 36.0 | 1.56% | 3.5 | 1.00% | motif file (matrix) | svg |
| 54 | T C A G T C A G A C G T G T A C G C T A T C A G C T G A A C T G A C T G A G C T A G T C C G T A | EAR2(NR)/K562-NR2F6-ChIP-Seq(Encode)/Homer | 1e-3 | -7.391e+00 | 0.0050 | 263.0 | 11.38% | 33.4 | 9.45% | motif file (matrix) | svg |
| 55 | G C T A A C G T C T A G G T A C G C T A G A C T C T G A G C A T C A T G G A T C | Pit1(Homeobox)/GCrat-Pit1-ChIP-Seq(GSE58009)/Homer | 1e-3 | -7.375e+00 | 0.0050 | 227.0 | 9.82% | 28.6 | 8.08% | motif file (matrix) | svg |
| 56 | C A T G A G T C A G C T C G T A C G A T C G A T G C A T G C A T C G A T C T G A C A T G T G A C | Mef2d(MADS)/Retina-Mef2d-ChIP-Seq(GSE61391)/Homer | 1e-3 | -7.066e+00 | 0.0066 | 44.0 | 1.90% | 4.5 | 1.26% | motif file (matrix) | svg |
| 57 | T G A C T A G C T C A G T C G A T C G A C G T A A G T C C G T A C G T A C G A T C T A G T A C G | Sox7(HMG)/ESC-Sox7-ChIP-Seq(GSE133899)/Homer | 1e-3 | -7.066e+00 | 0.0066 | 44.0 | 1.90% | 4.5 | 1.26% | motif file (matrix) | svg |
| 58 | A G T C G A T C G C T A C G A T A C G T T A C G G C A T C T G A G A C T A C T G A G T C G C T A C T G A T C G A C A G T | Oct4:Sox17(POU,Homeobox,HMG)/F9-Sox17-ChIP-Seq(GSE44553)/Homer | 1e-2 | -6.857e+00 | 0.0078 | 35.0 | 1.51% | 3.9 | 1.12% | motif file (matrix) | svg |
| 59 | T A C G G A C T A C T G A C T G C T A G A T G C A G T C A G T C A G T C C T G A | ZNF692(Zf)/HEK293-ZNF692.GFP-ChIP-Seq(GSE58341)/Homer | 1e-2 | -6.857e+00 | 0.0078 | 35.0 | 1.51% | 3.7 | 1.05% | motif file (matrix) | svg |
| 60 | A G T C G A C T A C T G G A T C G T A C C G T A T G A C A G T C C G A T A G C T A C G T A C G T C T A G G A C T C T G A | ZNF7(Zf)/HepG2-ZNF7.Flag-ChIP-Seq(Encode)/Homer | 1e-2 | -6.475e+00 | 0.0113 | 106.0 | 4.59% | 12.7 | 3.58% | motif file (matrix) | svg |
| 61 | C G A T C T A G A C G T A C G T A C G T C G T A A G C T C G A T A G C T C G T A C T A G T A G C | FoxD3(forkhead)/ZebrafishEmbryo-Foxd3.biotin-ChIP-seq(GSE106676)/Homer | 1e-2 | -6.281e+00 | 0.0133 | 172.0 | 7.44% | 21.1 | 5.98% | motif file (matrix) | svg |
| 62 | C A G T A C T G T C A G T G C A G C T A A T G C T C G A A T C G G T C A T G C A | ZNF189(Zf)/HEK293-ZNF189.GFP-ChIP-Seq(GSE58341)/Homer | 1e-2 | -6.281e+00 | 0.0133 | 172.0 | 7.44% | 21.3 | 6.04% | motif file (matrix) | svg |
| 63 | A G C T A G C T C A T G C T G A G T A C A G T C A G C T A G C T C A G T C T A G | RARa(NR)/K562-RARa-ChIP-Seq(Encode)/Homer | 1e-2 | -6.278e+00 | 0.0133 | 556.0 | 24.06% | 76.2 | 21.57% | motif file (matrix) | svg |
| 64 | T A C G G A C T T G A C C G T A A C G T G A T C G T C A C G T A A C G T A T G C C G T A G A C T | HOXA2(Homeobox)/mES-Hoxa2-ChIP-Seq(Donaldson\_et\_al.)/Homer | 1e-2 | -6.156e+00 | 0.0146 | 25.0 | 1.08% | 2.6 | 0.73% | motif file (matrix) | svg |
| 65 | T A G C C G A T A C G T A G C T A G C T A G T C A T G C A G T C A C T G A T G C A T G C G C T A | E2F7(E2F)/Hela-E2F7-ChIP-Seq(GSE32673)/Homer | 1e-2 | -5.774e+00 | 0.0204 | 15.0 | 0.65% | 0.9 | 0.27% | motif file (matrix) | svg |
| 66 | T G A C T C G A C T G A C T G A A T G C G A T C C T A G T A C G G A C T G A C T G A T C T C G A C T G A C T G A A T G C G A T C C T A G A T C G G A C T G A C T | Tcfcp2l1(CP2)/mES-Tcfcp2l1-ChIP-Seq(GSE11431)/Homer | 1e-2 | -5.774e+00 | 0.0204 | 15.0 | 0.65% | 0.0 | 0.00% | motif file (matrix) | svg |
| 67 | C T A G A C G T A G T C C G T A A C T G A G T C G C A T A C T G G C A T A G T C G A C T G A T C G C A T A G T C A G C T | ZNF317(Zf)/HEK293-ZNF317.GFP-ChIP-Seq(GSE58341)/Homer | 1e-2 | -5.774e+00 | 0.0204 | 15.0 | 0.65% | 0.0 | 0.00% | motif file (matrix) | svg |
| 68 | A G C T G C A T A C T G A C G T A G T C A C G T C T A G T A C G | Smad3(MAD)/NPC-Smad3-ChIP-Seq(GSE36673)/Homer | 1e-2 | -5.637e+00 | 0.0231 | 545.0 | 23.58% | 75.4 | 21.33% | motif file (matrix) | svg |
| 69 | C T G A C T G A T A G C G A T C G C T A G T A C A C G T G A T C T G C A C G T A | Nkx2.5(Homeobox)/HL1-Nkx2.5.biotin-ChIP-Seq(GSE21529)/Homer | 1e-2 | -5.626e+00 | 0.0231 | 415.0 | 17.96% | 56.7 | 16.04% | motif file (matrix) | svg |
| 70 | G T A C C T A G T C A G A G C T T A G C C G T A A T G C T A C G A G T C G T A C G T C A A G T C | Srebp2(bHLH)/HepG2-Srebp2-ChIP-Seq(GSE31477)/Homer | 1e-2 | -5.469e+00 | 0.0265 | 24.0 | 1.04% | 2.9 | 0.81% | motif file (matrix) | svg |
| 71 | A T C G G A T C G C A T C G T A G T C A A C G T A T G C A G T C | CRX(Homeobox)/Retina-Crx-ChIP-Seq(GSE20012)/Homer | 1e-2 | -5.437e+00 | 0.0270 | 496.0 | 21.46% | 68.9 | 19.49% | motif file (matrix) | svg |
| 72 | C T G A C G A T C T A G C G T A A G C T C G A T C A G T C T G A G A C T C T A G C T A G A T G C | PBX2(Homeobox)/K562-PBX2-ChIP-Seq(Encode)/Homer | 1e-2 | -5.200e+00 | 0.0337 | 182.0 | 7.88% | 23.2 | 6.57% | motif file (matrix) | svg |
| 73 | C T A G T A C G G A T C G T A C G C T A A G C T A G C T G T C A T C G A T A G C | Nanog(Homeobox)/mES-Nanog-ChIP-Seq(GSE11724)/Homer | 1e-2 | -5.135e+00 | 0.0355 | 877.0 | 37.95% | 125.0 | 35.38% | motif file (matrix) | svg |
| 74 | A G T C T G C A T C G A C T G A A C T G C A T G A C G T A T G C G T C A T A C G | Erra(NR)/HepG2-Erra-ChIP-Seq(GSE31477)/Homer | 1e-2 | -5.006e+00 | 0.0398 | 459.0 | 19.86% | 63.9 | 18.09% | motif file (matrix) | svg |
| 75 | C T G A C A G T C T G A A G T C C T A G G A C T A T C G G T A C | HIF-1b(HLH)/T47D-HIF1b-ChIP-Seq(GSE59937)/Homer | 1e-2 | -4.925e+00 | 0.0426 | 195.0 | 8.44% | 26.0 | 7.35% | motif file (matrix) | svg |
| 76 | C T A G A G C T G A C T C A T G A G T C A G T C G T C A C A G T C T A G T C A G G T A C C T G A T C G A G A T C T G A C | Rfx2(HTH)/LoVo-RFX2-ChIP-Seq(GSE49402)/Homer | 1e-2 | -4.901e+00 | 0.0426 | 14.0 | 0.61% | 1.8 | 0.51% | motif file (matrix) | svg |
| 77 | C G T A C G T A C G T A A G T C A G C T C T G A A C T G C T A G A G C T A G T C C G T A T C A G | RORg(NR)/Liver-Rorc-ChIP-Seq(GSE101115)/Homer | 1e-2 | -4.901e+00 | 0.0426 | 14.0 | 0.61% | 1.4 | 0.40% | motif file (matrix) | svg |
