# Supplementary material for: Global hypo-methylation in a proportion of glioblastoma enriched for an astrocytic signature is associated with increased invasion and altered immune landscape
Source: eLife. 2022 Nov 22;11:e77335. doi: 10.7554/eLife.77335 (PMC9681209; doi:10.7554/eLife.77335)
Supplement: Figure 2—source data 1. [file elife-77335-fig2-data1.zip › Figure_2_source_data_1/Figure_2A/homerResults.html]

/data/Blizard-MarinoLab/Nicola\_Pomella/Motifs\_James/JamesAnalysis\_Size\_200\_Motifs\_6to50/BG3// - Homer de novo Motif Results


# Homer *de novo* Motif Results (/data/Blizard-MarinoLab/Nicola\_Pomella/Motifs\_James/JamesAnalysis\_Size\_200\_Motifs\_6to50/BG3//)

Known Motif Enrichment Results  
Gene Ontology Enrichment Results  
If Homer is having trouble matching a motif to a known motif, try copy/pasting the matrix file into
STAMP  
More information on motif finding results: HOMER
| Description of Results
| Tips
  
Total target sequences = 2323  
Total background sequences = 3647  
\* - possible false positive  

|  |  |  |  |  |  |  |  |  |
| --- | --- | --- | --- | --- | --- | --- | --- | --- |
| Rank | Motif | P-value | log P-pvalue | % of Targets | % of Background | STD(Bg STD) | Best Match/Details | Motif File |
| 1 | A G T C G A C T A T G C G A T C G A C T A C T G C A T G T C A G A T G C G A C T A T G C C A G T T A C G C T A G T C A G | 1e-33 | -7.757e+01 | 1.16% | 0.04% | 44.2bp (0.0bp) | ZNF416(Zf)/HEK293-ZNF416.GFP-ChIP-Seq(GSE58341)/Homer(0.609) More Information | Similar Motifs Found | motif file (matrix) |
| 2 | A C G T A C T G T C G A A T G C C G A T T A C G G C A T C T A G A C T G A G T C A G T C G T C A | 1e-30 | -7.073e+01 | 2.67% | 0.40% | 54.3bp (49.2bp) | Plagl1/MA1615.1/Jaspar(0.713) More Information | Similar Motifs Found | motif file (matrix) |
| 3 | A C T G A T C G A G C T A G T C G A T C A G T C T C A G A G T C A G T C C A G T A G T C G T A C | 1e-27 | -6.296e+01 | 1.38% | 0.10% | 55.6bp (44.3bp) | E2F6/MA0471.2/Jaspar(0.729) More Information | Similar Motifs Found | motif file (matrix) |
| 4 | C A T G T A G C G T A C G C T A A C G T G T C A G T C A C A G T G A T C G C T A | 1e-27 | -6.276e+01 | 0.99% | 0.03% | 41.7bp (0.0bp) | DLX1(Homeobox)/BasalGanglia-Dlx1-ChIP-seq(GSE124936)/Homer(0.620) More Information | Similar Motifs Found | motif file (matrix) |
| 5 | G A C T A G T C G A C T A G T C G C T A A T G C A G T C A G T C G A C T A G C T A G T C G T A C | 1e-27 | -6.276e+01 | 0.99% | 0.02% | 53.5bp (0.0bp) | ZNF460/MA1596.1/Jaspar(0.642) More Information | Similar Motifs Found | motif file (matrix) |
| 6 | T A C G A G T C G T A C G C A T A T G C A G T C C G T A T A G C G T A C C G T A C T A G A T C G | 1e-27 | -6.257e+01 | 1.21% | 0.06% | 49.8bp (36.4bp) | Znf281/MA1630.1/Jaspar(0.672) More Information | Similar Motifs Found | motif file (matrix) |
| 7 | A T G C A G T C G T A C A G C T C A T G A G T C A G T C G A C T A T G C A G C T A T G C A T G C G T A C C G A T T A C G A T G C A G T C A T G C G A T C G A T C | 1e-27 | -6.257e+01 | 1.21% | 0.08% | 51.8bp (42.8bp) | ZNF148/MA1653.1/Jaspar(0.579) More Information | Similar Motifs Found | motif file (matrix) |
| 8 | G A T C A G T C A C G T A G C T C T A G C T A G C A T G C A T G C T A G A T G C G A C T A C G T | 1e-25 | -5.955e+01 | 1.46% | 0.12% | 53.3bp (40.3bp) | EBF3/MA1637.1/Jaspar(0.675) More Information | Similar Motifs Found | motif file (matrix) |
| 9 | A T G C T A G C C A T G C A T G T A C G G A C T A T C G G A C T A C T G A C T G A G T C A G T C G A T C G T A C G A T C | 1e-24 | -5.639e+01 | 1.12% | 0.08% | 48.5bp (7.6bp) | EKLF(Zf)/Erythrocyte-Klf1-ChIP-Seq(GSE20478)/Homer(0.707) More Information | Similar Motifs Found | motif file (matrix) |
| 10 | C T G A C A T G A C T G A T C G C G T A C T A G A G T C G C T A A T C G G A C T A C G T C A T G | 1e-24 | -5.560e+01 | 0.90% | 0.03% | 57.4bp (0.0bp) | POL013.1\_MED-1/Jaspar(0.724) More Information | Similar Motifs Found | motif file (matrix) |
| 11 | A C T G T G C A T G A C T G C A T G A C T A G C G T A C G C T A A C T G G A T C G T A C G T A C G T A C A G T C G T A C T G A C T G A C A T G C G T A C G C T A T A C G A C G T T G C A T G A C T A C G A T G C G A T C T C G A T A C G T A G C G T A C G A C T A G T C G A C T T C G A | 1e-23 | -5.336e+01 | 1.08% | 0.06% | 47.8bp (55.8bp) | Zfp281(Zf)/ES-Zfp281-ChIP-Seq(GSE81042)/Homer(0.487) More Information | Similar Motifs Found | motif file (matrix) |
| 12 | A T G C G T A C G T A C G T C A C A G T C T G A A T C G A T G C G T A C G A C T G T C A A T G C A G C T G A C T G T C A | 1e-22 | -5.210e+01 | 0.86% | 0.04% | 49.3bp (0.0bp) | PB0154.1\_Osr1\_2/Jaspar(0.525) More Information | Similar Motifs Found | motif file (matrix) |
| 13 | C A G T T A C G A T G C T C G A A C T G A G C T T A G C C A G T C A T G C A T G A T C G G T A C G T C A C A T G T A C G A T G C A T G C A T G C C G T A A T C G A T C G T A G C T A G C A T G C G C A T T A C G G C A T A T C G T A G C G T A C G A T C C T A G T A C G T A G C T C A G T A C G C A G T A T C G T C G A T C A G T A C G A T G C G T C A T A G C A G C T | 1e-22 | -5.210e+01 | 0.86% | 0.04% | 46.3bp (0.0bp) | ZNF322(Zf)/HEK293-ZNF322.GFP-ChIP-Seq(GSE58341)/Homer(0.461) More Information | Similar Motifs Found | motif file (matrix) |
| 14 | C G A T C T G A G A C T G C T A C G A T C G A T A C G T C G A T A C G T A G T C A C G T A C T G | 1e-22 | -5.182e+01 | 1.21% | 0.11% | 37.0bp (43.5bp) | PB0192.1\_Tcfap2e\_2/Jaspar(0.658) More Information | Similar Motifs Found | motif file (matrix) |
| 15 | T A G C A C T G A C T G A G C T T G C A A G T C A G T C A C G T A C T G G T A C | 1e-21 | -4.864e+01 | 0.82% | 0.03% | 34.8bp (0.0bp) | TCF4/MA0830.2/Jaspar(0.742) More Information | Similar Motifs Found | motif file (matrix) |
| 16 | G T A C C G T A A T C G C G T A A T G C A G T C A C G T A C T G A T G C A G T C G T A C A G T C | 1e-21 | -4.864e+01 | 0.82% | 0.03% | 46.7bp (0.0bp) | POL003.1\_GC-box/Jaspar(0.678) More Information | Similar Motifs Found | motif file (matrix) |
| 17 | C A G T T A C G G C T A C T G A G A C T G T A C C G T A A G T C G T A C G A C T A T G C G T A C G T C A C G T A T A C G | 1e-21 | -4.864e+01 | 0.82% | 0.05% | 55.4bp (2.8bp) | ZKSCAN5/MA1652.1/Jaspar(0.669) More Information | Similar Motifs Found | motif file (matrix) |
| 18 | T A G C A C G T G T A C C G T A C G T A A G T C A G C T A C G T G C T A A C G T | 1e-20 | -4.742e+01 | 0.99% | 0.07% | 57.9bp (34.9bp) | VDR/MA0693.2/Jaspar(0.669) More Information | Similar Motifs Found | motif file (matrix) |
| 19 | A C T G A T C G A C G T A C T G A C G T A T G C G A T C A G T C A C T G C T A G | 1e-20 | -4.645e+01 | 1.12% | 0.11% | 51.2bp (44.6bp) | AR-halfsite(NR)/LNCaP-AR-ChIP-Seq(GSE27824)/Homer(0.732) More Information | Similar Motifs Found | motif file (matrix) |
| 20 | C T A G A T C G A T C G G T A C C A T G T A C G C T A G A T C G A T G C G A T C A T G C T G C A | 1e-20 | -4.645e+01 | 1.12% | 0.10% | 52.9bp (47.4bp) | KLF5/MA0599.1/Jaspar(0.849) More Information | Similar Motifs Found | motif file (matrix) |
| 21 | C G A T C G T A G T A C A C T G G C T A C T G A A C G T A C G T G T C A C G T A A C T G A C T G | 1e-19 | -4.523e+01 | 0.78% | 0.04% | 37.2bp (32.6bp) | PH0028.1\_En1/Jaspar(0.659) More Information | Similar Motifs Found | motif file (matrix) |
| 22 | G T C A A C G T G T C A G C T A C G T A T G C A G T C A G C T A G C T A C G A T C G T A C T G A C T G A C G T A A G C T C G T A G T C A C T G A G C A T C T G A | 1e-19 | -4.523e+01 | 0.78% | 0.04% | 48.1bp (5.8bp) | PB0148.1\_Mtf1\_2/Jaspar(0.625) More Information | Similar Motifs Found | motif file (matrix) |
| 23 | C T G A C T G A T G A C C T G A T C A G C T G A T A G C T C A G T C G A T C G A T C G A C T A G A C T G A C G T A T G C | 1e-19 | -4.478e+01 | 1.42% | 0.17% | 50.7bp (77.9bp) | TCF7L1/MA1421.1/Jaspar(0.667) More Information | Similar Motifs Found | motif file (matrix) |
| 24 | T A G C G C T A A G T C G A T C T G C A T A G C G C T A A T G C G C A T A C T G A T C G A C G T T G C A C T G A T A G C | 1e-18 | -4.198e+01 | 2.20% | 0.49% | 44.8bp (46.1bp) | RUNX2(Runt)/PCa-RUNX2-ChIP-Seq(GSE33889)/Homer(0.611) More Information | Similar Motifs Found | motif file (matrix) |
| 25 | T A G C T G C A C A T G A G T C T C A G A C T G C A T G A C T G C G T A A T G C A T C G A T G C T C A G C T A G T A C G T G A C C T A G A C T G C T A G C T A G C T A G T A G C C A G T T A C G A C T G A C T G A T C G T C A G A C T G C T A G | 1e-18 | -4.188e+01 | 0.73% | 0.04% | 55.0bp (0.0bp) | PB0097.1\_Zfp281\_1/Jaspar(0.562) More Information | Similar Motifs Found | motif file (matrix) |
| 26 | A T C G T A G C G C A T A G T C G A C T C A G T T A C G A T C G C T G A A T C G A T G C G A T C | 1e-18 | -4.165e+01 | 0.90% | 0.08% | 54.0bp (50.8bp) | PB0113.1\_E2F3\_2/Jaspar(0.658) More Information | Similar Motifs Found | motif file (matrix) |
| 27 | A G T C A G T C A G T C A G C T A C T G A C T G A C T G A C T G C G T A A C T G | 1e-17 | -4.124e+01 | 1.03% | 0.10% | 48.7bp (6.3bp) | EBF1/MA0154.4/Jaspar(0.868) More Information | Similar Motifs Found | motif file (matrix) |
| 28 | T A G C G T C A T G C A C T G A T A C G C G T A C T A G T G A C G C A T A C T G | 1e-17 | -4.118e+01 | 3.96% | 1.39% | 50.4bp (62.4bp) | MyoG(bHLH)/C2C12-MyoG-ChIP-Seq(GSE36024)/Homer(0.798) More Information | Similar Motifs Found | motif file (matrix) |
| 29 | A C T G A C G T A T C G C G T A T A C G A C G T A C T G T G A C A T G C T G C A | 1e-17 | -4.030e+01 | 1.42% | 0.22% | 56.9bp (42.8bp) | PKNOX1/MA0782.2/Jaspar(0.788) More Information | Similar Motifs Found | motif file (matrix) |
| 30 | A T G C A C G T A C T G G A C T A C T G A C T G A T G C A C G T A G T C A T C G | 1e-16 | -3.883e+01 | 0.86% | 0.07% | 59.8bp (31.9bp) | RUNX1/MA0002.2/Jaspar(0.704) More Information | Similar Motifs Found | motif file (matrix) |
| 31 | T C A G C T A G C A T G A T C G C T A G A T C G G A T C T A G C T C G A T C A G T C A G T A C G T A C G T A C G C A T G T A C G A C T G T C A G T C A G A T C G A C T G A C T G T A C G T A C G C T A G T A C G A T G C T G C A A T C G C A T G A T C G T A C G T G A C A T G C A G C T G T A C G T A C T A G C C T G A A T C G | 1e-16 | -3.883e+01 | 0.86% | 0.08% | 42.6bp (16.3bp) | PB0097.1\_Zfp281\_1/Jaspar(0.594) More Information | Similar Motifs Found | motif file (matrix) |
| 32 | A T C G A G T C A C G T A G T C G T A C A T C G A T C G C G A T A C G T A C T G A C T G A C G T G A T C A C G T A T G C | 1e-16 | -3.858e+01 | 0.69% | 0.02% | 49.2bp (0.0bp) | PB0196.1\_Zbtb7b\_2/Jaspar(0.614) More Information | Similar Motifs Found | motif file (matrix) |
| 33 | A G C T A T G C A C G T A C T G T C G A C T A G A G T C T A C G G T A C A T G C G T A C A G T C G C T A C G A T T C A G A T G C G T A C G T A C C A G T A G T C | 1e-16 | -3.858e+01 | 0.69% | 0.02% | 53.3bp (0.0bp) | KLF11/MA1512.1/Jaspar(0.480) More Information | Similar Motifs Found | motif file (matrix) |
| 34 | C T A G A T C G C G T A C G A T T A C G A T G C C G T A A T G C A T G C A T C G | 1e-16 | -3.724e+01 | 1.08% | 0.13% | 61.6bp (1.6bp) | ETS:E-box(ETS,bHLH)/HPC7-Scl-ChIP-Seq(GSE22178)/Homer(0.665) More Information | Similar Motifs Found | motif file (matrix) |
| 35 | C A T G C G A T G C T A T A G C G A T C C T G A T C G A A T C G C G A T A T C G A G C T C G A T T A C G A C G T A C G T | 1e-15 | -3.606e+01 | 0.82% | 0.08% | 47.0bp (67.4bp) | PB0122.1\_Foxk1\_2/Jaspar(0.640) More Information | Similar Motifs Found | motif file (matrix) |
| 36 | G T A C T G C A A T G C G T A C C G T A A C T G C G A T C T A G C G T A T G C A A T G C G T A C G T A C C T A G G T C A | 1e-15 | -3.534e+01 | 0.65% | 0.03% | 55.1bp (0.0bp) | VDR/MA0693.2/Jaspar(0.583) More Information | Similar Motifs Found | motif file (matrix) |
| 37 | A C G T A G T C A G T C A C T G C G T A C T A G A G T C A C T G | 1e-14 | -3.373e+01 | 0.90% | 0.10% | 44.1bp (47.9bp) | PB0115.1\_Ehf\_2/Jaspar(0.637) More Information | Similar Motifs Found | motif file (matrix) |
| 38 | C A G T A C G T A C G T A C G T A G T C A T G C G T A C C G A T C A G T G C T A G T C A A G C T | 1e-14 | -3.335e+01 | 0.78% | 0.07% | 55.8bp (22.8bp) | NFAT5/MA0606.1/Jaspar(0.711) More Information | Similar Motifs Found | motif file (matrix) |
| 39 | T C A G C T G A C A T G C A T G T G A C A T C G C A G T T A G C C T G A T A C G A T C G T C G A T A G C A G T C A C G T T A C G C A T G T A G C C G T A A T C G T A C G A T C G T C G A T C G A A C G T T A C G T A G C C G T A A C T G C G A T T C A G G C T A A G T C T G A C G C A T | 1e-14 | -3.335e+01 | 0.78% | 0.07% | 44.5bp (15.2bp) | Zfp809(Zf)/ES-Zfp809-ChIP-Seq(GSE70799)/Homer(0.471) More Information | Similar Motifs Found | motif file (matrix) |
| 40 | T G A C A T C G C G A T A C T G A T C G C G T A A T C G C A T G T A C G A C G T A C G T A C T G A C T G A C T G G T A C A T C G C A T G T C A G T A C G C G T A A T C G A C T G A C T G C T G A T A C G A C G T A C T G A T C G A C T G A T C G C G T A A T C G A G C T T C G A C T A G T C A G A T C G G C T A G C A T C T A G C T A G A C T G A C T G C A T G C A G T A C T G T C A G T C A G C A T G A C T G | 1e-13 | -3.217e+01 | 0.60% | 0.03% | 40.3bp (0.0bp) | KLF9/MA1107.2/Jaspar(0.406) More Information | Similar Motifs Found | motif file (matrix) |
| 41 | C T A G A T C G G T A C A T G C G T A C A G T C T G C A C T A G G T C A A T C G T A C G G A T C A G T C A T G C C G T A A C T G A T C G G T A C T A C G T C G A T A C G A G T C C A G T A T G C A G T C G T A C G A T C G T A C T G A C C T G A A T G C T G A C T G A C G A T C A G C T T G C A A G T C T A G C G T C A T G A C A C T G G T A C G C A T G A T C A G T C T A G C T A G C T G C A A T C G T A G C | 1e-13 | -3.217e+01 | 0.60% | 0.03% | 46.4bp (0.0bp) | Znf281/MA1630.1/Jaspar(0.394) More Information | Similar Motifs Found | motif file (matrix) |
| 42 | A C T G A G T C A C T G A G T C A T G C A C G T A C T G C G T A | 1e-13 | -3.217e+01 | 0.60% | 0.05% | 53.2bp (25.6bp) | TCFL5/MA0632.2/Jaspar(0.729) More Information | Similar Motifs Found | motif file (matrix) |
| 43 | A C G T A C T G A T C G G T A C C G T A T C G A C T A G A C T G A T G C A G T C A C G T A C T G | 1e-13 | -3.132e+01 | 0.86% | 0.10% | 47.2bp (49.0bp) | Zfx/MA0146.2/Jaspar(0.683) More Information | Similar Motifs Found | motif file (matrix) |
| 44 | T A G C C G A T A C T G A C G T T A G C G T C A T A G C C T A G T C A G T C G A T C G A C A G T C A T G C A T G T C A G | 1e-13 | -3.069e+01 | 0.73% | 0.08% | 56.0bp (28.0bp) | Pax2/MA0067.1/Jaspar(0.611) More Information | Similar Motifs Found | motif file (matrix) |
| 45 | C A T G T A C G T A C G G A C T T A G C G T A C G T C A C T G A A T G C A T C G G T A C G C T A T C G A G T C A A G T C | 1e-13 | -3.069e+01 | 0.73% | 0.06% | 57.8bp (49.3bp) | NF1:FOXA1(CTF,Forkhead)/LNCAP-FOXA1-ChIP-Seq(GSE27824)/Homer(0.607) More Information | Similar Motifs Found | motif file (matrix) |
| 46 | A T G C A G C T G C A T A T C G C T A G T A G C G C A T G T C A | 1e-13 | -3.067e+01 | 16.37% | 11.20% | 56.1bp (55.6bp) | NFIC/MA0161.2/Jaspar(0.803) More Information | Similar Motifs Found | motif file (matrix) |
| 47 | A C G T A G T C G T A C A C G T A G T C C G T A C G A T A G T C | 1e-13 | -3.057e+01 | 3.75% | 1.51% | 52.1bp (54.1bp) | GATA4/MA0482.2/Jaspar(0.670) More Information | Similar Motifs Found | motif file (matrix) |
| 48 | C G T A G T C A G T A C C G A T C G A T A C G T A G C T T G A C G A T C G C A T A C G T A C G T A G C T C G A T A C G T | 1e-12 | -2.907e+01 | 0.56% | 0.02% | 67.0bp (0.0bp) | PRDM1(Zf)/Hela-PRDM1-ChIP-Seq(GSE31477)/Homer(0.650) More Information | Similar Motifs Found | motif file (matrix) |
| 49 | A C G T T A G C A G T C A C T G A C T G T C A G C A G T A G T C A C G T T A C G | 1e-12 | -2.865e+01 | 1.16% | 0.21% | 51.2bp (65.2bp) | SPDEF/MA0686.1/Jaspar(0.666) More Information | Similar Motifs Found | motif file (matrix) |
| 50 | G T A C A G T C A C T G A C T G C G T A C G T A A G T C A C G T | 1e-12 | -2.865e+01 | 1.16% | 0.19% | 47.8bp (39.2bp) | Elk1(ETS)/Hela-Elk1-ChIP-Seq(GSE31477)/Homer(0.855) More Information | Similar Motifs Found | motif file (matrix) |
| 51 | T G C A G A C T A T G C A G T C T G A C G C T A A T G C T C A G T A C G T G C A | 1e-12 | -2.829e+01 | 0.90% | 0.13% | 55.0bp (54.9bp) | Rbpj1(?)/Panc1-Rbpj1-ChIP-Seq(GSE47459)/Homer(0.715) More Information | Similar Motifs Found | motif file (matrix) |
| 52 | C A G T C A T G A C G T A C T G A C G T A C T G A C G T A C T G A C G T A C G T A G T C A G C T G C A T A C T G C T G A C T A G T C G A A C G T C T A G C G T A | 1e-12 | -2.829e+01 | 0.90% | 0.12% | 42.1bp (11.4bp) | PB0016.1\_Foxj1\_1/Jaspar(0.532) More Information | Similar Motifs Found | motif file (matrix) |
| 53 | T A C G T C G A C A T G G A T C G A T C T G A C C T A G A G C T C T G A A C T G | 1e-12 | -2.808e+01 | 0.69% | 0.08% | 50.6bp (59.2bp) | POL010.1\_DCE\_S\_III/Jaspar(0.657) More Information | Similar Motifs Found | motif file (matrix) |
| 54 | A C T G A C T G A G T C A C T G A C T G A G C T A C G T A G T C C G T A G T A C | 1e-12 | -2.808e+01 | 0.69% | 0.08% | 53.9bp (16.2bp) | ZNF341/MA1655.1/Jaspar(0.680) More Information | Similar Motifs Found | motif file (matrix) |
| 55 | A C G T A T G C C G A T A G T C A C T G A C T G A C T G A C G T A C G T A C G T A G T C G T C A A C G T A G T C A C T G | 1e-12 | -2.808e+01 | 0.69% | 0.08% | 47.9bp (9.8bp) | SIX2/MA1119.1/Jaspar(0.607) More Information | Similar Motifs Found | motif file (matrix) |
| 56 | C A G T A C T G T G A C A G T C G T A C T A C G C A T G A G T C A G T C A G T C G T A C A G T C T G A C A T C G A G T C C T G A C T A G A C G T A T C G T G C A | 1e-12 | -2.808e+01 | 0.69% | 0.07% | 51.9bp (12.6bp) | KLF16/MA0741.1/Jaspar(0.652) More Information | Similar Motifs Found | motif file (matrix) |
| 57 \* | A G C T C T G A C T A G C T A G T A C G T C G A C A G T T A G C G A T C G A T C | 1e-11 | -2.686e+01 | 14.34% | 9.81% | 54.8bp (61.1bp) | PB0052.1\_Plagl1\_1/Jaspar(0.651) More Information | Similar Motifs Found | motif file (matrix) |
| 58 \* | G C A T A C T G A C T G C T G A A C T G A C T G C T A G A G T C C G T A A T C G C G A T A C T G A T G C A G C T A C T G | 1e-11 | -2.605e+01 | 0.52% | 0.04% | 52.4bp (0.0bp) | PB0091.1\_Zbtb3\_1/Jaspar(0.647) More Information | Similar Motifs Found | motif file (matrix) |
| 59 \* | G A C T A C G T T A G C G T C A C G A T A C T G A C T G A G T C A C G T A C G T A C G T A C G T A C G T A C G T A G C T | 1e-11 | -2.605e+01 | 0.52% | 0.05% | 66.1bp (33.9bp) | ZNF384/MA1125.1/Jaspar(0.718) More Information | Similar Motifs Found | motif file (matrix) |
| 60 \* | C G A T T A C G G T A C G T A C A G T C T G A C G A C T A T C G A T G C T G C A A T G C G T C A T G A C T G A C A G T C T A C G T A G C G T A C G T A C A T G C | 1e-11 | -2.605e+01 | 0.52% | 0.00% | 33.8bp (0.0bp) | RREB1/MA0073.1/Jaspar(0.630) More Information | Similar Motifs Found | motif file (matrix) |
| 61 \* | T C A G T C G A A G T C G T C A A G T C T G A C A T G C C T G A A T G C A G T C G A T C T A G C G A C T T A C G T G C A A C G T A T C G A G T C T G A C A G C T A G C T G A T C G T A C G A T C T G C A T A G C C G A T A T C G G T A C A T G C | 1e-11 | -2.605e+01 | 0.52% | 0.00% | 49.1bp (0.0bp) | KLF4/MA0039.4/Jaspar(0.524) More Information | Similar Motifs Found | motif file (matrix) |
| 62 \* | A T C G A C G T A C T G A G T C A T C G A C G T C G T A C T G A | 1e-11 | -2.554e+01 | 0.65% | 0.07% | 54.8bp (26.3bp) | CEBPG/MA0838.1/Jaspar(0.689) More Information | Similar Motifs Found | motif file (matrix) |
| 63 \* | T C A G A C T G A C G T C G T A A G T C A G T C C G T A A G T C | 1e-10 | -2.468e+01 | 1.21% | 0.27% | 48.1bp (66.4bp) | PB0156.1\_Plagl1\_2/Jaspar(0.735) More Information | Similar Motifs Found | motif file (matrix) |
| 64 \* | A C T G A G T C A C G T A C T G C G T A A C G T A G T C A C G T | 1e-10 | -2.396e+01 | 1.12% | 0.23% | 51.6bp (68.6bp) | GATA1/MA0035.4/Jaspar(0.740) More Information | Similar Motifs Found | motif file (matrix) |
| 65 \* | C G A T C G T A G T C A A G T C A C T G C G T A A C G T C G T A | 1e-10 | -2.318e+01 | 1.29% | 0.30% | 63.2bp (56.5bp) | SD0003.1\_at\_AC\_acceptor/Jaspar(0.717) More Information | Similar Motifs Found | motif file (matrix) |
| 66 \* | G C A T C A G T C G T A A C G T C G A T C A T G C G T A C T A G C T A G C G T A C T A G C G T A C A T G C T A G C A T G C T A G A C G T T C A G A C G T T A C G C G A T T A C G C A G T C A T G A C G T | 1e-10 | -2.310e+01 | 0.47% | 0.02% | 46.3bp (0.0bp) | KLF9/MA1107.2/Jaspar(0.580) More Information | Similar Motifs Found | motif file (matrix) |
| 67 \* | A T G C A G T C A G T C A G T C A C T G C G T A C G T A C G T A | 1e-10 | -2.306e+01 | 0.60% | 0.08% | 47.7bp (37.7bp) | PB0092.1\_Zbtb7b\_1/Jaspar(0.705) More Information | Similar Motifs Found | motif file (matrix) |
| 68 \* | C G T A A G T C A C T G A G T C A G T C C G T A A C T G A C G T | 1e-10 | -2.306e+01 | 0.60% | 0.07% | 46.4bp (38.4bp) | MF0002.1\_bZIP\_CREB/G-box-like\_subclass/Jaspar(0.794) More Information | Similar Motifs Found | motif file (matrix) |
| 69 \* | A C T G A G C T A C T G C G A T C G T A C G T A A C G T A C T G | 1e-9 | -2.239e+01 | 2.07% | 0.74% | 54.0bp (40.1bp) | TEAD4/MA0809.2/Jaspar(0.717) More Information | Similar Motifs Found | motif file (matrix) |
| 70 \* | A G T C G T A C C G T A A C G T A C T G A C G T G C T A A C T G | 1e-9 | -2.211e+01 | 1.81% | 0.58% | 51.9bp (48.7bp) | YY1/MA0095.2/Jaspar(0.689) More Information | Similar Motifs Found | motif file (matrix) |
| 71 \* | A C T G A C G T A C T G C G T A A G T C A G T C C G T A C T A G | 1e-9 | -2.173e+01 | 1.89% | 0.66% | 53.4bp (59.5bp) | PAX5/MA0014.3/Jaspar(0.760) More Information | Similar Motifs Found | motif file (matrix) |
| 72 \* | A G C T A T G C G C T A A T G C G A T C G C T A A G C T A G C T G C T A A G T C G A T C C G T A A T G C C T A G T A C G | 1e-9 | -2.161e+01 | 0.99% | 0.22% | 48.0bp (43.5bp) | NFAT:AP1(RHD,bZIP)/Jurkat-NFATC1-ChIP-Seq(Jolma\_et\_al.)/Homer(0.585) More Information | Similar Motifs Found | motif file (matrix) |
| 73 \* | A G T C T A G C C G T A G T A C C A T G A T C G A T G C A T G C A G T C A G C T A T C G A C T G A G T C T A C G G T A C G T C A A G T C G A C T A G T C G T A C G T A C G A T C G T A C A G T C T C A G A T C G G T A C A G T C A G T C C G A T | 1e-8 | -2.024e+01 | 0.43% | 0.04% | 52.4bp (0.0bp) | ZNF740/MA0753.2/Jaspar(0.483) More Information | Similar Motifs Found | motif file (matrix) |
| 74 \* | A C T G C G A T A G T C A C T G C T G A A C T G A C T G A C G T | 1e-8 | -1.864e+01 | 0.78% | 0.15% | 51.8bp (40.3bp) | RUNX3/MA0684.2/Jaspar(0.662) More Information | Similar Motifs Found | motif file (matrix) |
| 75 \* | C A T G T C G A C T G A T C A G A C T G C T A G C T G A C T A G C T A G T C A G C G T A C A T G T C A G G T C A C A T G C T A G C T G A T C A G C T A G T C G A C T G A C T A G C T A G T C A G C T G A | 1e-7 | -1.831e+01 | 0.52% | 0.07% | 52.7bp (74.6bp) | ZNF263/MA0528.2/Jaspar(0.533) More Information | Similar Motifs Found | motif file (matrix) |
| 76 \* | A C G T C G T A A C G T T A G C A G T C A T C G | 1e-7 | -1.826e+01 | 28.51% | 23.48% | 55.9bp (57.3bp) | SPDEF/MA0686.1/Jaspar(0.723) More Information | Similar Motifs Found | motif file (matrix) |
| 77 \* | C T A G C G T A A G C T A G T C A C G T C G T A C G T A A G T C | 1e-7 | -1.793e+01 | 1.08% | 0.30% | 51.0bp (50.8bp) | PB0194.1\_Zbtb12\_2/Jaspar(0.676) More Information | Similar Motifs Found | motif file (matrix) |
| 78 \* | C G T A C G T A A C T G A G T C C G T A A C T G A C G T A C G T | 1e-7 | -1.711e+01 | 1.16% | 0.35% | 50.7bp (50.9bp) | BMYB(HTH)/Hela-BMYB-ChIP-Seq(GSE27030)/Homer(0.731) More Information | Similar Motifs Found | motif file (matrix) |
| 79 \* | A C G T A G T C G T C A A G T C A C T G C G T A | 1e-6 | -1.496e+01 | 10.12% | 7.29% | 57.3bp (56.4bp) | Npas4(bHLH)/Neuron-Npas4-ChIP-Seq(GSE127793)/Homer(0.874) More Information | Similar Motifs Found | motif file (matrix) |
| 80 \* | C G T A A C G T C G T A A C G T A G T C A G T C C G T A A C T G | 1e-5 | -1.160e+01 | 1.29% | 0.53% | 51.7bp (48.2bp) | SD0003.1\_at\_AC\_acceptor/Jaspar(0.668) More Information | Similar Motifs Found | motif file (matrix) |
| 81 \* | C T A G G T C A A T C G G T A C C G A T A C T G C A G T A T G C A G T C A G T C C A T G A T C G C A T G A T C G C G A T A C T G A C T G T A C G A T C G C T G A | 1e-4 | -1.047e+01 | 0.56% | 0.15% | 58.9bp (58.3bp) | KLF4/MA0039.4/Jaspar(0.673) More Information | Similar Motifs Found | motif file (matrix) |
| 82 \* | A C T G C G T A A G T C C G T A A C G T C G T A A G T C A G T C | 1e-2 | -4.635e+00 | 0.22% | 0.08% | 60.2bp (54.5bp) | TEAD3/MA0808.1/Jaspar(0.772) More Information | Similar Motifs Found | motif file (matrix) |
| 83 \* | C T A G C T A G T C A G T C A G T C A G T C A G T C A G T C A G C T A G T C A G C T A G T C A G T C A G T C A G T C A G C T A G T C A G T C G A C T A G C T A G T C A G T C A G C T A G C T A G T C A G T C A G C T A G T C A G T C A G C T A G T C A G T C A G C T A G T C A G C T A G T C A G T C A G C T A G T C A G T C A G | 1e-1 | -3.623e+00 | 0.13% | 0.02% | 30.8bp (0.0bp) | PB0097.1\_Zfp281\_1/Jaspar(0.481) More Information | Similar Motifs Found | motif file (matrix) |
