# Supplementary material for: Global hypo-methylation in a proportion of glioblastoma enriched for an astrocytic signature is associated with increased invasion and altered immune landscape
Source: eLife. 2022 Nov 22;11:e77335. doi: 10.7554/eLife.77335 (PMC9681209; doi:10.7554/eLife.77335)
Supplement: Figure 2—source data 1. [file elife-77335-fig2-data1.zip › Figure_2_source_data_1/Figure_2A/knownResults.html]

/data/Blizard-MarinoLab/Nicola\_Pomella/Motifs\_James/JamesAnalysis\_Size\_200\_Motifs\_6to50/BG3/ - Homer Known Motif Enrichment Results


# Homer Known Motif Enrichment Results (/data/Blizard-MarinoLab/Nicola\_Pomella/Motifs\_James/JamesAnalysis\_Size\_200\_Motifs\_6to50/BG3/)

Homer *de novo* Motif Results  
Gene Ontology Enrichment Results  
Known Motif Enrichment Results (txt file)  
Total Target Sequences = 2322, Total Background Sequences = 3670

|  |  |  |  |  |  |  |  |  |  |  |  |
| --- | --- | --- | --- | --- | --- | --- | --- | --- | --- | --- | --- |
| Rank | Motif | Name | P-value | log P-pvalue | q-value (Benjamini) | # Target Sequences with Motif | % of Targets Sequences with Motif | # Background Sequences with Motif | % of Background Sequences with Motif | Motif File | SVG |
| 1 | T A C G C T A G A T G C G A T C G T A C A G T C C T A G A G T C A G T C A G T C G T A C A G T C | Sp1(Zf)/Promoter/Homer | 1e-4 | -1.089e+01 | 0.0082 | 49.0 | 2.11% | 40.0 | 1.09% | motif file (matrix) | svg |
| 2 | T G A C G T A C C G T A A C T G T G A C C G A T A C T G A T C G A G C T T A C G T C G A T A G C G T A C C G T A A T C G T G A C G C A T A C T G A C T G A T G C | Twist(bHLH)/HMLE-TWIST1-ChIP-Seq(Chang\_et\_al)/Homer | 1e-4 | -1.032e+01 | 0.0082 | 41.0 | 1.77% | 32.8 | 0.90% | motif file (matrix) | svg |
| 3 | T C G A C A T G C A T G A C G T A T G C T C G A C T G A A G C T T A C G T G C A G T A C G A T C A G C T A G T C | FXR(NR),IR1/Liver-FXR-ChIP-Seq(Chong\_et\_al.)/Homer | 1e-4 | -9.978e+00 | 0.0082 | 100.0 | 4.31% | 104.5 | 2.86% | motif file (matrix) | svg |
| 4 | T G A C C T A G A C T G T A G C C G A T A C T G A T G C C A T G A T C G A T C G A T C G T A G C C T G A T A G C G C T A A C T G C G T A A G C T C G T A C T G A | GATA:SCL(Zf,bHLH)/Ter119-SCL-ChIP-Seq(GSE18720)/Homer | 1e-4 | -9.769e+00 | 0.0082 | 29.0 | 1.25% | 20.3 | 0.56% | motif file (matrix) | svg |
| 5 | C A T G A G T C C T G A T G A C A T C G G A C T G T C A A G T C T A G C G A T C | HIF2a(bHLH)/785\_O-HIF2a-ChIP-Seq(GSE34871)/Homer | 1e-3 | -7.305e+00 | 0.0591 | 73.0 | 3.14% | 77.6 | 2.12% | motif file (matrix) | svg |
| 6 | T G C A G T A C C G T A A T C G A C T G A C G T C T A G C G A T T C G A A G T C | ZEB1(Zf)/PDAC-ZEB1-ChIP-Seq(GSE64557)/Homer | 1e-3 | -7.213e+00 | 0.0591 | 442.0 | 19.04% | 604.7 | 16.54% | motif file (matrix) | svg |
| 7 | T A C G G A C T T G A C C G T A A C G T G A T C G T C A C G T A A C G T A T G C C G T A G A C T | HOXA2(Homeobox)/mES-Hoxa2-ChIP-Seq(Donaldson\_et\_al.)/Homer | 1e-2 | -6.551e+00 | 0.0898 | 25.0 | 1.08% | 20.4 | 0.56% | motif file (matrix) | svg |
| 8 | T C G A T C A G T C G A A C T G C A T G A C G T A G T C C T G A | COUP-TFII(NR)/Artia-Nr2f2-ChIP-Seq(GSE46497)/Homer | 1e-2 | -6.180e+00 | 0.1138 | 338.0 | 14.56% | 458.3 | 12.53% | motif file (matrix) | svg |
| 9 | A C G T A G T C A G T C C G A T A C G T A C G T A C T G A C G T A T G C G A C T A C T G T A C G | Sox21(HMG)/ESC-SOX21-ChIP-Seq(GSE110505)/Homer | 1e-2 | -5.917e+00 | 0.1317 | 342.0 | 14.73% | 466.9 | 12.77% | motif file (matrix) | svg |
| 10 | A C G T G A C T T A G C C G T A C T G A C A T G C T A G G A C T G A T C C G T A | Nr5a2(NR)/Pancreas-LRH1-ChIP-Seq(GSE34295)/Homer | 1e-2 | -5.641e+00 | 0.1562 | 131.0 | 5.64% | 162.8 | 4.45% | motif file (matrix) | svg |
| 11 | C A T G G A C T T A C G G T C A G T A C G A T C G A C T A G C T A T C G T C G A T A C G T A G C | ERRg(NR)/Kidney-ESRRG-ChIP-Seq(GSE104905)/Homer | 1e-2 | -5.382e+00 | 0.1840 | 159.0 | 6.85% | 203.8 | 5.57% | motif file (matrix) | svg |
| 12 | G T A C G C A T C T G A C G T A G A C T A G C T C A T G T G C A C T G A A C G T G A C T C G T A | Prop1(Homeobox)/GHFT1-PROP1.biotin-ChIP-Seq(GSE77302)/Homer | 1e-2 | -5.231e+00 | 0.1961 | 180.0 | 7.75% | 234.2 | 6.40% | motif file (matrix) | svg |
| 13 | C G A T G A C T C G A T T C A G G A C T A C G T C A G T C T G A G A C T G A C T A G C T C G A T A C T G A T C G G T A C G C T A | NF1:FOXA1(CTF,Forkhead)/LNCAP-FOXA1-ChIP-Seq(GSE27824)/Homer | 1e-2 | -5.104e+00 | 0.2055 | 11.0 | 0.47% | 7.4 | 0.20% | motif file (matrix) | svg |
| 14 | T G A C C G T A C T G A A C T G A C T G G A C T G A T C T G C A G T A C T A C G | SF1(NR)/H295R-Nr5a1-ChIP-Seq(GSE44220)/Homer | 1e-2 | -4.759e+00 | 0.2695 | 82.0 | 3.53% | 98.6 | 2.70% | motif file (matrix) | svg |
| 15 | T C A G T A C G G A T C G T A C T C G A C G A T C T G A C G T A G C T A C G T A | Hoxd11(Homeobox)/ChickenMSG-Hoxd11.Flag-ChIP-Seq(GSE86088)/Homer | 1e-2 | -4.746e+00 | 0.2695 | 561.0 | 24.16% | 807.7 | 22.09% | motif file (matrix) | svg |
| 16 | A G C T T C G A G T A C T C G A A T G C A T G C G C A T A T C G A G T C A G C T | Snail1(Zf)/LS174T-SNAIL1.HA-ChIP-Seq(GSE127183)/Homer | 1e-2 | -4.687e+00 | 0.2695 | 203.0 | 8.74% | 271.7 | 7.43% | motif file (matrix) | svg |
| 17 | A T C G G A T C G C A T C G T A G T C A A C G T A T G C A G T C | CRX(Homeobox)/Retina-Crx-ChIP-Seq(GSE20012)/Homer | 1e-2 | -4.665e+00 | 0.2695 | 496.0 | 21.36% | 709.8 | 19.41% | motif file (matrix) | svg |
| 18 | C T A G A T C G G A C T A G T C A G T C A G T C G A C T C T G A A C T G C T A G A C T G C T G A | EBF(EBF)/proBcell-EBF-ChIP-Seq(GSE21978)/Homer | 1e-2 | -4.607e+00 | 0.2695 | 61.0 | 2.63% | 70.2 | 1.92% | motif file (matrix) | svg |
| 19 | C T A G A C T G C T A G T C A G T C A G T A C G C T A G A C T G | Maz(Zf)/HepG2-Maz-ChIP-Seq(GSE31477)/Homer | 1e-2 | -4.606e+00 | 0.2695 | 321.0 | 13.82% | 446.1 | 12.20% | motif file (matrix) | svg |
