# Supplementary material for: Global hypo-methylation in a proportion of glioblastoma enriched for an astrocytic signature is associated with increased invasion and altered immune landscape
Source: eLife. 2022 Nov 22;11:e77335. doi: 10.7554/eLife.77335 (PMC9681209; doi:10.7554/eLife.77335)
Supplement: Figure 2—source data 1. [file elife-77335-fig2-data1.zip › Figure_2_source_data_1/Figure_2C/homerResults/motif21.similar.html]

motif21

## Information for motif21

A
T
G
C
C
G
T
A
C
T
A
G
T
G
A
C
C
G
T
A
T
C
G
A
G
C
T
A
A
G
T
C
C
G
A
T
C
G
T
A
  
Reverse Opposite:  

G
C
A
T
G
C
T
A
T
C
A
G
C
G
A
T
A
G
C
T
C
G
A
T
A
C
T
G
G
A
T
C
G
C
A
T
T
A
C
G
  

|  |  |
| --- | --- |
| p-value: | 1e-14 |
| log p-value: | -3.318e+01 |
| Information Content per bp: | 1.655 |
| Number of Target Sequences with motif | 69.0 |
| Percentage of Target Sequences with motif | 5.87% |
| Number of Background Sequences with motif | 95.4 |
| Percentage of Background Sequences with motif | 1.97% |
| Average Position of motif in Targets | 100.3 +/- 55.0bp |
| Average Position of motif in Background | 97.6 +/- 59.4bp |
| Strand Bias (log2 ratio + to - strand density) | 0.0 |
| Multiplicity (# of sites on avg that occur together) | 1.04 |
| Motif File: | file (matrix) reverse opposite |

### Similar de novo motifs found

|  |  |  |  |  |  |  |  |
| --- | --- | --- | --- | --- | --- | --- | --- |
| Rank | Match Score | Redundant Motif | P-value | log P-value | % of Targets | % of Background | Motif file |
| 1 | 0.670 | C G A T C G A T G T C A A G C T G A C T C A G T C G A T A T C G T G C A C A G T | 1e-11 | -25.518801 | 4.00% | 1.25% | motif file (matrix) |
| 2 | 0.769 | G C A T A G C T C G T A A C T G A G C T A G C T C A G T C T A G | 1e-9 | -21.770298 | 12.24% | 7.15% | motif file (matrix) |
| 3 | 0.606 | A G T C C T G A A C T G A G T C A C G T A G T C C G T A A G T C | 1e-5 | -13.354581 | 2.21% | 0.76% | motif file (matrix) |
