# Supplementary material for: Global hypo-methylation in a proportion of glioblastoma enriched for an astrocytic signature is associated with increased invasion and altered immune landscape
Source: eLife. 2022 Nov 22;11:e77335. doi: 10.7554/eLife.77335 (PMC9681209; doi:10.7554/eLife.77335)
Supplement: Figure 2—source data 1. [file elife-77335-fig2-data1.zip › Figure_2_source_data_1/Figure_2C/homerResults/motif32.similar.html]

motif32

## Information for motif32

G
T
A
C
A
T
G
C
T
A
G
C
G
T
C
A
T
C
A
G
T
A
G
C
A
T
G
C
A
T
C
G
C
A
G
T
A
C
T
G
T
A
C
G
T
C
G
A
G
T
A
C
T
C
G
A
T
A
G
C
T
G
C
A
A
T
G
C
C
T
G
A
A
T
G
C
A
C
T
G
C
A
T
G
T
C
A
G
A
T
C
G
G
T
A
C
G
A
T
C
T
G
C
A
C
A
G
T
C
A
T
G
T
G
A
C
G
T
A
C
  
Reverse Opposite:  

C
A
T
G
A
C
T
G
G
T
A
C
G
T
C
A
A
C
G
T
C
A
T
G
C
A
T
G
A
T
G
C
A
G
T
C
G
A
T
C
T
G
A
C
T
A
C
G
G
A
C
T
A
T
C
G
A
C
G
T
A
T
C
G
A
G
C
T
A
C
T
G
A
G
C
T
A
T
G
C
A
G
T
C
G
T
C
A
T
A
G
C
T
A
C
G
A
C
T
G
A
G
T
C
A
C
G
T
A
T
C
G
T
A
C
G
A
C
T
G
  

|  |  |
| --- | --- |
| p-value: | 1e-12 |
| log p-value: | -2.951e+01 |
| Information Content per bp: | 1.565 |
| Number of Target Sequences with motif | 10.0 |
| Percentage of Target Sequences with motif | 0.85% |
| Number of Background Sequences with motif | 1.4 |
| Percentage of Background Sequences with motif | 0.03% |
| Average Position of motif in Targets | 98.9 +/- 49.2bp |
| Average Position of motif in Background | 134.6 +/- 6.8bp |
| Strand Bias (log2 ratio + to - strand density) | 0.8 |
| Multiplicity (# of sites on avg that occur together) | 1.40 |
| Motif File: | file (matrix) reverse opposite |

### Similar de novo motifs found

|  |  |  |  |  |  |  |  |
| --- | --- | --- | --- | --- | --- | --- | --- |
| Rank | Match Score | Redundant Motif | P-value | log P-value | % of Targets | % of Background | Motif file |
| 1 | 0.622 | T A G C C G A T G A T C T A G C T A G C C G T A T A C G A G T C G T C A T A C G T C A G C T A G T C A G T G C A G A T C G T C A T G C A G C T A A T G C T C G A A T G C A C T G T C A G A C G T T C A G T C G A T A C G G C T A T G A C C A T G T A G C C T A G A C T G T C G A C T G A | 1e-12 | -29.505524 | 0.85% | 0.01% | motif file (matrix) |
| 2 | 0.623 | T G A C C A T G T A G C A G T C A T G C T C G A T A C G G T A C T A G C A T C G C A G T A C T G G C T A A G C T A T G C T G A C G A T C T G A C T A C G T C A G A T G C T A C G T C A G A T C G A T C G C G T A A T G C T C A G A C T G A T C G T G A C G T C A A T C G T A C G C G T A A T C G A T G C A C T G T G A C T G A C | 1e-11 | -25.778830 | 0.77% | 0.01% | motif file (matrix) |
