# Supplementary material for: Global hypo-methylation in a proportion of glioblastoma enriched for an astrocytic signature is associated with increased invasion and altered immune landscape
Source: eLife. 2022 Nov 22;11:e77335. doi: 10.7554/eLife.77335 (PMC9681209; doi:10.7554/eLife.77335)
Supplement: Figure 2—source data 1. [file elife-77335-fig2-data1.zip › Figure_2_source_data_1/Figure_2C/homerResults/motif20.similar.html]

motif20

## Information for motif20

T
A
G
C
A
T
C
G
A
T
G
C
T
A
C
G
A
T
G
C
A
C
T
G
A
T
C
G
T
A
C
G
A
G
T
C
A
G
T
C
G
T
A
C
C
G
A
T
A
T
C
G
A
T
C
G
A
G
T
C
A
T
C
G
A
G
C
T
A
T
C
G
A
T
G
C
A
C
T
G
  
Reverse Opposite:  

A
G
T
C
T
A
C
G
A
T
G
C
T
C
G
A
A
T
G
C
C
T
A
G
A
T
G
C
A
T
G
C
C
G
T
A
C
A
T
G
A
C
T
G
T
A
C
G
A
T
G
C
T
A
G
C
T
G
A
C
T
A
C
G
A
G
T
C
A
T
C
G
A
T
G
C
A
T
C
G
  

|  |  |
| --- | --- |
| p-value: | 1e-14 |
| log p-value: | -3.333e+01 |
| Information Content per bp: | 1.652 |
| Number of Target Sequences with motif | 11.0 |
| Percentage of Target Sequences with motif | 0.94% |
| Number of Background Sequences with motif | 1.8 |
| Percentage of Background Sequences with motif | 0.04% |
| Average Position of motif in Targets | 92.3 +/- 50.2bp |
| Average Position of motif in Background | 80.6 +/- 0.5bp |
| Strand Bias (log2 ratio + to - strand density) | 1.4 |
| Multiplicity (# of sites on avg that occur together) | 1.64 |
| Motif File: | file (matrix) reverse opposite |

### Similar de novo motifs found

|  |  |  |  |  |  |  |  |
| --- | --- | --- | --- | --- | --- | --- | --- |
| Rank | Match Score | Redundant Motif | P-value | log P-value | % of Targets | % of Background | Motif file |
| 1 | 0.799 | A T G C A C T G A T C G C G T A A G T C A C T G A G T C G T A C C G A T C T A G A C T G A T C G A G C T G A T C G T A C | 1e-12 | -29.505524 | 0.85% | 0.04% | motif file (matrix) |
| 2 | 0.776 | T G C A C T A G T A G C A T G C A T C G A G T C T C A G A T G C T C A G A G C T C T A G A G T C A T G C T G C A C A G T A C T G A T C G A G T C T C A G C A G T A T G C G A T C A C T G A T G C T C A G | 1e-4 | -11.363045 | 1.11% | 0.25% | motif file (matrix) |
