# Supplementary material for: Global hypo-methylation in a proportion of glioblastoma enriched for an astrocytic signature is associated with increased invasion and altered immune landscape
Source: eLife. 2022 Nov 22;11:e77335. doi: 10.7554/eLife.77335 (PMC9681209; doi:10.7554/eLife.77335)
Supplement: Figure 2—figure supplement 1—source data 1. [file elife-77335-fig2-figsupp1-data1.zip › Figure_2_figure_supplement_1_source_data_1/Figure_2_figure_supplement_1_G_H/homerResults.html]

/data/Blizard-MarinoLab/Nicola\_Pomella/Motifs\_James/210119// - Homer de novo Motif Results


# Homer *de novo* Motif Results (/data/Blizard-MarinoLab/Nicola\_Pomella/Motifs\_James/210119//)

Known Motif Enrichment Results  
Gene Ontology Enrichment Results  
If Homer is having trouble matching a motif to a known motif, try copy/pasting the matrix file into
STAMP  
More information on motif finding results: HOMER
| Description of Results
| Tips
  
Total target sequences = 105  
Total background sequences = 605  
\* - possible false positive  

|  |  |  |  |  |  |  |  |  |
| --- | --- | --- | --- | --- | --- | --- | --- | --- |
| Rank | Motif | P-value | log P-pvalue | % of Targets | % of Background | STD(Bg STD) | Best Match/Details | Motif File |
| 1 | T G C A C G T A C T A G C T A G C A T G T A G C G T A C C G T A G C T A A G C T T G C A A C G T T A C G C G T A T C A G G C A T T G C A G T C A G T A C C T G A G C T A C T G A T G A C A G T C C G A T G C A T C A G T G T C A G T C A G A C T A T G C C T G A A C T G A T G C A G C T G T A C G C T A A C G T G A C T A T C G | 1e-26 | -6.145e+01 | 18.10% | 0.48% | 47.3bp (62.9bp) | PH0080.1\_Hoxd8/Jaspar(0.356) More Information | Similar Motifs Found | motif file (matrix) |
| 2 | A G T C A C G T A T C G C G A T A T G C T A G C A G T C C G T A A T C G A G T C C G T A A T C G | 1e-22 | -5.076e+01 | 13.33% | 0.28% | 57.2bp (0.0bp) | Zic1::Zic2/MA1628.1/Jaspar(0.705) More Information | Similar Motifs Found | motif file (matrix) |
| 3 | C T G A A C T G T C A G A G C T G C A T C A G T G T C A A C T G A G T C C A G T G A T C G A T C A C G T A T C G C G T A T A C G G A T C G T A C G A C T C A G T T A C G T G A C A T G C G T C A A G C T C G T A A T G C G T C A C G T A A T C G T G A C A G C T G T A C C G T A T G C A | 1e-22 | -5.076e+01 | 13.33% | 0.12% | 49.4bp (0.0bp) | ZNF322(Zf)/HEK293-ZNF322.GFP-ChIP-Seq(GSE58341)/Homer(0.404) More Information | Similar Motifs Found | motif file (matrix) |
| 4 | C T G A T G C A G A T C A T C G T G A C G T A C G T A C C T G A C A G T C A T G A C G T G C A T G A C T C G A T G A C T T G A C G T C A T C G A G T C A G A T C C T G A A G C T A T G C G A T C G C A T A C G T C A G T G C A T A G T C T G C A T A G C G T C A G T C A A G T C G T C A | 1e-22 | -5.076e+01 | 13.33% | 0.27% | 48.5bp (0.0bp) | SD0003.1\_at\_AC\_acceptor/Jaspar(0.439) More Information | Similar Motifs Found | motif file (matrix) |
| 5 | A G T C C G T A C T A G G A T C A G C T A G T C A C G T A G C T T A G C A G C T | 1e-20 | -4.623e+01 | 12.38% | 0.21% | 49.4bp (0.8bp) | MyoG(bHLH)/C2C12-MyoG-ChIP-Seq(GSE36024)/Homer(0.740) More Information | Similar Motifs Found | motif file (matrix) |
| 6 | A C T G A G T C G A T C A G T C A G T C A G T C G A C T A C T G C T G A T G C A G C A T G A C T A T C G T A G C T G C A C A T G T A G C A T C G G A T C G A T C T C G A C T G A A T C G G A T C G A T C G T C A G A T C G A T C T G A C G T C A | 1e-20 | -4.623e+01 | 12.38% | 0.29% | 50.7bp (55.5bp) | CTCFL/MA1102.2/Jaspar(0.453) More Information | Similar Motifs Found | motif file (matrix) |
| 7 | C T G A C T A G G A T C C G T A T G C A G C A T C G T A G C T A A G C T G A T C G T A C G T A C T C G A G C A T C G A T A G T C G T A C A G C T T C A G T C G A T C G A C T G A G C T A G A T C G T A C C G T A A G T C G C A T C G A T G A C T C G A T G A C T A G T C C G A T T A G C G A C T G T C A T C G A A C T G C G A T | 1e-20 | -4.623e+01 | 12.38% | 0.30% | 41.3bp (20.9bp) | PITX2/MA1547.1/Jaspar(0.367) More Information | Similar Motifs Found | motif file (matrix) |
| 8 | A G T C G A T C G T C A A C G T C G T A C A T G G T C A A G C T A G C T C T G A T C G A G C T A G T A C G A C T T G C A G A C T G A T C G C T A G C A T G A T C A G T C G C A T A G C T G C A T A T G C G C A T C G T A G C A T A C G T A G C T G A T C G C T A A C G T C A T G A G C T G A T C G A T C G A C T T C G A T G C A G T C A C G T A A C G T T A G C G C A T G C A T G C T A G C T A G C T A A G C T | 1e-20 | -4.623e+01 | 12.38% | 0.26% | 34.8bp (39.3bp) | PB0061.1\_Sox11\_1/Jaspar(0.399) More Information | Similar Motifs Found | motif file (matrix) |
| 9 | T A G C G T C A T G C A G T C A T G A C G T C A A T G C G T A C G T C A T C A G C G T A A C T G A G T C G A T C C T G A T G A C G C T A T A C G T G A C G T A C T G A C G A T C A C G T A G T C T A G C | 1e-18 | -4.180e+01 | 11.43% | 0.12% | 50.2bp (0.0bp) | POL003.1\_GC-box/Jaspar(0.471) More Information | Similar Motifs Found | motif file (matrix) |
| 10 | C T G A C G A T C T G A C G T A A C G T A C T G C A T G G A C T G A C T A C G T A C G T A G C T C T G A G C T A T G A C A G C T G A T C G T C A C G A T A C G T G C A T G C A T C G T A C A T G C T A G G C T A A C G T C G A T G T A C C T G A T A C G A G T C C A G T C G A T A G C T C T G A A G T C G T C A A C T G A G T C | 1e-18 | -4.180e+01 | 11.43% | 0.10% | 50.2bp (0.0bp) | ZNF652/MA1657.1/Jaspar(0.426) More Information | Similar Motifs Found | motif file (matrix) |
| 11 | A G T C A G T C G T A C C G T A C A T G A C T G G T A C A G T C G A T C G T A C G A C T A T G C | 1e-16 | -3.745e+01 | 10.48% | 0.12% | 47.8bp (0.0bp) | ZNF682/MA1599.1/Jaspar(0.682) More Information | Similar Motifs Found | motif file (matrix) |
| 12 | A C G T G A T C A C G T G T C A G A C T C G T A A C T G C G T A T G C A C T G A G C T A G A T C | 1e-16 | -3.745e+01 | 10.48% | 0.23% | 54.6bp (7.8bp) | ZBTB26/MA1579.1/Jaspar(0.677) More Information | Similar Motifs Found | motif file (matrix) |
| 13 | T G C A T C G A G A T C A C G T G T C A T G C A T G A C C G T A T G A C T G A C A C G T T A G C G T C A A C G T T A G C G T A C G A T C G C T A A T G C C G T A | 1e-16 | -3.745e+01 | 10.48% | 0.19% | 50.3bp (56.7bp) | ZNF263/MA0528.2/Jaspar(0.502) More Information | Similar Motifs Found | motif file (matrix) |
| 14 | G C T A C G T A C G A T C G A T A C T G A G T C G T C A G T A C G T C A C A T G T G C A C A T G C G A T C G T A C A G T C A T G A C T G G C T A G C T A A G T C G C A T A C T G G C A T C A T G C T G A G T A C G C A T A C T G C T G A T G C A C T A G C T G A C G A T T C G A T A C G C A G T C A T G A C G T A C G T A G C T | 1e-16 | -3.745e+01 | 10.48% | 0.00% | 48.7bp (0.0bp) | Mef2a(MADS)/HL1-Mef2a.biotin-ChIP-Seq(GSE21529)/Homer(0.382) More Information | Similar Motifs Found | motif file (matrix) |
| 15 | A C T G T C A G T A G C G T A C A C T G A T G C G T A C C T A G A T C G A G T C A T C G A T G C G C A T A G T C A C G T A T C G A T C G C A G T A T C G T C A G C T G A C T A G C T A G G A C T A C T G T A C G G A C T A C G T A C T G A G T C A G C T A C T G A G T C G A C T A G C T | 1e-15 | -3.498e+01 | 14.29% | 0.80% | 51.1bp (42.8bp) | ZNF528(Zf)/HEK293-ZNF528.GFP-ChIP-Seq(GSE58341)/Homer(0.472) More Information | Similar Motifs Found | motif file (matrix) |
| 16 | T G C A G A C T A G C T G C T A G C A T G C A T G A T C G C T A T C G A T G A C G C A T G A T C G T C A C T G A T G C A C G T A G T C A G T C A G A C T A C G T G A C T G C T A A G T C G T A C T C G A | 1e-14 | -3.362e+01 | 11.43% | 0.44% | 50.4bp (41.3bp) | PH0132.1\_Pax6/Jaspar(0.508) More Information | Similar Motifs Found | motif file (matrix) |
| 17 | C G A T A C G T A C T G A G T C A G T C G T A C G T C A T G C A T G C A G A C T G A C T G T A C G C T A T A G C G T C A G C T A T C A G T A G C G A C T C A G T T G A C A T G C A T G C G T A C G T A C A T G C G T A C C G A T A T C G G T A C A T G C G C A T G T A C A G T C T G A C | 1e-14 | -3.362e+01 | 11.43% | 0.40% | 49.7bp (26.5bp) | RREB1/MA0073.1/Jaspar(0.447) More Information | Similar Motifs Found | motif file (matrix) |
| 18 | G A T C C G T A C T G A T C G A T C A G G A T C A G T C A G C T A G T C A G T C | 1e-14 | -3.320e+01 | 9.52% | 0.33% | 57.6bp (41.2bp) | Sox9(HMG)/Limb-SOX9-ChIP-Seq(GSE73225)/Homer(0.648) More Information | Similar Motifs Found | motif file (matrix) |
| 19 | C G A T A C T G C A T G A C G T C G T A T A C G C T G A T A C G C G A T C A T G C T A G A G T C G C A T C A G T A C T G | 1e-14 | -3.320e+01 | 9.52% | 0.32% | 49.8bp (33.3bp) | NKX2-2/MA1645.1/Jaspar(0.609) More Information | Similar Motifs Found | motif file (matrix) |
| 20 | G T C A C G A T A C G T G C A T G C A T G T C A T A C G G T C A C T G A G T C A C G A T T C G A C G T A C T A G G A T C C G A T C G A T A T C G A G C T C G A T | 1e-14 | -3.320e+01 | 9.52% | 0.29% | 57.2bp (15.3bp) | Nr2e3/MA0164.1/Jaspar(0.496) More Information | Similar Motifs Found | motif file (matrix) |
| 21 | C G A T T A C G T C G A A C T G C G A T A G C T G C T A T C A G G T A C A G C T A G C T A G T C G C A T T A C G C G T A C A T G T G C A G A T C A G C T A C G T A C T G T C G A T A G C G C T A C A T G C T A G A T C G T C G A C T G A C A T G | 1e-14 | -3.320e+01 | 9.52% | 0.31% | 55.4bp (9.6bp) | Rarg(var.2)/MA0860.1/Jaspar(0.471) More Information | Similar Motifs Found | motif file (matrix) |
| 22 | A C T G T G A C T A G C C T A G A T C G G T A C A T C G T A C G A T G C C T A G A C G T T C A G A T C G A T G C A G T C G C T A A T C G T A C G T C A G G C T A T C A G A T C G A T G C A T C G A T C G T G C A C A T G A C T G T A G C A C T G | 1e-14 | -3.320e+01 | 9.52% | 0.31% | 48.8bp (48.7bp) | ZNF460/MA1596.1/Jaspar(0.546) More Information | Similar Motifs Found | motif file (matrix) |
| 23 | C G A T G T A C G C A T A G C T C G A T C A G T C A G T G C A T G T C A G C T A G T A C C G A T A G C T A G C T G A T C G A T C G A T C G C A T G A C T G A C T G T C A T C A G C A T G C A T G C G T A C G T A C G T A C G T A G A C T C G A T C G A T C T A G G C T A G A T C A C G T C T A G C T A G C G T A C T G A T C G A C T A G A G T C A G C T C G T A G C A T | 1e-14 | -3.320e+01 | 9.52% | 0.10% | 45.4bp (0.0bp) | IRF1/MA0050.2/Jaspar(0.445) More Information | Similar Motifs Found | motif file (matrix) |
| 24 | C G A T T A C G C T G A T A C G G C T A A G C T G C A T C T G A C T G A C T G A G T C A C G T A C G A T T C G A C A T G G C T A G C A T A C T G T C G A G A C T G T A C T G C A T C G A T A C G C T G A A T C G T C G A T G C A T C G A C T G A C G A T T A C G G C T A C A T G C G A T A G C T G A T C G A T C A C T G C T G A C T A G G C A T C G T A G C A T C A G T C T A G C T A G G C T A T G C A A C G T | 1e-14 | -3.320e+01 | 9.52% | 0.09% | 48.3bp (0.0bp) | PB0166.1\_Sox12\_2/Jaspar(0.445) More Information | Similar Motifs Found | motif file (matrix) |
| 25 | T C A G A T C G C A G T A C G T C G T A A T C G C G A T A C G T A G T C A G C T A T G C G T A C C G T A A T C G C T A G A T C G A T C G A T G C G A C T C A G T A T C G G A T C A C T G A C G T C A G T | 1e-13 | -2.997e+01 | 10.48% | 0.45% | 45.0bp (4.7bp) | KLF6/MA1517.1/Jaspar(0.540) More Information | Similar Motifs Found | motif file (matrix) |
| 26 | C G T A T G C A T G C A T G A C C G T A C A T G C G T A C G A T C A T G C A G T G T C A C T G A T G A C G C T A C T A G T C A G G T C A T C G A C A T G C T G A A G T C G C T A G T A C C G T A T C A G C T G A C T A G G C T A C T G A C G T A | 1e-13 | -2.997e+01 | 10.48% | 0.34% | 37.6bp (46.1bp) | IKZF1/MA1508.1/Jaspar(0.488) More Information | Similar Motifs Found | motif file (matrix) |
| 27 | C G T A C G T A C G T A C G A T A C G T A C G T A G T C A C G T C G T A A T G C | 1e-12 | -2.906e+01 | 8.57% | 0.31% | 40.6bp (33.5bp) | HSF2/MA0770.1/Jaspar(0.686) More Information | Similar Motifs Found | motif file (matrix) |
| 28 | A G T C C G T A A G C T C G A T A T G C G C A T A G T C C G T A T A G C A G T C A C G T T G A C | 1e-12 | -2.906e+01 | 8.57% | 0.20% | 49.9bp (0.0bp) | Eomes(T-box)/H9-Eomes-ChIP-Seq(GSE26097)/Homer(0.681) More Information | Similar Motifs Found | motif file (matrix) |
| 29 | G T A C A C G T C G A T A C T G G A T C G C A T C T G A T A C G A C G T G T C A A C G T G C A T | 1e-12 | -2.906e+01 | 8.57% | 0.19% | 55.0bp (0.0bp) | Duxbl(Homeobox)/NIH3T3-Duxbl.HA-ChIP-Seq(GSE119782)/Homer(0.619) More Information | Similar Motifs Found | motif file (matrix) |
| 30 | A C T G C T G A A C T G A T G C C G A T A T G C G A C T T A C G C A G T A C T G A T C G T A C G A C G T C A T G C T G A | 1e-12 | -2.906e+01 | 8.57% | 0.19% | 59.5bp (39.4bp) | Egr1(Zf)/K562-Egr1-ChIP-Seq(GSE32465)/Homer(0.612) More Information | Similar Motifs Found | motif file (matrix) |
| 31 | C A T G A C G T A G C T A C G T T A C G A C T G T G A C C G T A A C T G A T G C C G T A C T A G T A C G A C T G A T C G | 1e-12 | -2.906e+01 | 8.57% | 0.00% | 36.1bp (0.0bp) | Zic1::Zic2/MA1628.1/Jaspar(0.716) More Information | Similar Motifs Found | motif file (matrix) |
| 32 | A C G T A T C G A C G T A C T G A C G T C G A T C G T A G T C A A G T C A C G T A G T C A C G T C A T G C G A T C A T G | 1e-12 | -2.906e+01 | 8.57% | 0.28% | 45.7bp (25.0bp) | FoxD3(forkhead)/ZebrafishEmbryo-Foxd3.biotin-ChIP-seq(GSE106676)/Homer(0.621) More Information | Similar Motifs Found | motif file (matrix) |
| 33 | C G A T G T A C A G C T G T A C C G T A G C T A C A G T G T A C C G T A A G T C C G T A G T C A T C G A G A C T A C G T | 1e-12 | -2.906e+01 | 8.57% | 0.32% | 55.9bp (5.4bp) | RUNX1(Runt)/Jurkat-RUNX1-ChIP-Seq(GSE29180)/Homer(0.651) More Information | Similar Motifs Found | motif file (matrix) |
| 34 | T G C A A G T C A G T C A G T C C G T A A T C G C T G A C T A G A C T G C T A G A G T C G A C T A T G C A G T C G C T A | 1e-12 | -2.906e+01 | 8.57% | 0.00% | 47.6bp (0.0bp) | CTCFL/MA1102.2/Jaspar(0.656) More Information | Similar Motifs Found | motif file (matrix) |
| 35 | G T A C T G A C G A C T C A G T A G T C G T A C G T C A C G T A G A T C A T G C A G C T A C G T G T A C G C A T A G C T G A T C G A C T G C A T A T G C G T C A | 1e-12 | -2.906e+01 | 8.57% | 0.21% | 51.6bp (0.0bp) | E2F6/MA0471.2/Jaspar(0.527) More Information | Similar Motifs Found | motif file (matrix) |
| 36 | G T A C G A T C A T G C G T A C A T G C G T A C A G T C A T G C T G A C A T C G T G A C G T A C T A G C A G T C A G T C T G A C T C A G T A G C G T C A A T G C G T A C G A T C G T A C G T C A A T G C T G A C G A T C A G T C G C A T A T G C G T A C A T G C G A C T A G T C T A G C | 1e-12 | -2.906e+01 | 8.57% | 0.17% | 46.5bp (0.0bp) | PB0097.1\_Zfp281\_1/Jaspar(0.514) More Information | Similar Motifs Found | motif file (matrix) |
| 37 | C G T A C G A T T C G A T C A G C G A T C T A G A C G T A G C T C A G T A G C T C G T A G C A T G C T A C G T A G A T C G C A T T C G A T G A C C G A T C G A T C A G T G A T C G C A T C A T G C A T G C G T A C T G A C G A T G A T C C G T A A C T G T A C G G C T A A C G T C T G A G C A T G T A C C G T A C G T A C G A T C G T A C G T A G T A C G C T A C T G A C G A T C G T A C G T A A C T G G A C T | 1e-12 | -2.906e+01 | 8.57% | 0.00% | 52.0bp (0.0bp) | Cdx2(Homeobox)/mES-Cdx2-ChIP-Seq(GSE14586)/Homer(0.374) More Information | Similar Motifs Found | motif file (matrix) |
| 38 | C T G A A C G T C T A G G T A C A G T C A C T G A C T G A G T C | 1e-12 | -2.906e+01 | 8.57% | 0.27% | 56.5bp (42.2bp) | GCM2/MA0767.1/Jaspar(0.776) More Information | Similar Motifs Found | motif file (matrix) |
| 39 | C T G A A G T C A G T C A G T C A G C T C G T A A T C G A G T C G A T C A G T C G C A T A G T C A C G T A G T C A G T C C G T A A T C G G A T C A G C T G T A C | 1e-12 | -2.890e+01 | 11.43% | 0.60% | 55.6bp (34.4bp) | Maz(Zf)/HepG2-Maz-ChIP-Seq(GSE31477)/Homer(0.565) More Information | Similar Motifs Found | motif file (matrix) |
| 40 \* | A C G T C G T A A C G T C G A T A C T G C T A G C G T A C G T A A T G C C G T A | 1e-11 | -2.641e+01 | 9.52% | 0.47% | 58.5bp (6.8bp) | ZNF189(Zf)/HEK293-ZNF189.GFP-ChIP-Seq(GSE58341)/Homer(0.717) More Information | Similar Motifs Found | motif file (matrix) |
| 41 \* | C G T A C G A T C T G A A T G C C G T A G T A C G T C A G A T C G T A C A G T C G T C A C T A G | 1e-11 | -2.641e+01 | 9.52% | 0.48% | 44.1bp (35.4bp) | Ddit3::Cebpa/MA0019.1/Jaspar(0.643) More Information | Similar Motifs Found | motif file (matrix) |
| 42 \* | A G T C G T A C A G T C C G T A A C T G A C T G A C T G T C G A C A T G C T A G G T A C A C T G A C T G C G A T A C G T | 1e-11 | -2.641e+01 | 9.52% | 0.44% | 50.8bp (30.4bp) | EBF1(EBF)/Near-E2A-ChIP-Seq(GSE21512)/Homer(0.708) More Information | Similar Motifs Found | motif file (matrix) |
| 43 \* | G A T C A G T C A T G C G C T A A T C G C T G A A T C G G A T C A G T C A G C T T A G C T G C A T C A G A T G C A G T C A G T C G T C A A G T C G C T A T G A C A G T C G C A T A T G C G T C A G A C T | 1e-10 | -2.503e+01 | 7.62% | 0.00% | 47.3bp (0.0bp) | ZNF519(Zf)/HEK293-ZNF519.GFP-ChIP-Seq(GSE58341)/Homer(0.501) More Information | Similar Motifs Found | motif file (matrix) |
| 44 \* | C G A T T G A C G T C A C G A T G A C T G A C T A G C T G A C T A G C T G A C T A G C T C A G T C G A T A G C T G C A T T G C A A G T C G C A T C G A T C A G T A G T C C G A T T C G A G C A T A C G T A C G T G A C T A G C T C G T A C A T G G T C A G C T A G A C T G A C T A G C T A G C T C G A T G C T A A C G T C G T A A C G T G A T C G A C T C G A T G A T C | 1e-10 | -2.503e+01 | 7.62% | 0.21% | 34.9bp (23.1bp) | IRF1/MA0050.2/Jaspar(0.450) More Information | Similar Motifs Found | motif file (matrix) |
| 45 \* | C G T A G A C T C A G T C A G T A T C G A T G C G A C T A G C T G A C T A C G T A G T C C G T A A G T C G C T A T G A C T A G C A G C T A C G T A G C T T A G C C G A T T A C G C G T A C G T A C G T A A C G T A G T C C G T A C T A G G A T C G C T A G A C T G A C T A T C G G T A C C G A T C T A G T G C A C G T A A C T G G T A C C G T A G C T A A G C T T C G A | 1e-10 | -2.503e+01 | 7.62% | 0.00% | 57.8bp (0.0bp) | ONECUT1/MA0679.2/Jaspar(0.405) More Information | Similar Motifs Found | motif file (matrix) |
| 46 \* | A C G T A C G T A C G T C G T A G C A T A C G T C G A T A G T C C G T A A C T G | 1e-9 | -2.296e+01 | 8.57% | 0.47% | 35.5bp (3.4bp) | CDX2/MA0465.2/Jaspar(0.715) More Information | Similar Motifs Found | motif file (matrix) |
| 47 \* | A G T C G T A C G C A T A T C G C A G T A T G C G A C T G A T C G A T C G T A C T G A C G A C T | 1e-9 | -2.296e+01 | 8.57% | 0.44% | 68.6bp (72.6bp) | ZNF165(Zf)/WHIM12-ZNF165-ChIP-Seq(GSE65937)/Homer(0.626) More Information | Similar Motifs Found | motif file (matrix) |
| 48 \* | C T A G C G A T C G T A C G A T A G T C C G A T A G C T A G T C A C G T G A C T A C G T G A T C | 1e-9 | -2.296e+01 | 8.57% | 0.39% | 55.8bp (56.5bp) | Mecom/MA0029.1/Jaspar(0.638) More Information | Similar Motifs Found | motif file (matrix) |
| 49 \* | T A C G T A G C A G T C A C G T T G A C C G A T A C T G C G T A T A C G T G C A G A C T C T A G C T A G A C T G C T A G | 1e-9 | -2.296e+01 | 8.57% | 0.42% | 51.9bp (60.9bp) | TCFL2(HMG)/K562-TCF7L2-ChIP-Seq(GSE29196)/Homer(0.534) More Information | Similar Motifs Found | motif file (matrix) |
| 50 \* | T A C G C A T G T C A G G A T C A T C G T C A G C G T A C T A G A T C G A T C G G A T C A G C T A T C G A T C G T C A G T C A G T C G A T A C G A T C G C T A G A C T G G T A C T A G C G A C T A C T G T A C G T A C G C A T G A T G C C T A G A T C G A C T G T A C G T A G C G A T C T A G C C T A G C T A G T C G A T C A G A T C G A G T C G T A C G C A T A T C G A C G T C T A G C A T G T C A G T A G C | 1e-9 | -2.296e+01 | 8.57% | 0.46% | 40.1bp (19.4bp) | Sp2(Zf)/HEK293-Sp2.eGFP-ChIP-Seq(Encode)/Homer(0.421) More Information | Similar Motifs Found | motif file (matrix) |
| 51 \* | A G C T G T C A A C G T A C G T A C G T A G T C A G T C A G T C | 1e-9 | -2.189e+01 | 20.95% | 4.19% | 48.2bp (47.0bp) | RBPJ/MA1116.1/Jaspar(0.754) More Information | Similar Motifs Found | motif file (matrix) |
| 52 \* | C G A T A C T G A T G C A G C T C A G T A C T G C T G A A G C T T G C A T G A C G T C A C G T A | 1e-9 | -2.113e+01 | 6.67% | 0.29% | 55.1bp (68.5bp) | MEIS1(var.2)/MA1639.1/Jaspar(0.596) More Information | Similar Motifs Found | motif file (matrix) |
| 53 \* | A C T G C G T A A C T G A T C G C G T A C T G A C T A G C T G A G T A C C G A T A C G T C A G T | 1e-9 | -2.113e+01 | 6.67% | 0.24% | 62.7bp (0.0bp) | ETV4/MA0764.2/Jaspar(0.716) More Information | Similar Motifs Found | motif file (matrix) |
| 54 \* | A T G C C G T A A C G T A T C G C G T A A C T G A G T C A C G T A T C G A C G T A C T G G T A C C T G A A G T C A G C T | 1e-9 | -2.113e+01 | 6.67% | 0.24% | 49.8bp (0.0bp) | PB0026.1\_Gm397\_1/Jaspar(0.610) More Information | Similar Motifs Found | motif file (matrix) |
| 55 \* | G A T C A G C T A C G T A G C T A G T C A G T C G A T C A G T C G C T A A T G C A G T C A G C T A T C G A G T C G T C A | 1e-9 | -2.113e+01 | 6.67% | 0.15% | 43.0bp (0.0bp) | ZEB1/MA0103.3/Jaspar(0.658) More Information | Similar Motifs Found | motif file (matrix) |
| 56 \* | A C G T A C T G A G T C C G A T G T A C C T G A C G T A A C G T | 1e-9 | -2.113e+01 | 6.67% | 0.31% | 61.2bp (29.3bp) | CEBPD/MA0836.2/Jaspar(0.792) More Information | Similar Motifs Found | motif file (matrix) |
| 57 \* | C G T A G A C T A G T C C G T A A C T G C T A G A C T G A G T C A T G C G T C A | 1e-8 | -1.963e+01 | 7.62% | 0.47% | 64.5bp (64.4bp) | ZNF264(Zf)/HEK293-ZNF264.GFP-ChIP-Seq(GSE58341)/Homer(0.648) More Information | Similar Motifs Found | motif file (matrix) |
| 58 \* | C G A T C T A G C G T A A C T G C G T A G A T C A T G C C G A T A T C G G T C A G A C T C G T A | 1e-8 | -1.963e+01 | 7.62% | 0.45% | 50.9bp (40.7bp) | SIX2/MA1119.1/Jaspar(0.751) More Information | Similar Motifs Found | motif file (matrix) |
| 59 \* | A C T G A C T G A C T G C G T A A C G T A C G T A G T C A C G T A G T C A C G T | 1e-7 | -1.737e+01 | 5.71% | 0.29% | 55.9bp (35.3bp) | DPRX/MA1480.1/Jaspar(0.732) More Information | Similar Motifs Found | motif file (matrix) |
| 60 \* | C G A T A T C G T C G A A C G T C G A T A T C G G T A C A G T C C G A T A C T G A C T G G T A C | 1e-7 | -1.737e+01 | 5.71% | 0.31% | 48.4bp (0.0bp) | PBX1(Homeobox)/MCF7-PBX1-ChIP-Seq(GSE28007)/Homer(0.659) More Information | Similar Motifs Found | motif file (matrix) |
| 61 \* | A C G T C T A G A G T C C A T G A C G T A C G T C G T A A C T G C G T A C G T A A C T G A T G C | 1e-7 | -1.737e+01 | 5.71% | 0.09% | 72.1bp (0.0bp) | POL008.1\_DCE\_S\_I/Jaspar(0.665) More Information | Similar Motifs Found | motif file (matrix) |
| 62 \* | C G T A C G T A G A C T A T G C A G T C G T C A T C A G A C T G | 1e-7 | -1.687e+01 | 20.95% | 5.52% | 53.4bp (54.5bp) | PB0185.1\_Tcf1\_2/Jaspar(0.721) More Information | Similar Motifs Found | motif file (matrix) |
| 63 \* | C G T A T C A G T A G C C T G A C T A G A G T C T A C G C A T G A T G C A G T C | 1e-6 | -1.514e+01 | 8.57% | 0.98% | 46.0bp (40.2bp) | Ascl2/MA0816.1/Jaspar(0.677) More Information | Similar Motifs Found | motif file (matrix) |
| 64 \* | G T C A C T G A C A T G C G T A T G C A C G T A G T A C T G C A C T A G A T G C G T A C A G T C | 1e-5 | -1.378e+01 | 4.76% | 0.33% | 62.5bp (25.1bp) | PRDM4/MA1647.1/Jaspar(0.691) More Information | Similar Motifs Found | motif file (matrix) |
| 65 \* | T C G A T C A G T A G C C G T A G A T C A G T C G T C A A C G T A C T G A C T G C A G T A T C G | 1e-5 | -1.378e+01 | 4.76% | 0.20% | 34.3bp (0.0bp) | REST-NRSF(Zf)/Jurkat-NRSF-ChIP-Seq/Homer(0.626) More Information | Similar Motifs Found | motif file (matrix) |
| 66 \* | A C T G C G A T A G C T A G C T C A G T A C T G T G A C C G T A A T C G G T A C | 1e-5 | -1.335e+01 | 5.71% | 0.39% | 37.7bp (9.3bp) | NFATC2/MA0152.1/Jaspar(0.703) More Information | Similar Motifs Found | motif file (matrix) |
| 67 \* | A C G T A T G C A C G T C T G A C G T A C G T A | 1e-4 | -1.105e+01 | 42.86% | 24.08% | 58.5bp (55.2bp) | NFAT5/MA0606.1/Jaspar(0.694) More Information | Similar Motifs Found | motif file (matrix) |
| 68 \* | A C T G A C T G A C G T C G T A A C T G C G T A | 1e-4 | -1.046e+01 | 14.29% | 4.24% | 48.0bp (49.8bp) | PB0154.1\_Osr1\_2/Jaspar(0.673) More Information | Similar Motifs Found | motif file (matrix) |
| 69 \* | A C T G A T G C A C T G A C G T A C G T C G T A | 1e-4 | -1.046e+01 | 13.33% | 3.71% | 57.2bp (70.5bp) | ZNF652/HepG2-ZNF652.Flag-ChIP-Seq(Encode)/Homer(0.798) More Information | Similar Motifs Found | motif file (matrix) |
| 70 \* | A C T G A G T C A C G T A T G C C A T G C A G T A C T G C G T A A C T G A T G C | 1e-4 | -1.037e+01 | 3.81% | 0.22% | 59.6bp (4.1bp) | POL010.1\_DCE\_S\_III/Jaspar(0.695) More Information | Similar Motifs Found | motif file (matrix) |
| 71 \* | A C G T A C G T A C T G A C G T C T A G A C T G A C G T A G T C | 1e-4 | -9.474e+00 | 5.71% | 0.82% | 24.1bp (48.6bp) | RUNX2(Runt)/PCa-RUNX2-ChIP-Seq(GSE33889)/Homer(0.847) More Information | Similar Motifs Found | motif file (matrix) |
| 72 \* | C T A G C T A G A T C G T C A G A C T G A C G T A G T C A G C T | 1e-3 | -8.670e+00 | 9.52% | 2.46% | 57.5bp (56.7bp) | LRF(Zf)/Erythroblasts-ZBTB7A-ChIP-Seq(GSE74977)/Homer(0.751) More Information | Similar Motifs Found | motif file (matrix) |
| 73 \* | A C G T A C G T C G T A C A T G C G T A G C T A T G A C A G T C G C A T A G T C | 1e-3 | -7.562e+00 | 6.67% | 1.40% | 54.0bp (60.2bp) | PB0194.1\_Zbtb12\_2/Jaspar(0.675) More Information | Similar Motifs Found | motif file (matrix) |
