# Supplementary material for: Global hypo-methylation in a proportion of glioblastoma enriched for an astrocytic signature is associated with increased invasion and altered immune landscape
Source: eLife. 2022 Nov 22;11:e77335. doi: 10.7554/eLife.77335 (PMC9681209; doi:10.7554/eLife.77335)
Supplement: Figure 2—figure supplement 1—source data 1. [file elife-77335-fig2-figsupp1-data1.zip › Figure_2_figure_supplement_1_source_data_1/Figure_2_figure_supplement_1_G_H/knownResults.html]

/data/Blizard-MarinoLab/Nicola\_Pomella/Motifs\_James/210119/ - Homer Known Motif Enrichment Results


# Homer Known Motif Enrichment Results (/data/Blizard-MarinoLab/Nicola\_Pomella/Motifs\_James/210119/)

Homer *de novo* Motif Results  
Gene Ontology Enrichment Results  
Known Motif Enrichment Results (txt file)  
Total Target Sequences = 105, Total Background Sequences = 604

|  |  |  |  |  |  |  |  |  |  |  |  |
| --- | --- | --- | --- | --- | --- | --- | --- | --- | --- | --- | --- |
| Rank | Motif | Name | P-value | log P-pvalue | q-value (Benjamini) | # Target Sequences with Motif | % of Targets Sequences with Motif | # Background Sequences with Motif | % of Background Sequences with Motif | Motif File | SVG |
| 1 | A G T C A G T C C G A T A C G T A C G T A C T G A C G T A G C T A G T C A G T C | Sox4(HMG)/proB-Sox4-ChIP-Seq(GSE50066)/Homer | 1e-2 | -5.819e+00 | 1.0000 | 14.0 | 13.33% | 35.6 | 5.89% | motif file (matrix) | svg |
| 2 | C T G A C T A G A T C G G C A T A C T G G T A C A T G C C G T A A C T G G C T A A G T C C G T A | Tbox:Smad(T-box,MAD)/ESCd5-Smad2\_3-ChIP-Seq(GSE29422)/Homer | 1e-2 | -4.865e+00 | 1.0000 | 5.0 | 4.76% | 7.1 | 1.17% | motif file (matrix) | svg |
| 3 | A C G T A G T C A G T C C G A T A C G T A C G T A C T G A C G T A T G C G A C T A C T G T A C G | Sox21(HMG)/ESC-SOX21-ChIP-Seq(GSE110505)/Homer | 1e-2 | -4.850e+00 | 1.0000 | 22.0 | 20.95% | 75.0 | 12.38% | motif file (matrix) | svg |
| 4 | A C T G A C G T C A T G A T C G A T C G T G A C A C T G A T C G A T C G T G C A C T G A C G T A | E2F3(E2F)/MEF-E2F3-ChIP-Seq(GSE71376)/Homer | 1e-2 | -4.803e+00 | 1.0000 | 9.0 | 8.57% | 20.1 | 3.33% | motif file (matrix) | svg |
| 5 | C T A G A C T G T G C A G T C A A T G C C G T A A T C G A T G C A G T C C T A G | ZNF341(Zf)/EBV-ZNF341-ChIP-Seq(GSE113194)/Homer | 1e-2 | -4.703e+00 | 1.0000 | 15.0 | 14.29% | 44.6 | 7.37% | motif file (matrix) | svg |
| 6 | C T A G C T A G A G T C T C A G A C T G A C G T A C G T C T G A | MYB(HTH)/ERMYB-Myb-ChIPSeq(GSE22095)/Homer | 1e-2 | -4.644e+00 | 1.0000 | 23.0 | 21.90% | 80.5 | 13.30% | motif file (matrix) | svg |
