# Supplementary material for: Global hypo-methylation in a proportion of glioblastoma enriched for an astrocytic signature is associated with increased invasion and altered immune landscape
Source: eLife. 2022 Nov 22;11:e77335. doi: 10.7554/eLife.77335 (PMC9681209; doi:10.7554/eLife.77335)
Supplement: Figure 2—figure supplement 1—source data 1. [file elife-77335-fig2-figsupp1-data1.zip › Figure_2_figure_supplement_1_source_data_1/Figure_2_figure_supplement_1_G_H/homerResults/motif49.similar.html]

motif49

## Information for motif49

T
A
C
G
T
A
G
C
A
G
T
C
A
C
G
T
T
G
A
C
C
G
A
T
A
C
T
G
C
G
T
A
T
A
C
G
T
G
C
A
G
A
C
T
C
T
A
G
C
T
A
G
A
C
T
G
C
T
A
G
  
Reverse Opposite:  

A
G
T
C
G
T
A
C
A
G
T
C
G
A
T
C
C
G
T
A
A
C
G
T
A
T
G
C
A
C
G
T
T
A
G
C
C
G
T
A
A
C
T
G
T
G
C
A
C
T
A
G
A
T
C
G
A
G
T
C
  

|  |  |
| --- | --- |
| p-value: | 1e-9 |
| log p-value: | -2.296e+01 |
| Information Content per bp: | 1.657 |
| Number of Target Sequences with motif | 9.0 |
| Percentage of Target Sequences with motif | 8.57% |
| Number of Background Sequences with motif | 2.6 |
| Percentage of Background Sequences with motif | 0.42% |
| Average Position of motif in Targets | 113.1 +/- 51.9bp |
| Average Position of motif in Background | 87.2 +/- 60.9bp |
| Strand Bias (log2 ratio + to - strand density) | 1.2 |
| Multiplicity (# of sites on avg that occur together) | 1.11 |
| Motif File: | file (matrix) reverse opposite |

### Similar de novo motifs found

|  |  |  |  |  |  |  |  |
| --- | --- | --- | --- | --- | --- | --- | --- |
| Rank | Match Score | Redundant Motif | P-value | log P-value | % of Targets | % of Background | Motif file |
| 1 | 0.678 | A C T G C T A G A G T C A G T C A G C T G T C A C G A T A T C G C G T A A T C G | 1e-7 | -17.372432 | 5.71% | 0.17% | motif file (matrix) |
