# Supplementary material for: Global hypo-methylation in a proportion of glioblastoma enriched for an astrocytic signature is associated with increased invasion and altered immune landscape
Source: eLife. 2022 Nov 22;11:e77335. doi: 10.7554/eLife.77335 (PMC9681209; doi:10.7554/eLife.77335)
Supplement: Figure 2—figure supplement 1—source data 1. [file elife-77335-fig2-figsupp1-data1.zip › Figure_2_figure_supplement_1_source_data_1/Figure_2_figure_supplement_1_G_H/homerResults/motif6.similar.html]

motif6

## Information for motif6

A
C
T
G
A
G
T
C
G
A
T
C
A
G
T
C
A
G
T
C
A
G
T
C
G
A
C
T
A
C
T
G
C
T
G
A
T
G
C
A
G
C
A
T
G
A
C
T
A
T
C
G
T
A
G
C
T
G
C
A
C
A
T
G
T
A
G
C
A
T
C
G
G
A
T
C
G
A
T
C
T
C
G
A
C
T
G
A
A
T
C
G
G
A
T
C
G
A
T
C
G
T
C
A
G
A
T
C
G
A
T
C
T
G
A
C
G
T
C
A
  
Reverse Opposite:  

C
A
G
T
A
C
T
G
C
T
A
G
C
T
A
G
C
A
G
T
C
T
A
G
C
T
A
G
T
A
G
C
G
A
C
T
A
G
C
T
C
T
A
G
C
T
A
G
T
A
G
C
A
T
C
G
G
T
A
C
A
C
G
T
A
T
C
G
T
A
G
C
C
T
G
A
C
G
T
A
A
C
G
T
G
A
C
T
T
G
A
C
C
T
G
A
T
C
A
G
C
T
A
G
C
T
A
G
C
A
T
G
T
C
A
G
T
G
A
C
  

|  |  |
| --- | --- |
| p-value: | 1e-20 |
| log p-value: | -4.623e+01 |
| Information Content per bp: | 1.529 |
| Number of Target Sequences with motif | 13.0 |
| Percentage of Target Sequences with motif | 12.38% |
| Number of Background Sequences with motif | 1.8 |
| Percentage of Background Sequences with motif | 0.29% |
| Average Position of motif in Targets | 92.8 +/- 50.7bp |
| Average Position of motif in Background | 113.1 +/- 55.5bp |
| Strand Bias (log2 ratio + to - strand density) | 0.7 |
| Multiplicity (# of sites on avg that occur together) | 1.38 |
| Motif File: | file (matrix) reverse opposite |

### Similar de novo motifs found

|  |  |  |  |  |  |  |  |
| --- | --- | --- | --- | --- | --- | --- | --- |
| Rank | Match Score | Redundant Motif | P-value | log P-value | % of Targets | % of Background | Motif file |
