# Supplementary material for: Global hypo-methylation in a proportion of glioblastoma enriched for an astrocytic signature is associated with increased invasion and altered immune landscape
Source: eLife. 2022 Nov 22;11:e77335. doi: 10.7554/eLife.77335 (PMC9681209; doi:10.7554/eLife.77335)
Supplement: Figure 2—figure supplement 1—source data 1. [file elife-77335-fig2-figsupp1-data1.zip › Figure_2_figure_supplement_1_source_data_1/Figure_2_figure_supplement_1_G_H/homerResults/motif11.similar.html]

motif11

## Information for motif11

A
G
T
C
A
G
T
C
G
T
A
C
C
G
T
A
C
A
T
G
A
C
T
G
G
T
A
C
A
G
T
C
G
A
T
C
G
T
A
C
G
A
C
T
A
T
G
C
  
Reverse Opposite:  

T
A
C
G
C
T
G
A
A
C
T
G
C
A
T
G
C
T
A
G
A
C
T
G
A
G
T
C
G
T
A
C
C
G
A
T
A
C
T
G
A
C
T
G
A
C
T
G
  

|  |  |
| --- | --- |
| p-value: | 1e-16 |
| log p-value: | -3.745e+01 |
| Information Content per bp: | 1.753 |
| Number of Target Sequences with motif | 11.0 |
| Percentage of Target Sequences with motif | 10.48% |
| Number of Background Sequences with motif | 0.7 |
| Percentage of Background Sequences with motif | 0.12% |
| Average Position of motif in Targets | 106.0 +/- 47.8bp |
| Average Position of motif in Background | 154.0 +/- 0.0bp |
| Strand Bias (log2 ratio + to - strand density) | 0.0 |
| Multiplicity (# of sites on avg that occur together) | 1.09 |
| Motif File: | file (matrix) reverse opposite |

### Similar de novo motifs found

|  |  |  |  |  |  |  |  |
| --- | --- | --- | --- | --- | --- | --- | --- |
| Rank | Match Score | Redundant Motif | P-value | log P-value | % of Targets | % of Background | Motif file |
| 1 | 0.736 | C A G T C A T G C A T G C A T G A T C G A T G C G A T C C G A T C A T G C A T G A C T G A C G T A C G T A C G T A T G C | 1e-9 | -21.129976 | 6.67% | 0.29% | motif file (matrix) |
| 2 | 0.620 | G A T C C T G A T C A G C T A G A T G C T G A C G T C A A G T C C G A T A C T G | 1e-8 | -19.627761 | 7.62% | 0.44% | motif file (matrix) |
| 3 | 0.695 | A G T C A T C G A G T C C G T A A C T G C T G A A G T C A G T C A G T C A G T C | 1e-7 | -16.419503 | 6.67% | 0.40% | motif file (matrix) |
