# Supplementary material for: Global hypo-methylation in a proportion of glioblastoma enriched for an astrocytic signature is associated with increased invasion and altered immune landscape
Source: eLife. 2022 Nov 22;11:e77335. doi: 10.7554/eLife.77335 (PMC9681209; doi:10.7554/eLife.77335)
Supplement: Figure 2—figure supplement 1—source data 1. [file elife-77335-fig2-figsupp1-data1.zip › Figure_2_figure_supplement_1_source_data_1/Figure_2_figure_supplement_1_G_H/homerResults/motif56.similar.html]

motif56

## Information for motif56

A
C
G
T
A
C
T
G
A
G
T
C
C
G
A
T
G
T
A
C
C
T
G
A
C
G
T
A
A
C
G
T
  
Reverse Opposite:  

C
G
T
A
A
C
G
T
A
G
C
T
A
C
T
G
C
G
T
A
A
C
T
G
A
G
T
C
C
G
T
A
  

|  |  |
| --- | --- |
| p-value: | 1e-9 |
| log p-value: | -2.113e+01 |
| Information Content per bp: | 1.874 |
| Number of Target Sequences with motif | 7.0 |
| Percentage of Target Sequences with motif | 6.67% |
| Number of Background Sequences with motif | 1.9 |
| Percentage of Background Sequences with motif | 0.31% |
| Average Position of motif in Targets | 89.4 +/- 61.2bp |
| Average Position of motif in Background | 103.8 +/- 29.3bp |
| Strand Bias (log2 ratio + to - strand density) | 0.7 |
| Multiplicity (# of sites on avg that occur together) | 1.14 |
| Motif File: | file (matrix) reverse opposite |

### Similar de novo motifs found

|  |  |  |  |  |  |  |  |
| --- | --- | --- | --- | --- | --- | --- | --- |
| Rank | Match Score | Redundant Motif | P-value | log P-value | % of Targets | % of Background | Motif file |
