# Supplementary material for: Global hypo-methylation in a proportion of glioblastoma enriched for an astrocytic signature is associated with increased invasion and altered immune landscape
Source: eLife. 2022 Nov 22;11:e77335. doi: 10.7554/eLife.77335 (PMC9681209; doi:10.7554/eLife.77335)
Supplement: Figure 2—figure supplement 1—source data 1. [file elife-77335-fig2-figsupp1-data1.zip › Figure_2_figure_supplement_1_source_data_1/Figure_2_figure_supplement_1_G_H/homerResults/motif51.similar.html]

motif51

## Information for motif51

A
G
C
T
G
T
C
A
A
C
G
T
A
C
G
T
A
C
G
T
A
G
T
C
A
G
T
C
A
G
T
C
  
Reverse Opposite:  

C
T
A
G
A
C
T
G
A
C
T
G
C
G
T
A
C
G
T
A
C
G
T
A
A
C
G
T
C
T
G
A
  

|  |  |
| --- | --- |
| p-value: | 1e-9 |
| log p-value: | -2.189e+01 |
| Information Content per bp: | 1.931 |
| Number of Target Sequences with motif | 22.0 |
| Percentage of Target Sequences with motif | 20.95% |
| Number of Background Sequences with motif | 25.4 |
| Percentage of Background Sequences with motif | 4.19% |
| Average Position of motif in Targets | 106.3 +/- 48.2bp |
| Average Position of motif in Background | 107.6 +/- 47.0bp |
| Strand Bias (log2 ratio + to - strand density) | 1.3 |
| Multiplicity (# of sites on avg that occur together) | 1.09 |
| Motif File: | file (matrix) reverse opposite |

### Similar de novo motifs found

|  |  |  |  |  |  |  |  |
| --- | --- | --- | --- | --- | --- | --- | --- |
| Rank | Match Score | Redundant Motif | P-value | log P-value | % of Targets | % of Background | Motif file |
| 1 | 0.623 | T C A G A G C T C G T A A C G T A C G T A G T C G A T C A G T C G A C T A C T G | 1e-9 | -21.129976 | 6.67% | 0.27% | motif file (matrix) |
| 2 | 0.656 | G T C A A C G T A C G T C A T G A T G C G T A C G A T C G T A C C G T A A T C G | 1e-7 | -16.419503 | 6.67% | 0.40% | motif file (matrix) |
| 3 | 0.739 | A G T C A C G T A C G T A C G T A G T C A G T C | 1e-4 | -10.214472 | 23.81% | 10.23% | motif file (matrix) |
