# Supplementary figures and images for: Global hypo-methylation in a proportion of glioblastoma enriched for an astrocytic signature is associated with increased invasion and altered immune landscape
Source: eLife. 2022 Nov 22;11:e77335. doi: 10.7554/eLife.77335 (PMC9681209; doi:10.7554/eLife.77335)

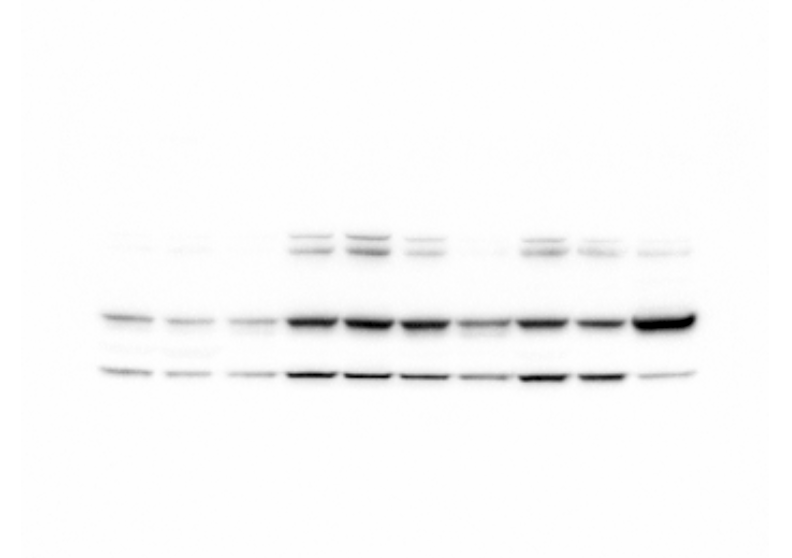

Supplement: Figure 4—source data 1. [file elife-77335-fig4-data1.zip › Figure_4_source_data_1/Figure_4_J/Gel1_Image2.tif]

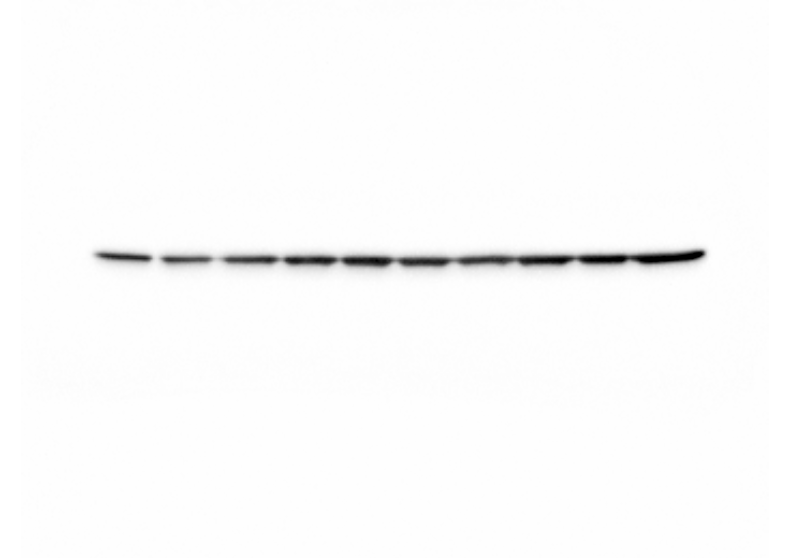

Supplement: Figure 4—source data 1. [file elife-77335-fig4-data1.zip › Figure_4_source_data_1/Figure_4_J/Gel1_Image3.tif]

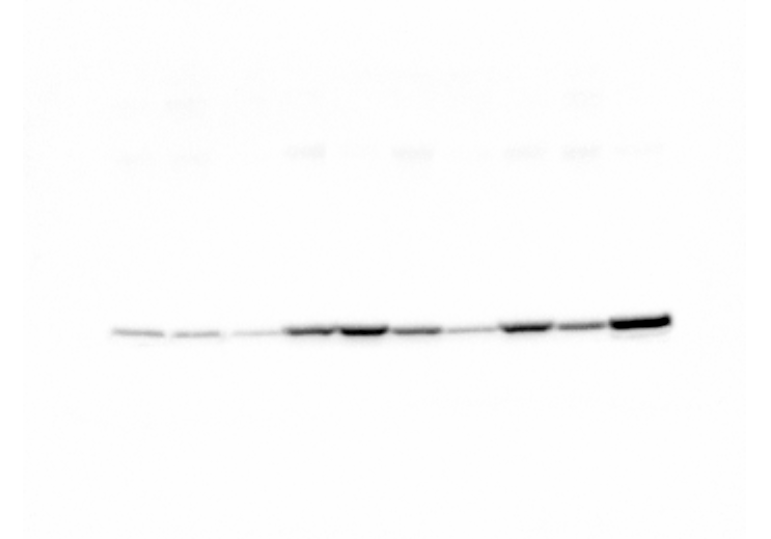

Supplement: Figure 4—source data 1. [file elife-77335-fig4-data1.zip › Figure_4_source_data_1/Figure_4_J/Gel1_Image1.tif]

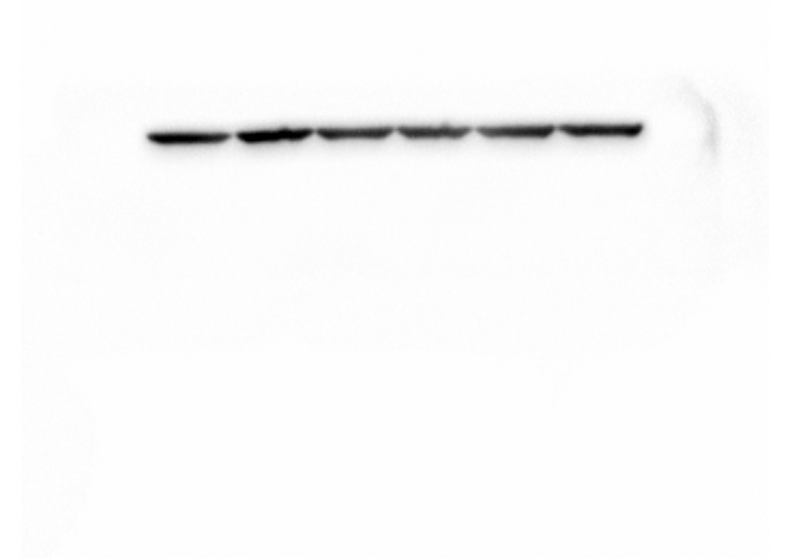

Supplement: Figure 4—source data 1. [file elife-77335-fig4-data1.zip › Figure_4_source_data_1/Figure_4_J/Gel4_Image1.tif]

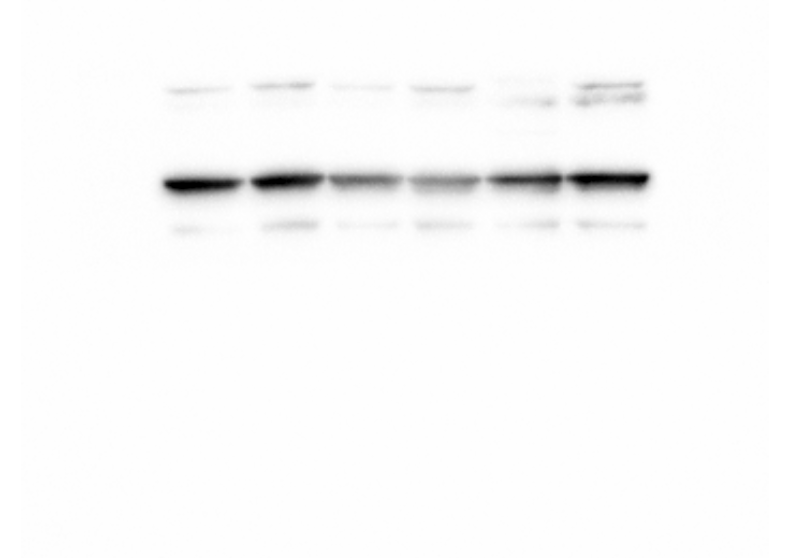

Supplement: Figure 4—source data 1. [file elife-77335-fig4-data1.zip › Figure_4_source_data_1/Figure_4_J/Gel4_Image2.tif]

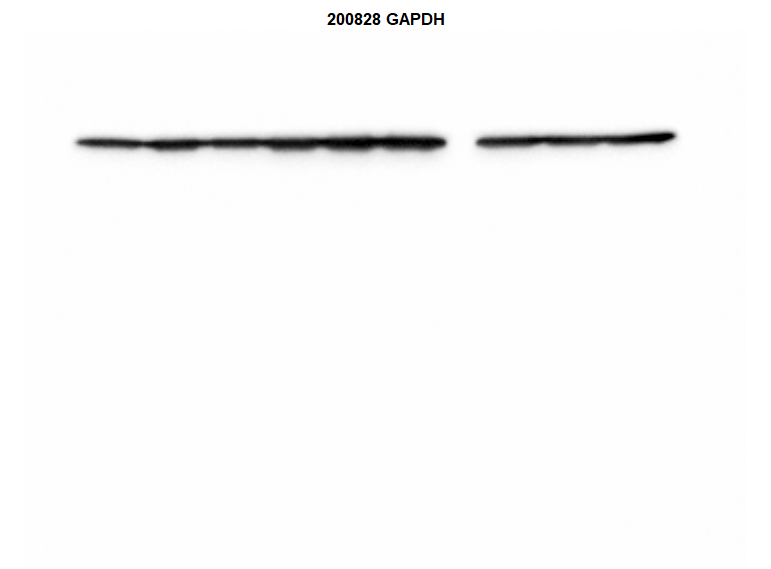

Supplement: Figure 4—source data 1. [file elife-77335-fig4-data1.zip › Figure_4_source_data_1/Figure_4_J/Figure_4_J_Gel3_Image1.tif]

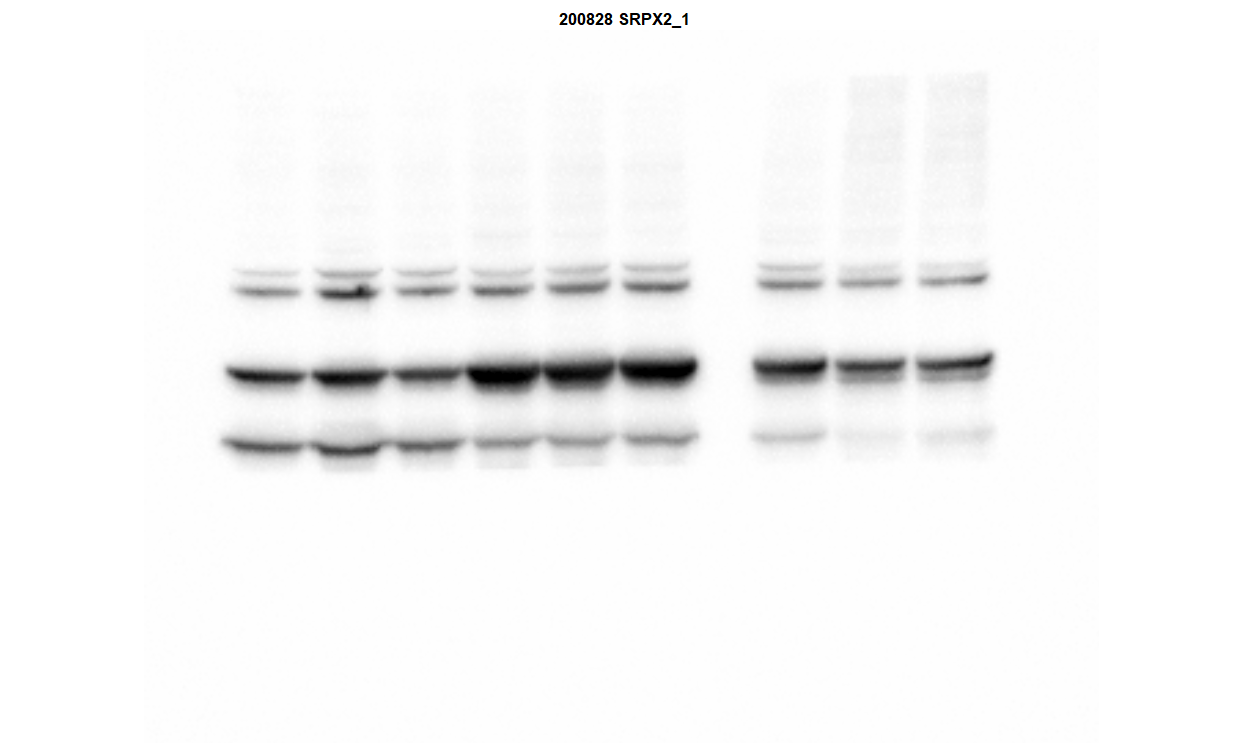

Supplement: Figure 4—source data 1. [file elife-77335-fig4-data1.zip › Figure_4_source_data_1/Figure_4_J/Figure_4_J_Gel3_Image2.tif]

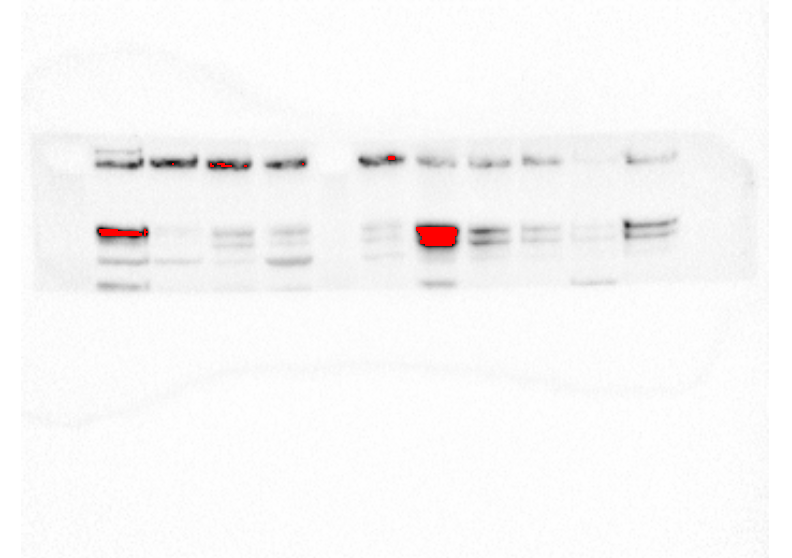

Supplement: Figure 4—source data 1. [file elife-77335-fig4-data1.zip › Figure_4_source_data_1/Figure_4_J/Gel2_Image2.tif]

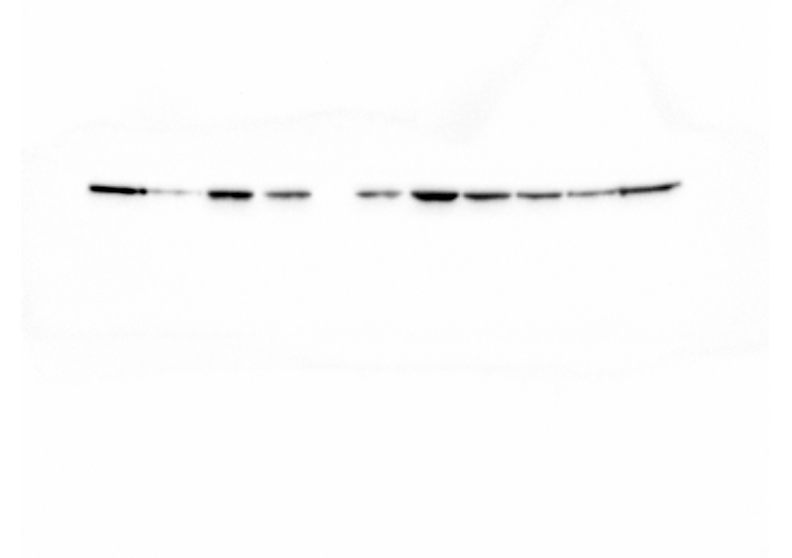

Supplement: Figure 4—source data 1. [file elife-77335-fig4-data1.zip › Figure_4_source_data_1/Figure_4_J/Gel2_Image1.tif]
