# Supplementary material for: Global hypo-methylation in a proportion of glioblastoma enriched for an astrocytic signature is associated with increased invasion and altered immune landscape
Source: eLife. 2022 Nov 22;11:e77335. doi: 10.7554/eLife.77335 (PMC9681209; doi:10.7554/eLife.77335)
Supplement: Figure 2—figure supplement 1—source data 1. [file elife-77335-fig2-figsupp1-data1.zip › Figure_2_figure_supplement_1_source_data_1/Figure_2_figure_supplement_1_G_H/homerResults/motif22.similar.html]

motif22

## Information for motif22

A
C
T
G
T
G
A
C
T
A
G
C
C
T
A
G
A
T
C
G
G
T
A
C
A
T
C
G
T
A
C
G
A
T
G
C
C
T
A
G
A
C
G
T
T
C
A
G
A
T
C
G
A
T
G
C
A
G
T
C
G
C
T
A
A
T
C
G
T
A
C
G
T
C
A
G
G
C
T
A
T
C
A
G
A
T
C
G
A
T
G
C
A
T
C
G
A
T
C
G
T
G
C
A
C
A
T
G
A
C
T
G
T
A
G
C
A
C
T
G
  
Reverse Opposite:  

G
T
A
C
A
T
C
G
T
G
A
C
G
T
A
C
A
C
G
T
T
A
G
C
T
A
G
C
T
A
C
G
T
A
G
C
A
G
T
C
C
G
A
T
A
G
T
C
A
G
T
C
T
A
G
C
C
G
A
T
T
C
A
G
T
A
C
G
T
A
G
C
A
G
T
C
T
G
C
A
G
A
T
C
A
T
C
G
A
T
G
C
T
A
G
C
C
A
T
G
A
T
G
C
G
A
T
C
A
T
C
G
A
C
T
G
T
G
A
C
  

|  |  |
| --- | --- |
| p-value: | 1e-14 |
| log p-value: | -3.320e+01 |
| Information Content per bp: | 1.508 |
| Number of Target Sequences with motif | 10.0 |
| Percentage of Target Sequences with motif | 9.52% |
| Number of Background Sequences with motif | 1.9 |
| Percentage of Background Sequences with motif | 0.31% |
| Average Position of motif in Targets | 119.1 +/- 48.8bp |
| Average Position of motif in Background | 59.5 +/- 48.7bp |
| Strand Bias (log2 ratio + to - strand density) | 0.5 |
| Multiplicity (# of sites on avg that occur together) | 1.40 |
| Motif File: | file (matrix) reverse opposite |

### Similar de novo motifs found

|  |  |  |  |  |  |  |  |
| --- | --- | --- | --- | --- | --- | --- | --- |
| Rank | Match Score | Redundant Motif | P-value | log P-value | % of Targets | % of Background | Motif file |
| 1 | 0.614 | C T A G A T G C A T C G C A T G A T C G A T G C A C T G T G A C G A T C G T C A C A G T C T A G C T A G T G A C C A G T A C T G C G T A C T A G T C A G C T G A C T A G T A C G A T G C C T A G A T C G A G C T A C G T A C G T A T G C G A C T | 1e-11 | -25.588096 | 11.43% | 0.73% | motif file (matrix) |
| 2 | 0.686 | T C G A T G A C G T A C A C T G A T C G A G T C A G C T A T G C A G T C C A G T A G C T T C A G A C T G A G T C A G T C C T A G C G T A C T A G A C T G T C G A T C A G T A C G A G T C A C T G A C T G | 1e-7 | -17.372432 | 5.71% | 0.33% | motif file (matrix) |
