# Supplementary material for: Global hypo-methylation in a proportion of glioblastoma enriched for an astrocytic signature is associated with increased invasion and altered immune landscape
Source: eLife. 2022 Nov 22;11:e77335. doi: 10.7554/eLife.77335 (PMC9681209; doi:10.7554/eLife.77335)
Supplement: Figure 2—figure supplement 1—source data 1. [file elife-77335-fig2-figsupp1-data1.zip › Figure_2_figure_supplement_1_source_data_1/Figure_2_figure_supplement_1_G_H/homerResults/motif36.similar.html]

motif36

## Information for motif36

G
T
A
C
G
A
T
C
A
T
G
C
G
T
A
C
A
T
G
C
G
T
A
C
A
G
T
C
A
T
G
C
T
G
A
C
A
T
C
G
T
G
A
C
G
T
A
C
T
A
G
C
A
G
T
C
A
G
T
C
T
G
A
C
T
C
A
G
T
A
G
C
G
T
C
A
A
T
G
C
G
T
A
C
G
A
T
C
G
T
A
C
G
T
C
A
A
T
G
C
T
G
A
C
G
A
T
C
A
G
T
C
G
C
A
T
A
T
G
C
G
T
A
C
A
T
G
C
G
A
C
T
A
G
T
C
T
A
G
C
  
Reverse Opposite:  

A
T
C
G
C
T
A
G
C
T
G
A
T
A
C
G
C
A
T
G
T
A
C
G
C
G
T
A
A
C
T
G
C
T
A
G
A
C
T
G
T
A
C
G
C
A
G
T
C
A
T
G
C
T
A
G
C
A
T
G
T
A
C
G
C
A
G
T
A
T
C
G
A
G
T
C
A
C
T
G
C
T
A
G
T
C
A
G
A
T
C
G
C
A
T
G
A
C
T
G
T
A
G
C
A
C
T
G
T
A
C
G
T
C
A
G
C
A
T
G
T
A
C
G
C
A
T
G
T
A
C
G
C
T
A
G
C
A
T
G
  

|  |  |
| --- | --- |
| p-value: | 1e-12 |
| log p-value: | -2.906e+01 |
| Information Content per bp: | 1.507 |
| Number of Target Sequences with motif | 9.0 |
| Percentage of Target Sequences with motif | 8.57% |
| Number of Background Sequences with motif | 1.1 |
| Percentage of Background Sequences with motif | 0.17% |
| Average Position of motif in Targets | 99.2 +/- 46.5bp |
| Average Position of motif in Background | 109.0 +/- 0.0bp |
| Strand Bias (log2 ratio + to - strand density) | 1.7 |
| Multiplicity (# of sites on avg that occur together) | 1.33 |
| Motif File: | file (matrix) reverse opposite |

### Similar de novo motifs found

|  |  |  |  |  |  |  |  |
| --- | --- | --- | --- | --- | --- | --- | --- |
| Rank | Match Score | Redundant Motif | P-value | log P-value | % of Targets | % of Background | Motif file |
| 1 | 0.753 | A G T C A G T C A T G C G T A C A T G C T G A C A T G C G T A C T A G C A G T C G T A C G A T C A T G C T G A C A T G C G T A C A G T C T G A C G A C T T A G C T G A C A G T C G A T C G A T C G A T C A G T C G A T C A G T C G A C T A G T C G T A C A T G C G T A C A G T C G A T C G T A C G A T C A T G C G T A C A G T C | 1e-5 | -13.778025 | 4.76% | 0.21% | motif file (matrix) |
| 2 | 0.740 | T G A C A T G C T G A C A G T C A G T C G T A C A G T C T G A C A G T C T G A C G A T C A G T C G T A C A G T C A T G C G A T C A T G C G T A C A T G C T G A C A T G C G T A C T A G C T G A C G T A C T G A C T A G C G T A C A G T C T G A C G A T C G A T C G T A C A T G C G T A C G A T C A T G C G A T C A G T C G T A C G A T C A T G C G A T C A T G C G T A C | 1e-4 | -10.374184 | 3.81% | 0.17% | motif file (matrix) |
| 3 | 0.737 | G T A C A T G C T G A C A G T C A G T C A G T C G A T C G A T C A G T C A G T C T G A C A G T C T G A C A T G C G A T C T G A C A T G C G T A C A G T C A G T C T G A C A G T C T G A C G A T C A T G C G T A C A T G C T G A C G A T C T G A C G A T C A G T C G T A C G A T C T A G C G A T C A T G C G T A C A G T C A T G C G A T C A T G C G T A C A T G C G A T C G A T C A T G C G T A C G A T C T G A C | 1e-4 | -10.374184 | 3.81% | 0.00% | motif file (matrix) |
