# Supplementary material for: Global hypo-methylation in a proportion of glioblastoma enriched for an astrocytic signature is associated with increased invasion and altered immune landscape
Source: eLife. 2022 Nov 22;11:e77335. doi: 10.7554/eLife.77335 (PMC9681209; doi:10.7554/eLife.77335)
Supplement: Figure 2—figure supplement 1—source data 1. [file elife-77335-fig2-figsupp1-data1.zip › Figure_2_figure_supplement_1_source_data_1/Figure_2_figure_supplement_1_G_H/homerResults/motif29.similar.html]

motif29

## Information for motif29

G
T
A
C
A
C
G
T
C
G
A
T
A
C
T
G
G
A
T
C
G
C
A
T
C
T
G
A
T
A
C
G
A
C
G
T
G
T
C
A
A
C
G
T
G
C
A
T
  
Reverse Opposite:  

C
G
T
A
C
G
T
A
C
A
G
T
T
G
C
A
A
T
G
C
G
A
C
T
C
G
T
A
C
A
T
G
T
G
A
C
C
G
T
A
G
T
C
A
C
A
T
G
  

|  |  |
| --- | --- |
| p-value: | 1e-12 |
| log p-value: | -2.906e+01 |
| Information Content per bp: | 1.703 |
| Number of Target Sequences with motif | 9.0 |
| Percentage of Target Sequences with motif | 8.57% |
| Number of Background Sequences with motif | 1.1 |
| Percentage of Background Sequences with motif | 0.19% |
| Average Position of motif in Targets | 120.0 +/- 55.0bp |
| Average Position of motif in Background | 102.0 +/- 0.0bp |
| Strand Bias (log2 ratio + to - strand density) | 0.3 |
| Multiplicity (# of sites on avg that occur together) | 1.00 |
| Motif File: | file (matrix) reverse opposite |

### Similar de novo motifs found

|  |  |  |  |  |  |  |  |
| --- | --- | --- | --- | --- | --- | --- | --- |
| Rank | Match Score | Redundant Motif | P-value | log P-value | % of Targets | % of Background | Motif file |
| 1 | 0.621 | A G C T T C A G A T G C A C G T A C G T A C G T T A G C A C G T C T A G C A T G C A T G C T A G C G A T C G A T C T G A | 1e-12 | -29.058555 | 8.57% | 0.32% | motif file (matrix) |
| 2 | 0.678 | C T G A C T A G T C G A T C A G T G C A G T C A C T A G T A G C G T C A T G C A C A T G C T G A C T A G T C A G C T G A | 1e-10 | -25.030493 | 7.62% | 0.28% | motif file (matrix) |
