# Supplementary material for: Global hypo-methylation in a proportion of glioblastoma enriched for an astrocytic signature is associated with increased invasion and altered immune landscape
Source: eLife. 2022 Nov 22;11:e77335. doi: 10.7554/eLife.77335 (PMC9681209; doi:10.7554/eLife.77335)
Supplement: Figure 2—figure supplement 1—source data 1. [file elife-77335-fig2-figsupp1-data1.zip › Figure_2_figure_supplement_1_source_data_1/Figure_2_figure_supplement_1_G_H/homerResults/motif58.similar.html]

motif58

## Information for motif58

C
G
A
T
C
T
A
G
C
G
T
A
A
C
T
G
C
G
T
A
G
A
T
C
A
T
G
C
C
G
A
T
A
T
C
G
G
T
C
A
G
A
C
T
C
G
T
A
  
Reverse Opposite:  

C
G
A
T
C
T
G
A
C
A
G
T
A
T
G
C
C
G
T
A
A
T
C
G
C
T
A
G
G
C
A
T
G
T
A
C
A
C
G
T
G
A
T
C
C
G
T
A
  

|  |  |
| --- | --- |
| p-value: | 1e-8 |
| log p-value: | -1.963e+01 |
| Information Content per bp: | 1.717 |
| Number of Target Sequences with motif | 8.0 |
| Percentage of Target Sequences with motif | 7.62% |
| Number of Background Sequences with motif | 2.7 |
| Percentage of Background Sequences with motif | 0.45% |
| Average Position of motif in Targets | 121.5 +/- 50.9bp |
| Average Position of motif in Background | 157.4 +/- 40.7bp |
| Strand Bias (log2 ratio + to - strand density) | 0.0 |
| Multiplicity (# of sites on avg that occur together) | 1.00 |
| Motif File: | file (matrix) reverse opposite |

### Similar de novo motifs found

|  |  |  |  |  |  |  |  |
| --- | --- | --- | --- | --- | --- | --- | --- |
| Rank | Match Score | Redundant Motif | P-value | log P-value | % of Targets | % of Background | Motif file |
