# Supplementary material for: Global hypo-methylation in a proportion of glioblastoma enriched for an astrocytic signature is associated with increased invasion and altered immune landscape
Source: eLife. 2022 Nov 22;11:e77335. doi: 10.7554/eLife.77335 (PMC9681209; doi:10.7554/eLife.77335)
Supplement: Figure 2—figure supplement 1—source data 1. [file elife-77335-fig2-figsupp1-data1.zip › Figure_2_figure_supplement_1_source_data_1/Figure_2_figure_supplement_1_G_H/homerResults/motif19.similar.html]

motif19

## Information for motif19

C
G
A
T
A
C
T
G
C
A
T
G
A
C
G
T
C
G
T
A
T
A
C
G
C
T
G
A
T
A
C
G
C
G
A
T
C
A
T
G
C
T
A
G
A
G
T
C
G
C
A
T
C
A
G
T
A
C
T
G
  
Reverse Opposite:  

T
G
A
C
G
C
T
A
C
G
T
A
T
C
A
G
A
G
T
C
G
A
T
C
G
C
T
A
A
T
G
C
G
A
C
T
A
T
G
C
A
C
G
T
T
G
C
A
G
A
T
C
T
G
A
C
G
C
T
A
  

|  |  |
| --- | --- |
| p-value: | 1e-14 |
| log p-value: | -3.320e+01 |
| Information Content per bp: | 1.665 |
| Number of Target Sequences with motif | 10.0 |
| Percentage of Target Sequences with motif | 9.52% |
| Number of Background Sequences with motif | 1.9 |
| Percentage of Background Sequences with motif | 0.32% |
| Average Position of motif in Targets | 106.2 +/- 49.8bp |
| Average Position of motif in Background | 54.7 +/- 33.3bp |
| Strand Bias (log2 ratio + to - strand density) | 0.0 |
| Multiplicity (# of sites on avg that occur together) | 1.00 |
| Motif File: | file (matrix) reverse opposite |

### Similar de novo motifs found

|  |  |  |  |  |  |  |  |
| --- | --- | --- | --- | --- | --- | --- | --- |
| Rank | Match Score | Redundant Motif | P-value | log P-value | % of Targets | % of Background | Motif file |
| 1 | 0.635 | G C T A A T G C G T A C G C T A C G T A A T G C A G C T A T C G A C G T A G T C | 1e-7 | -16.480014 | 12.38% | 1.85% | motif file (matrix) |
