# Supplementary material for: Global hypo-methylation in a proportion of glioblastoma enriched for an astrocytic signature is associated with increased invasion and altered immune landscape
Source: eLife. 2022 Nov 22;11:e77335. doi: 10.7554/eLife.77335 (PMC9681209; doi:10.7554/eLife.77335)
Supplement: Transparent reporting form [file elife-77335-transrepform1.docx]

***eLife’s* transparent reporting form**

We encourage authors to provide detailed information *within their submission* to facilitate the interpretation and replication of experiments. Authors can upload supporting documentation to indicate the use of appropriate reporting guidelines for health-related research (see [EQUATOR Network](http://www.equator-network.org/%20)), life science research (see the [BioSharing Information Resource](https://biosharing.org/)), or the [ARRIVE guidelines](http://www.plosbiology.org/article/info:doi/10.1371/journal.pbio.1000412) for reporting work involving animal research. Where applicable, authors should refer to any relevant reporting standards documents in this form.

If you have any questions, please consult our Journal Policies and/or contact us: [editorial@elifesciences.org](mailto:editorial@elifesciences.org).

**Sample-size estimation**

- You should state whether an appropriate sample size was computed when the study was being designed
- You should state the statistical method of sample size computation and any required assumptions
- If no explicit power analysis was used, you should describe how you decided what sample (replicate) size (number) to use

Please outline where this information can be found within the submission (e.g., sections or figure legends), or explain why this information doesn’t apply to your submission:

No sample size/power analysis was used. Reasoning:

For the in-vitro generation of non neoplastic primary iAPCs, iOPCs, iNSCs from iPSCs, that were used for DNA methylation and RNAseq and comparison with patient matched GICs in this study, no power analysis was used to determine the number of replicates for each cell line/type. Instead the sample size of 2 replicates per patient cell line/type was determined based on the feasibility of producing all lines in-vitro.

For 2 biological replicates of iNSCs, two independent differentiations were performed from the same iPSC clone. For 2 biological replicates of iAPCs and iOPCs, two independent differentiations were performed from the same iNSC line. This is detailed in the Methods section.

In order to match the number of replicates of non neoplastic primary patient cell lines (iAPCs, iOPCs, iNSCs), 2 biological replicates of GIC lines derived from the same patient were used, in this case different passages were considered biological replicates. This is detailed in the Methods section.

All other datasets used were publicly available so sample size was already determined.

**Replicates**

- You should report how often each experiment was performed
- You should include a definition of biological versus technical replication
- The data obtained should be provided and sufficient information should be provided to indicate the number of independent biological and/or technical replicates
- If you encountered any outliers, you should describe how these were handled
- Criteria for exclusion/inclusion of data should be clearly stated
- High-throughput sequence data should be uploaded before submission, with a private link for reviewers provided (these are available from both GEO and ArrayExpress)

Please outline where this information can be found within the submission (e.g., sections or figure legends), or explain why this information doesn’t apply to your submission:

The number of biological replicates performed for each experiment is reported in the figure legends where relevant and is indicated by “N = x” in brackets.

Specific details for each experiment/assay on what was deemed to be a biological or technical replicate and the number of each of these replicates performed can be found in the Methods section, under specific assay headings.

All raw data including the replicates can be found in the source files.

If any samples were included or excluded for any reason during the study - this is described and the reasoning provided in the Results section.

No outliers were identified or excluded in this study.

All accession numbers / links for publicly available datasets used in this study are listed in the Methods section under the heading Data availability.

New sequencing data produced during this study, namely RNAseq and DNA methylation array data of iAPCs, iOPCs and some iNSCs is available as a private GEO submission. Instructions on how reviewers can access this data is available below:

---

To review GEO accession GSE196418:

Go to <https://www.ncbi.nlm.nih.gov/geo/query/acc.cgi?acc=GSE196418>

Enter token mjefsyqizhqxlml into the box

---

---

To review GEO accession GSE196339:

Go to <https://www.ncbi.nlm.nih.gov/geo/query/acc.cgi?acc=GSE196339>

Enter token kfcxweewptchjeb into the box

---

These 2 GEO submissions will be made publicly available upon final acceptance of the paper for publication.

**Statistical reporting**

- Statistical analysis methods should be described and justified
- Raw data should be presented in figures whenever informative to do so (typically when N per group is less than 10)
- For each experiment, you should identify the statistical tests used, exact values of N, definitions of center, methods of multiple test correction, and dispersion and precision measures (e.g., mean, median, SD, SEM, confidence intervals; and, for the major substantive results, a measure of effect size (e.g., Pearson's r, Cohen's d)
- Report exact p-values wherever possible alongside the summary statistics and 95% confidence intervals. These should be reported for all key questions and not only when the p-value is less than 0.05.

Please outline where this information can be found within the submission (e.g., sections or figure legends), or explain why this information doesn’t apply to your submission:

The statistical methods used during analysis are described in the figure legends where relevant.

The Methods section contains further detailed descriptions of the statistical methods used for certain analyses and bioinformatic analyses.

Exact P-values are reported in the main text of the Results section and statistical significance is summarized/indicated using asterixis in the figure panels. The significance meanings of the number of asterixis is reported in the Methods section under the heading Statistical analysis and graphs.

(For large datasets, or papers with a very large number of statistical tests, you may upload a single table file with tests, Ns, etc., with reference to sections in the manuscript.)

**Group allocation**

- Indicate how samples were allocated into experimental groups (in the case of clinical studies, please specify allocation to treatment method); if randomization was used, please also state if restricted randomization was applied
- Indicate if masking was used during group allocation, data collection and/or data analysis

Please outline where this information can be found within the submission (e.g., sections or figure legends), or explain why this information doesn’t apply to your submission:

During this study some patient-derived GIC lines were segregated into different groups (e.g. bias, non-bias, or enriched, non-enriched, or bias/enriched, non-bias/non-enriched). Detailed descriptions of the numerical thresholds and strategies used to determine which patient-derived GICs were allocated to each group can be found in the Results section.

**Additional data files (“source data”)**

- We encourage you to upload relevant additional data files, such as numerical data that are represented as a graph in a figure, or as a summary table
- Where provided, these should be in the most useful format, and they can be uploaded as “Source data” files linked to a main figure or table
- Include model definition files including the full list of parameters used
- Include code used for data analysis (e.g., R, MatLab)
- Avoid stating that data files are “available upon request”

Please indicate the figures or tables for which source data files have been provided:

All files/folders referred to below are contained within the file eLife_Source_Files.zip

Source data for the figures listed below are available in the file named All_Figures_Source_File.xlsx:

Figure 1 B, E, G

Figure S1 H

Figure 2 F, G, H

Figure S2 A, C, D

Figure 3 A, C, D, E, F, G, H

Figure S3 A, B, C, D, G, H, I, J

Figure 4 C, E, F, G, H, J, K, L

Figure S4 C, E, F

Figure 5 A, B, G-O

Figure S5 A, B, C-H

Source data for the figures listed below are available in the folder named Figure_1A_1H_S1B-E_source, in this folder is also a markdown file named meta.md that contains descriptions of each of the files within this folder. All files in the folder are also labelled with the figure to which they correspond:

Figure 1 A, H

Figure S1 B-E

Source data for Figure S1H is available in the file named Figure_S1H_source.xlsx this file contains the lists of differentially expressed miRNAs, plus statistics, for all patient comparisons.

Source data for the figures listed below are available in the folder named Figure_2A-C_2I-J_S2G-H_source, this folder contains further subfolders with named by the figure to which they correspond:

Figure 2 A, B, C, I, J

Figure S2 G, H

All raw images for immunocytochemistry (ICC) of iAPCs from Figure S2B are available in the folder named Figure_S2B_iAPC_ICC_40x_images, images are named by the part of the figure panel to which they correspond e.g. bottom_left

Source data for Figure 4D is available in the folder named Figure_4D_Gene_Lists. This folder also contains a file named meta.md that contains descriptions of the folders and files within Figure_4D_Gene_Lists.

Source data and raw images for western blots (Figure 4 I, J and S4D) can be found in the folder named Figure_4I-J_S4D_source. This folder contains raw images of western blots actually shown in Figure 4J and S4D (labelled with the figure name to which they correspond). The folder also contains other raw images of western blot membranes of replicates that are not shown in the figures. The folder contains an excel file named Figure_4J_S4D_source.xlsx that contains the images annotated – highlighting bands of interest, labelling of lanes/samples, and the quantification values.

Heatmap/dendrograms, PCAs, UMAPs were produced from sequencing data, therefore sequencing data are the source files for these figures. Standard bioinformatic pipelines and tools have been used for data analysis during this study. Detailed descriptions of the tools/models used and the parameters used for key functions are detailed in the Methods so that these analyses can be recreated.
